# Helical Dispiroindeno[2,1‑*c*]fluorenes Possessing Planar Chirality: Synthesis and Chiroptical Properties

**DOI:** 10.1021/acs.joc.5c02008

**Published:** 2025-12-11

**Authors:** Marko Bogomolec, Lucia Feriancová, Jhon Sebastian Oviedo Ortiz, Jeanne Crassous, Ivana Císařová, Robert Gyepes, Timothée Cadart, Martin Kotora

**Affiliations:** † Department of Organic Chemistry, Faculty of Science, 112302 Charles University, Hlavova 8, Praha 2, Prague 128 00, Czech Republic; ‡ Institut des Sciences Chimiques de Rennes, 27079 University of Rennes, CNRS, ISCR, UMR 6226, Rennes F-35000, France; § Department of Inorganic Chemistry, Faculty of Science, 37740 Charles University, Hlavova 8, Praha 2, Prague 128 00, Czech Republic

## Abstract

Syntheses of enantioenriched helical dispiroindeno­[2,1-*c*]­fluorenes (DSIF) possessing one or two pCp moieties are presented. These are based on the conversion of (*S*_p_)- and (*R*_p_) pCp-carbaldehydes **1** to triynediols **2** and **6** that were converted to enantioenriched DSIFs **4** and **8** via a catalytic [2+2+2] cyclotrimerization, oxidation, and spirocyclization sequence. Photophysical properties were measured, and compounds **4** and **8** are highly fluorescent in the violet-blue light region with |*g*_lum_| values in the order of 10^–4^ and 10^–3^, and *B*_CPL_ values 1.1 and 7.6, respectively.

## Graphical abstract

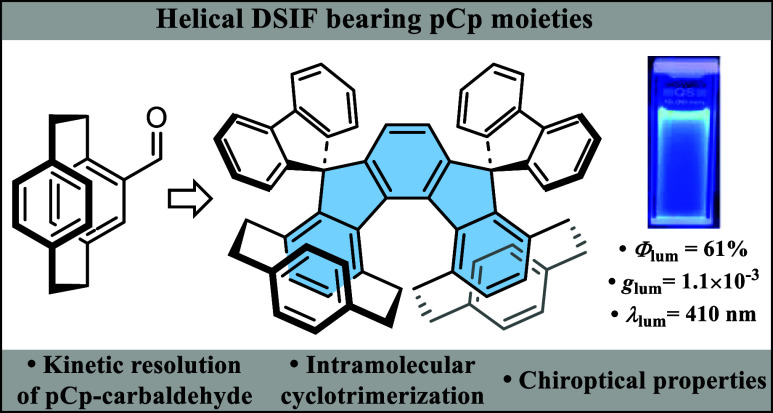

First synthesized by pyrolysis of *p*-xylene in 1949, [2.2]­paracyclophane (pCp) has gained more and more attention over the last two decades.^1^ pCp has a three-dimensional structure consisting of two overlapping, slightly curved benzene rings held in close proximity (∼3.09 Å) by two strained ethylene bridges (∼2.78 Å). Indeed, such a specific motif has found applications in various fields;^2^ in particular, chiral pCp-based substances^3^ such as chiral ligands,^4^ organocatalysts,^5^ and others^6^ have found wide utility in different areas of organic chemistry and related fields (Figure 1). Most importantly, pCp and its derivatives—driven by their chemical, physical, and topological properties—have made their way into the design of new CPL active compounds.^7^ Especially active in this area have been Morisaki et al., who have synthesized a huge variety of CPL active substances, in which the pCp moiety has played the central role.^8^ Alongside them, others such as Bräse etc., have also been involved.^9^ Despite considerable effort in this direction, only a handful of examples deal with the pCp unit embedded in some kind of a helical scaffold (e.g., phenanthrene,^10^ benzophenanthrene,^11^ [6]­helicene,^12^ and helicenophanes^13^ (Figure 1)). Worth mentioning is that this fragment has also been found as the central element in indeno­[1,2-*b*]­fluorenes^14^ or [2.2]­fluorenophanes.^15^ One example of dihydrohelicenophanes, with pCp unit as part of the 6,7-dihydroindeno­[2,1-*c*]­fluorene scaffold, has been reported as well.^16^

**Figure 1:**
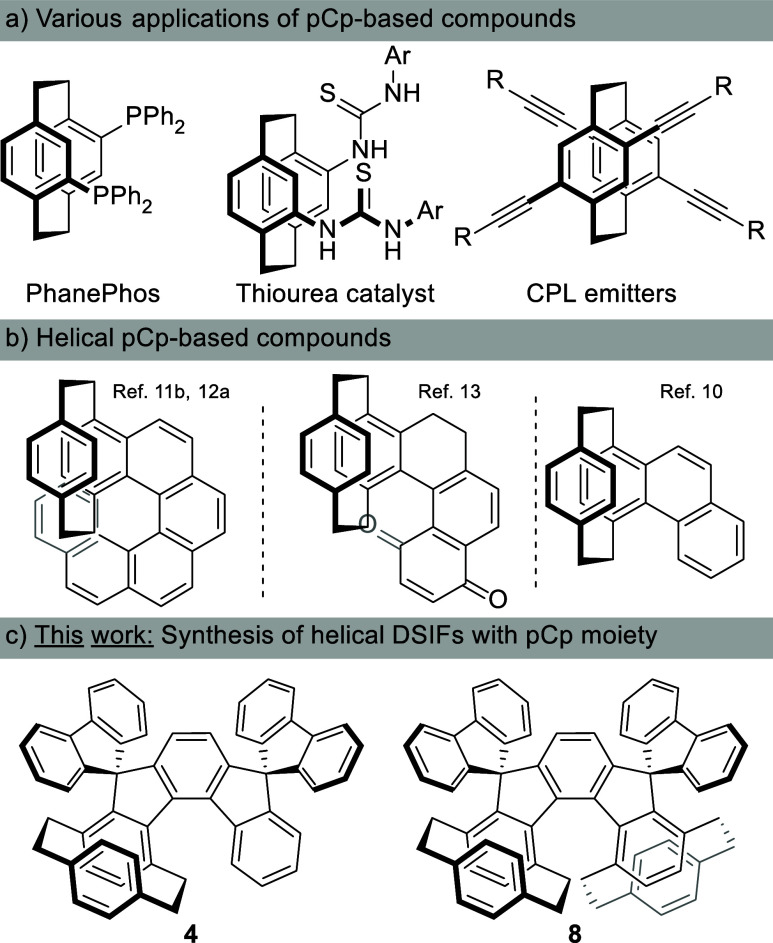
Various systems possess the [2.2]­paracyclophane unit.

Since dispiroindeno­[2,1-*c*]­fluorenes (DSIFs) have been considered as attractive candidates for application in materials science^17^ (the influence of spiro moieties on compounds’ photophysical properties was already discussed by Salbeck and Poriel),^18^ and because of our interest in new synthetic approaches toward chiral aromatic compounds, we previously developed an enantioselective pathway to [7]-helical DSIFs. Indeed, they showed reasonable CPL activity.^19^ However, it remains challenging to prepare such compounds with high enantiopurity using enantioselective cyclotrimerization. Therefore, we decided to explore the possibilities of designing and developing possible synthetic routes based on a different strategy enabling the synthesis of a new type of stereochemical hitherto unprepared hybrid with high optical purity. In this respect, we envisioned that the introduction of (*S*_p_)- and (*R*_p_)-[2.2]­paracyclophane units at the ends of the 5,8-dihydroindeno­[2,1-*c*]­fluorene core will allow it to adopt helical configuration (*P* or *M*) avoiding unfavorable steric interactions. In addition, we expected that such an appropriately substituted scaffold could have a beneficial effect on photophysical properties (high luminescence, etc.).

Herein, we report a synthetic route by using a cyclotrimerization reaction as a pivotal step to access highly enantioenriched [5]-helical mono-pCp- and bis-pCp-indeno­[2,1-*c*]­fluorene derivatives. In addition, we evaluated the photophysical properties of all the newly prepared compounds and compared them with their non-pCp dispiroindeno­[2,1-*c*]­fluorene analogs.

We envisioned that a route to desired helical compounds possessing pCp units at the end of a helical scaffold could be based on the same strategy we used for the synthesis of [5]-, [7]- and [9]-helical [2,1-*c*]­indenofluorenes, i.e., catalytic cyclotrimerization of suitably substituted triynediols.^19,20^ Our initial interest focused on the preparation of (*S*_p_)- and (*R*_p_)-mono-pCp-indeno­[2,1-*c*]­fluorenes **4**. For that purpose, we prepared advanced pCp-containing intermediates: triynediols (*S*_p_)- and (*R*_p_)-**2** in 5 steps by using a combination of the previously reported procedures starting from easily accessible aldehydes (*S*_p_)- and (*R*_p_)-**1** ^21^ (Scheme 1) (for details, see the Supporting Information section). Out of several tested catalytic systems (Table S1), the use of Wilkinson’s catalyst, RhCl­(PPh_3_)_3_, in 1,2-dichloroethane turned out to reproducibly give the desired diketone (*S*_p_)- and (*R*_p_)-**3** in 75 and 81% isolated yields, respectively. The subsequent two-step spirocyclization, utilizing arylation with 2-biphenyllithium followed by acidic treatment, yielded the corresponding dispiro-compounds (*S*_p_,*P*)- and (*R*_p_,*M*)-**4** in 23 and 18% yields, respectively. Structures of **3** and **4** were confirmed by single crystal X-ray analyses (Figures S9 and 2a).

**Scheme 1:**
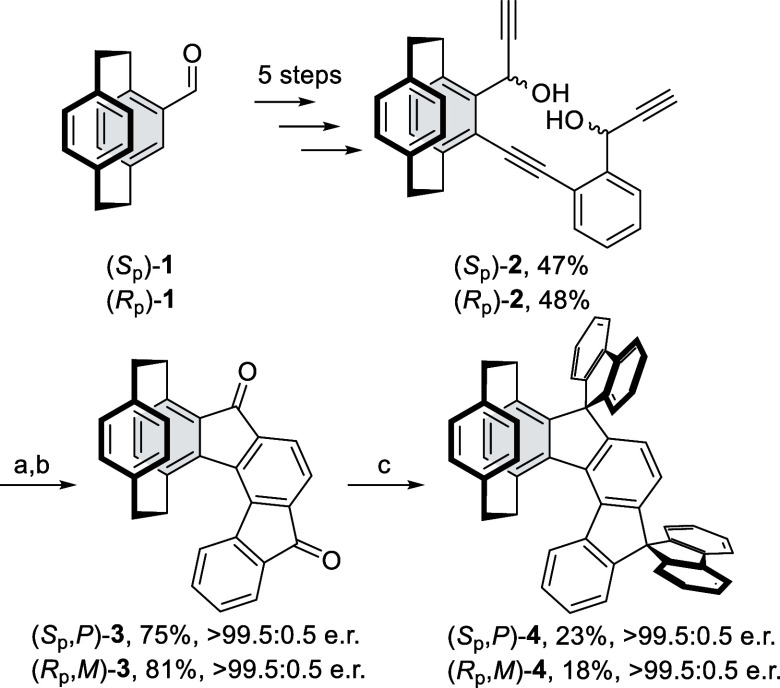
Synthesis of Mono-pCp-DSIF **4** [Fn sch1-fn1]

i a) RhCl­(PPh_3_)_3_ (5 mol %), 1,2-dichloroethane, 100 °C, MW. b) PCC, CH_2_Cl_2_, 25 °C. c) *i.* 2-LiC_6_H_4_Ph, THF, −78 to 25 °C; *ii.* AcOH/HCl, reflux.

With these results in hand, we pursued the synthesis of [5]-helical derivatives bearing pCp units at both ends of the helical scaffold. However, it should be taken into account that cyclotrimerization of a bis-pCp based triyne with (*S*_p_,*S*_p_)- or (*R*_p_,*R*_p_)-configuration will result in the formation of a compound having pCp moieties oriented in opposite directions—*anti* arrangement (Scheme 2, route A), whereas in the case of a triyne possessing (*R*_p_,*S*_p_)- or (*R*_p_,*S*_p_)-configuration, the *meso*-product will be formed having both pCp moieties pointing in the same direction—*syn* arrangement (Scheme 2, route B).

**Scheme 2:**
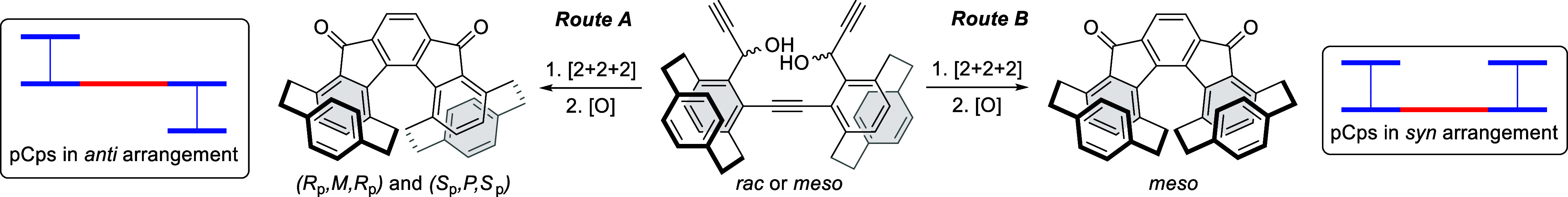
Visualization of Possible Cyclotrimerized Products: [5]-Helical (*R*_p_,*M*,*R*_p_)-Diketone and *meso*-(*R*_p_,*S*_p_)-Diketone

**Scheme 3:**
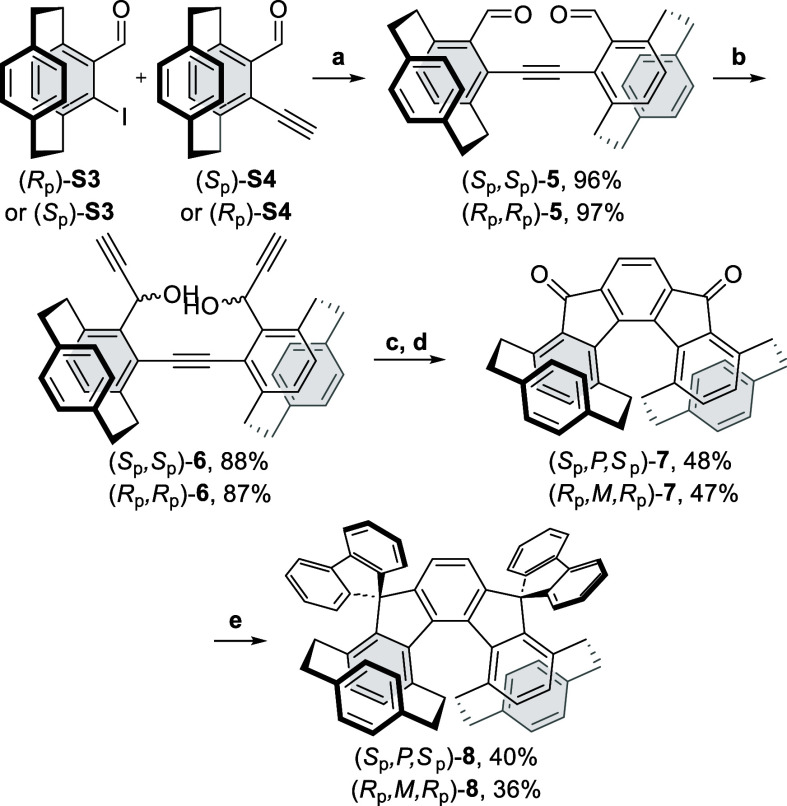
Synthesis of **8** [Fn sch3-fn1]

i a) Pd­(PPh_3_)_2_Cl_2_ (5 mol %), CuI (10 mol %), THF/Et_3_N (2/1), 80 °C. b) ethynylMgBr, THF, 0 to 25 °C. c) RhCl­(PPh_3_)_3_ (5 mol %), 1,2-dichloroethane, 100 °C, MW. d) PCC, CH_2_Cl_2_, 25 °C. e) *i.* 2-LiC_6_H_4_Ph, THF, −78 to 25 °C. *ii.* AcOH/HCl, reflux.

Initially, we focused on the synthesis of (*S*_p_,*P,S*_p_)-**8** (Scheme 3). In the first step, Sonogashira coupling of alkyne (*S*_p_)-**S4** with iodide (*R*_p_)-**S3** provided bis-pCp-substituted dialdehyde (*S*_p_,*S*_p_)-**5** (97%), which, after 2-fold ethynylation with ethynylmagnesium bromide, provided triyne (*S*_p_,*S*_p_)-**6** (87%). Its catalytic cyclotrimerization with Wilkinson’s catalyst gave rise to a mixture of diastereoisomeric [5]-helical indeno­[2,1-*c*]­fluorenediols, which was directly subjected to oxidation with PCC to diketone (*S*_p_,*P,S*_p_)-**7** in 48% yield over 2 steps. It is worth mentioning that reaction conditions were optimized (details in Supporting Information). Surprisingly, full conversion was achieved after only 30 min in a microwave reactor at 100 °C in 1,2-dichloroethane. Afterward, dione (*S*_p_,*P,S*_p_)-**7** was transformed to dispiro compound (*S*_p_,*P,S*_p_)-**8** in 40% yield by using the aforementioned two-step spirocyclization. Finally, the same reaction sequence was carried out with (*R*_p_)-**S4** and (*S*_p_)-**S3** which provided (*R*_p_,*R*_p_)-**5**, (*R*_p_,*R*_p_)-**6**, (*R*_p_,*M,R*_p_)-**7**, and dispiro compound (*R*_p_,*M,R*_p_)-**8** in 97, 87, 47, and 36% yields, respectively. Then, our attention was turned toward the synthesis of *meso-*product. The reaction sequence began with a Sonogashira coupling of iodide (*S*_p_)-**S3** with alkyne (*S*_p_)-**S4,** successfully generating dialdehyde *meso*-**5** (92%). The alkyne was then transformed into triyne *meso*-**6** using the established protocol in 88% yield. Following this, a cyclotrimerization of *meso*-**6** led to the formation of diketone *meso*-**7** (47%) (for detailed synthetic procedures, refer to the Supporting Information). Given the fact that *meso*-diketone **6** is not optically active, it was not converted into a dispiro derivative.

Crystallizing the desired helical compounds enabled us to study their spatial arrangements (Figure 2) and compare them to those of maternal **DSIF** (Figure 3). The sum of the three dihedral angles (φ_3_) for the inner helical rim (C-16–C17–C18-C19–C20-C1), representing the helical twist, of dispiro products **4** and **8** are 41.9° and 47.9°, respectively. They are larger than those for the pristine [5]-helical dispiroindeno­[2,1-*c*]­fluorene (DSIF)^22^ (18.1°) and 6,7-disubstituted indeno­[2,1-*c*]­fluorene (37.5°).^20a^ Values for diketones **3** and **7** were 37.1° and 54.7°, respectively. All sums were lower than the one for the pristine [5]­helicene (63.5°).^23^

**Figure 2:**
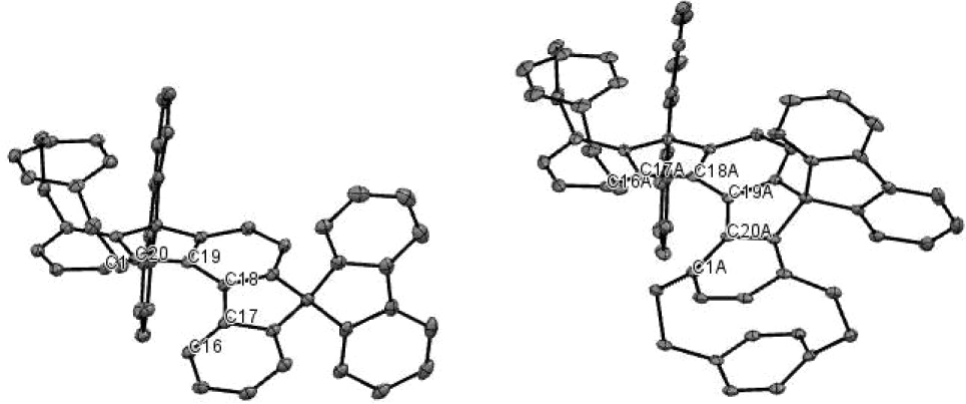
ORTEP drawings of **4** (left) and **8** (right). Ellipsoids are drawn with a 30% probability. Hydrogen atoms were omitted for clarity. Carbon atoms constituting the inner helical rims are marked.

**Figure 3:**
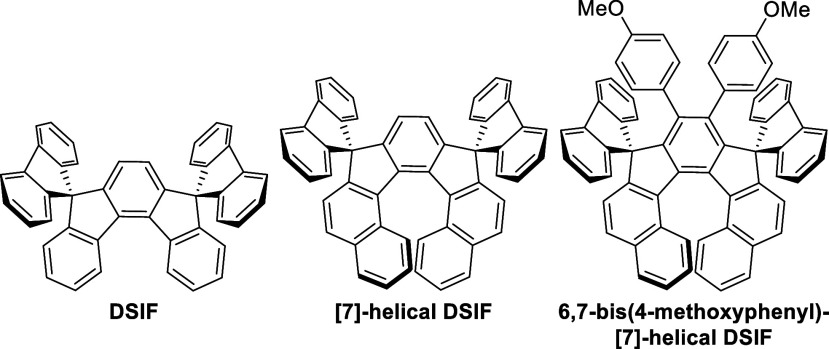
DSIF, [7]-helical DSIF, and 6,7-bis­(4-methoxyphenyl)-[7]-helical DSIF.

The photophysical properties of **4** and **8** were recorded and compared to maternal **DSIF**, developed by Poriel et al., and **[7]-helical DSIF** from our group (Table 1, Figure 3). Originally, photophysical properties of the former were measured by Poriel et al.^22^ for the cyclohexane solution (λ_abs_ = 338, λ_lum_ = 343, 358, Φ_lum_ = not reported) and were remeasured by us for DCM solution (λ_abs_ = 338, λ_lum_ = 346, 360, Φ_lum_ = 87%) for comparison. Interestingly, there was no significant difference in these values, which indicates no evidence of a solvatochromic effect. Compounds **4** and **8** evince high luminescence (Φ_lum_ = 41 and 61%, respectively). Comparison of emission maxima values for maternal **DSIF** (λ_lum_ = 360 nm), **4** (λ_lum_ = 386 nm) and **8** (λ_lum_ = 410 nm) clearly shows that the emission maximum is bathochromically shifted by ∼25 nm for the addition of each pCp moiety. In this respect, the emission maximum for **8** is close to that of [7]-helical DSIF (**[7]-DSIF**)^19^ (λ_lum_ = 423 nm) (Figure S2). The bathochromic shifts from **4** to **8** could be explained by “through-space” conjugation between the aromatic ring of the pCp moiety^24^ and the indeno­[2,1-*c*]­fluorene scaffold. In other words, the presence of the pCp moiety almost has the same effect as that of the naphthalene moiety.

**Table 1:** Photophysical and Chiroptical Properties of **4**, **8**, and Their Structurally Related Compounds

Compd.	λ_abs_ (nm)^a^	λ_lum_ (nm)	Φ_lum_ (%)	|*g*_lum_|
**DSIF**	261, 309, 317, 339	346, 360	87	
**[7]-DSIF**	299, 312, 333, 370, 388	409, 423	87	
**4**	297, 311, 333	386	41	0.3 × 10^–3^
**8**	314, 348, 362	410	61	1.1 × 10^–3^

a Dichloromethane solutions with *c* = 1 × 10^–5^ M.

Regarding the dissymmetry factors |*g*_lum_|, their values for (*S*_p_,*P*)- and (*R*_p_,*M*)-**4** and (*S*_p_,*P,S*_p_)- and (*R*_p_,*M,R*_p_)-**8** were determined to be 3 × 10^–4^ and 1.1 × 10^–3^, respectively (Figures 4, S3, S4, and S7), and they are in the typical range of all-carbon based small molecules bearing the pCp moiety.^7b^ It should be noted that the DFT calculated values for |*g*_lum_| factors of **4** and **8** were estimated to be 4.2 × 10^–4^, and 1.2 × 10^–3^, respectively, which is in good agreement with the experimental values. Simulation of their electronic and magnetic transition dipole moments is shown in Figure S9. Comparison of these values clearly indicates that the presence of two pCp moieties in combination with the helical scaffold has a beneficial effect on chiroptical properties. Moreover, the latter value is close to the one obtained previously for 6,7-bis (4-methoxyphenyl)-[7]-helical DSIF derivative (Figure 3),^19^ which was 1 × 10^–3^. Thanks to the combination of helical and planar chirality, enantiopure compounds **4** and **8** display intense electronic circular dichroism (ECD) responses (see Figures 4, S3 and S5). For instance, (*R*_p_,*M*)-**4** displays a positive band at 267 nm (Δε = +96 M^–1^ cm^–1^), while (*R*_p_,*M,R*_p_)-**8** exhibits a positive band at 295 nm (Δε = +115 M^–1^ cm^–1^). CPL brightness values, *B*_CPL_,^25^ for **4** and **8** were calculated to be ca. 1.1 and 7.6, respectively.

**Figure 4:**
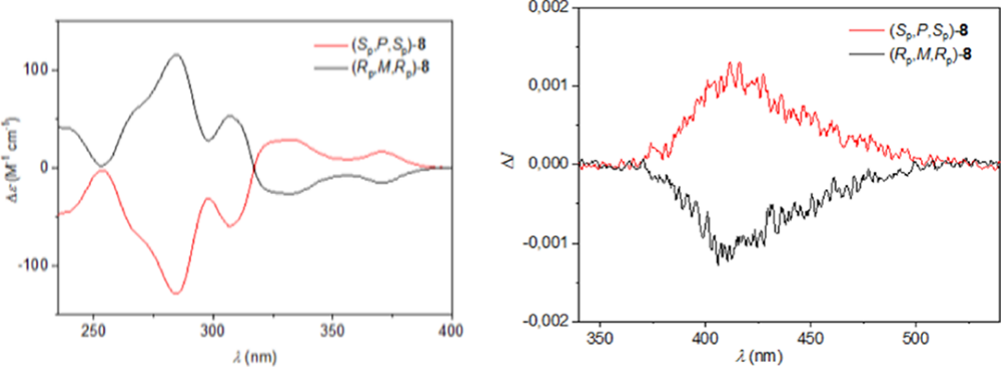
ECD (left) and CPL spectra (right) of **8**.

In summary, we presented a study of the synthesis of hitherto unknown highly enantioenriched dispiroindeno­[2,1-*c*]­fluorenes, possessing one or two pCp units, and their chiroptical properties were described as well. The standard synthetic transformations of the pristine pCp, crowned by catalytic cyclotrimerization of advanced triyne intermediates, allowed us to develop an efficient methodology toward the aforementioned compounds. The final dispiro-compounds have emission maxima in the violet-blue light region at 386 and 410 nm, and are highly luminescent with quantum yields of 41 and 61%. The chiroptical properties underscore the significant impact of molecular structure on the optical activity of these compounds, as illustrated by a luminescence dissymmetry factor of 3 × 10^–4^ and 1.1 × 10^–3^ for **4** and **8**, respectively. *B*_CPL_ values for **4** and **8** were about 1.1 and 7.6, respectively.

## Supplementary Material



## Data Availability

The data underlying this study are available in the published article and in its Supporting Information.

## Notes

Experimental details, analytical data, crystallographic data, NMR spectra, HPLC data, and computational details (PDF)

## References

[1] Brown C. J., Farthing A. C. (1949). Preparation and Structure of Di-*p*-Xylylene. Nature.

[2] b Gleiter, R. ; Hopf, H. , Eds.; Modern Cyclophane Chemistry. Wiley-VCH: Weinheim, Germany, 2004.

[3] Rowlands G. J. (2008). The synthesis of enantiomerically pure [2.2]­paracyclophane
derivatives. Org. Biomol. Chem..

[4] Liang H., Guo W., Li J., Jiang J., Wang J. (2022). Chiral Arene Ligand
as Stereocontroller for Asymmetric C–H Activation. Angew. Chem., Int. Ed..

[5] Felder S., Wu S., Brom J., Micouin L., Benedetti E. (2021). Enantiopure
planar chiral [2.2]­paracyclophanes: Synthesis and applications in
asymmetric organocatalysis. Chirality.

[6] Lopez R., Palomo C. (2022). Planar Chirality: A
Mine for Catalysis
and Structure Discovery. Angew. Chem., Int.
Ed..

[7] Sugiura K.-I. (2020). [2.2]­Paracyclophane-Based Chiral Platforms for Circularly
Polarized Luminescence Fluorophores and Their Chiroptical Properties:
Past and Future. Front. Chem..

[8] Morisaki Y., Gon M., Sasamori T., Tokitoh N., Chujo Y. (2014). Planar Chiral Tetrasubstituted
[2.2]­Paracyclophane: Optical Resolution
and Functionalization. J. Am. Chem. Soc..

[9] Felder S., Delcourt M.-L., Contant D., Rodríguez R., Favereau L., Crassous J., Micouin L., Benedetti E. (2023). Compact CPL
emitters based on a [2.2]­paracyclophane scaffold: Recent developments
and future perspectives. J. Mater. Chem. C.

[10] Hopf H., Mlynek C., El-Tamany S., Ernst L. (1985). [2.2]­(l,4)­Phenanthrenoparacyclophane:
Synthesis and Two-Dimensional Proton and Carbon-13 NMR Study. *J*. Am. Chem. Soc..

[11] Hopf H., Hucker J., Ernst L. (2007). Paracyclophanes.
Part 58 [1]. On the Use of the Stilbene-Phenanthrene Photocyclization
in [2.2] Paracyclophane Chemistry. Polym. J.
Chem..

[12] Nakazaki M., Zahamoto K., Maeda M. (1980). Preparation
of (−)-(*M*)-[2.2]­Paracyclophano-hexahelicene
from (−)-(*M*)-1,4-Dimethylhexahelicene and
Absolute Configuration of 4-Substituted [2.2]­Paracyclophanes. Chem. Lett..

[13] Marrocchi A., Minuti L., Taticchi A., Dix I., Hopf H., Gacs-Baitz E. P. G., Jones P. G. (2001). The Preparation of Helical Cyclophanes
Containing Five-membered Rings. Eur. J. Org.
Chem..

[14] Wang C.-S., Wei J.-C., Chang K.-H., Chou P.-T., Wu Y.-T. (2019). Indeno­[1,2-*b*]­fluorene-Based
2,2]­Cyclophanes with 4*n*/4*n* and 4*n*/4*n*+2]
π Electrons: Syntheses, Structural Analyses, and Excitonic Coupling
Properties. Angew. Chem., Int. Ed..

[15] Wang C.-S., Wei Y.-C., Pan M.-L., Wu C.-H., Chou P.-T., Wu Y.-T. (2021). New [2,2]­Fluorenophanes
Give Insights into Asymmetric Charge Transfer-Mediated
Exciton Delocalization along the π-π Packing Direction. Chem.—Eur. J..

[16] Minuti L., Taticchi A., Marrocchi A., Gacs-Baitz E. (2005). Synthesis
of Helicenophanes Containing Two Carbocyclic Five-Membered Rings. Polycyclic Aromat. Compd..

[17] Fix, A. G. ; Chase, D. T. ; Haley, M. M. Indenofluorenes and Derivatives: Syntheses and Emerging Materials Applications. In Polyarenes I, Siegel, J. ; Wu, Y. T. , Eds.; Springer: Berlin, Heidelberg, 2012; Vol. 349, pp. 159–195. 10.1007/128_2012_376 23097030

[18] Saragi T. P. I., Spehr T., Siebert A., Fuhrmann-Lieker T., Salbeck J. (2007). Spiro Compounds for
Organic Optoelectronics. Chem. Rev..

[19] Cadart T., Nečas D., Kaiser R. P., Favereau L., Císařová I., Gyepes R., Hodačová J., Kalíková K., Bednárová L., Crassous J. (2021). Rhodium
Catalyzed Enantioselective Synthesis of Highly Fluorescent and CPL
Active Dispiroindeno­[2,1-c]­fluorenes. Chem.—Eur.
J..

[20] Kaiser R. P., Nečas D., Cadart T., Gyepes R., Císařová I., Mosinger J., Pospíšil L., Kotora M. (2019). Straightforward
Synthesis and Properties of Highly Fluorescent [5]- and [7]-Helical
Dispiroindeno­[2,1-c]­fluorenes. Angew. Chem.,
Int. Ed..

[21] Kramer J. J. P., Yildiz C., Nieger M., Bräse S. (2014). Direct Access
to 4,5-Disubstituted [2.2]­Paracyclophanes
by Selective ortho-Halogenation with Pd-Catalyzed C–H Activation. Eur. J. Org. Chem..

[22] Romain M., Thiery S., Shirinskaya A., Declairieux C., Tondelier D., Geffroy B., Jeannin O., Rault-Berthelot J., Métivier R., Poriel C. (2015). *ortho*-, *meta*-, and *para*-Dihydroindenofluorene
Derivatives
as Host Materials for Phosphorescent OLEDs *Angew*. Chem., Int. Ed..

[23] Kaehler T., John A., Jin T., Bolte M., Lerner H.-W., Wagner M. (2020). Selective Vicinal Diiodination of Polycyclic Aromatic
Hydrocarbons. Eur. J. Org. Chem..

[24] Felder S., Delcourt M.-L., Bousquet M. H. E., Jacquemin D., Rodríguez R., Favereau L., Crassous J., Micouin L., Benedetti E. (2022). Planar Chiral Analogues of PRODAN
Based on a [2.2]­Paracyclophane Scaffold: Synthesis and Photophysical
Studies. J. Org. Chem..

[25] Arrico L., Di Bari L., Zinna F. (2021). Quantifying the Overall Efficiency
of Circularly Polarized Emitters. Chem.—Eur.
J..

