# Supplementary material for: Helical Dispiroindeno[2,1‑c]fluorenes Possessing Planar Chirality: Synthesis and Chiroptical Properties
Source: J Org Chem. 2025 Dec 11;91(3):1468–72. doi: 10.1021/acs.joc.5c02008 (PMC12836319; doi:10.1021/acs.joc.5c02008)
Supplement: Supplementary file 1 [file jo5c02008_si_001.pdf]

Supporting Infomation

for

Helical Dispiroindeno[2,1-*c*]fluorenes Possessing Planar Chirality: Synthesis and Chiroptical Properties

Marko Bogomolec,[a] Lucia Feriancová,[a] Jhon Sebastian Oviedo Ortiz,[b] Jeanne Crassous,[b] Ivana Císařová,[c] Robert Gyepes,[c] Timothée Cadart,[a]* and Martin Kotora[a]*

[a] Department of Organic Chemistry, Faculty of Science, Charles University, Hlavova 8, 128 00 Praha 2, Czech Republic.

[b] Institut des Sciences Chimiques de Rennes, University of Rennes, CNRS, ISCR, UMR 6226, F-35000 Rennes, France.

[c] Department of Inorganic Chemistry, Faculty of Science, Charles University, Hlavova 8, 128 00 Praha 2, Czech Republic.

Contents

1 Experimental Section 3
2 Synthesis of Starting Building Blocks 4
2.1 Synthesis of pCp-iodo-carbaldehydes S3. 4
2.2 Synthesis of Bisaldehydes S5 and 5 8
2.3 Alkynylation of carbaldehydes 10
3 Synthesis of mono-pCp-dispiro[2,1-*c*]indenofluorenes 13
3.1 Cyclotrimerizations of (*rac*)-2 13
3.2 Cyclotrimerizations of (S_p)-3 and (R_p)-3 15
3.3 Spirocyclization 16
4 Synthesis of bis-pCp-dispiro[2,1-*c*]indenofluorenes 18
4.1 Cyclotrimerizations of (S_p,S_p)-6 18
4.2 Spirocyclization 21
5 Photophysical Properties 23
6 Chiroptical Properties 25
7 X-ray Diffraction Data 28
8 Copies of ^{1}H and ^{13}C{^{1}H} NMR Spectra 34
9 Selected HRMS spectra 54

1 Experimental Section

All commercially available reagents were purchased from Sigma-Aldrich, BLD Pharmatech, Acros Organics, Fluorochem, Alfa Aesar, TCI Chemicals, and Strem Chemicals. Solvents were purified and dried by distillation: tetrahydrofuran (THF) and toluene from sodium/benzophenone, dichloromethane and 1,2-dichloroethane from calcium hydride. Other solvents and reagents were used without further purification. Column chromatography was performed on Silica gel 60A (40-60 μm) from Silicycle. Thin layer chromatography was performed on Silicycle silica gel 60 F_{254} pre-coated aluminum sheets.

NMR spectra were recorded on Bruker AVANCE III Spectrometer (^{1}H at 400 MHz, ^{13}C{^{1}H} at 101 MHz, and ^{19}F at 376 MHz). All NMR spectra were measured as solutions in $CDCl_3$ and referenced to residual solvent signal: $CDCl_3$ (^{1}H, δ_H = 7.26; ^{13}C{^{1}H}, δ_C = 77.16). Coupling constants *J* are given in Hz.

Infrared spectra were recorded with a Nicolet Avatar 370 FTIR spectrophotometer. The method used for measuring was diffuse reflectance (DRIFT) in KBr pellets or Attenuated Total Reflectance (ATR) with a Ge crystal. IR absorptions are given in wavenumbers as cm^{-1}.

MS spectra were recorded on an Agilent Technologies 6530 Accurate-Mass Q-TOF LC/MS spectrometer. Samples were ionized by electrospray ionization (ESI) or atmospheric pressure photoionization (APPI) and detected by quadrupole or TOF. Drying and nebulizer gas was nitrogen.

Melting points are uncorrected and were determined on a Kofler apparatus KB T300.

HPLC analyses were performed with a Shimadzu liquid chromatograph with a spectrophotometric detector (SPD-M20A). Chiral columns Daicel Chiralpak® IB and IC were used for the separation of enantiomers.

Specific optical rotations were measured with AUTOMATIC polarimetry, Autopol III (Rudolph Research, Flandres, New Jersey), and $CHCl_3$ was used as a solvent. Specific optical rotations are given in concentrations *c* [g/100 mL].

The UV/vis absorption spectra were recorded in CH_2Cl_2 solutions (1x10^{-5}) using a Unicam 340 spectrophotometer. Steady-state fluorescence spectra were monitored on an FLS 980 spectrofluorometer (Edinburgh Instruments). Absolute fluorescence quantum yields were determined using a Quantaurus-QY Plus spectrofluorometer equipped with an integrating sphere (Hamamatsu C13534-33). Fluorescence of air-saturated solutions of samples in CH_2Cl_2 was measured in 1 cm path-length cuvettes using samples with an absorbance of 0.1 or less at the excitation wavelength.

Microwave reactions were performed in an Anton Paar Monowave 400 instrument.

Note 1. Systematic names of new products were generated by ChemDraw and don't necessarily follow the IUPAC nomenclature.

Note 2. Isolated yields of products refer to compounds with >97% purity.

2 Synthesis of Starting Building Blocks

Compounds **S2-5** were synthesized according to previously reported procedures (for citations see the respective procedures).

2.1 Synthesis of pCp-iodo-carbaldehydes S3.

Synthesis of (*rac*)-1,4(1,4)-dibenzenacyclohexaphane-1^2-carbaldehyde ((*rac*)-1)

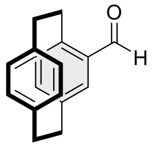

Titanium(IV) chloride (96 mmol, 10.5 mL) was added dropwise to a suspension of [2.2]paracyclophane (48 mmol, 10.0 g) and α,α-dichloromethyl methyl ether (4.5 mL, 50.4 mmol) in CH_2Cl_2 (100 mL) at 0 °C. The reaction mixture was stirred for 16 h at 25 °C. The dark solution was poured onto ice and stirred for 2 hours until complete decolorization. The aqueous phase was extracted with CH_2Cl_2 (3 × 150 mL). Combined organic phases were washed with saturated aq. solution of $NaHCO_3$ (50 mL), dried over Na_2SO_4, filtered and the solvent was evaporated under reduced pressure. Flash column chromatography of the residue on silica gel (6/4 CH_2Cl_2/hexanes) provided 10.75 g (95%) of the title compound as a colorless solid.
R_f (6/4 CH_2Cl_2/hexanes) = 0.26
^{1}H NMR (400 MHz, $CDCl_3$) δ 9.95 (s, 1H), 7.01 (d, *J* = 2.0 Hz, 1H), 6.73 (dd, *J* = 7.8, 2.0 Hz, 1H), 6.62–6.55 (m, 2H), 6.50 (dd, *J* = 7.9, 1.9 Hz, 1H), 6.43 (dd, *J* = 7.8, 1.9 Hz, 1H), 6.37 (dd, *J* = 7.9, 1.9 Hz, 1H), 4.10 (ddd, *J* = 13.1, 9.9, 1.8 Hz, 1H), 3.31–2.83 (m, 7H).
The recorded values agree with the reported data.[1]

Preparation of (S_p)-1 by kinetic resolution[2]

In a flame-dried flask, (*rac*)-4-formyl[2.2]paracyclophane (12.7 mmol, 3.0 g) was dissolved in a mixture of *i*-PrOH/CH_3CN (560 mL, 1/1) under Ar and the solution was cooled to 0 °C. Solution of RuCl(*p*-cymene)[(*S*,*S*)-Ts-DPEN] (0.25 mmol, 159.0 mg) and *t*-BuOK (1.27 mmol, 142.5 mg) in a mixture of *i*-PrOH/CH_3CN (40 mL, 1/1) was then transferred *via* cannula. After stirring for 45 min at 0 °C, the reaction was quenched with saturated aq. solution of NH_4Cl (50 mL). The aqueous phase was extracted with EtOAc (3 × 100 mL), combined organic phases were washed with brine (100 mL), dried over $MgSO_4$ and the solvent was evaporated under reduced pressure. Flash column chromatography of the residue on silica gel (linear gradient: 100/0 to 99/3 CH_2Cl_2/EtOAc) provided 1.18 g (39%) of the title compound (S_p)-**1** as a colorless solid and 1.71 g (56%) of enantioenriched alcohol (S_p)-**1´** as a pale yellow solid.

The same procedure was used for the synthesis of (R_p)-**1** (1.22 g, 41%) and enantioenriched alcohol (R_p)-**1´** (1.71 g, 56%) by using RuCl(*p*-cymene)[(*R*,*R*)-Ts-DPEN].

HPLC conditions: Chiralpak® IC, Heptane/*i*-PrOH 98/2, flow rate 1 mL/min, UV 254 nm.

---

[1] Kramer, J. J. P.; Yildiz, C.; Nieger, M.; Bräse, S. *Eur. J. Org. Chem.* **2014**, 1287–1295.
[2] Delcourt, M.-L.; Turcaud, S.; Benedetti, E.; Micouin, L. *Adv. Synth. Catal.* .**2016**, 358, 1213–1218.

Chart S2. Chromatogram of (*rac*)-**1**

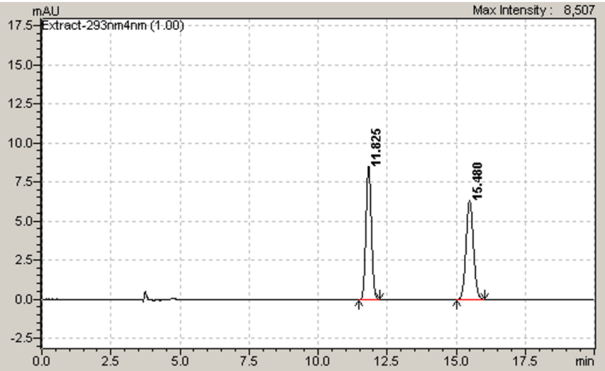

	Retention time (min)	Peak start	Peak end	Area (%)
(R_p)-**1**	11.83	11.48	12.24	50
(S_p)-**1**	15.48	15.02	16.03	50

Chart S3. Chromatogram of (R_p)- and (S_p)-**1**

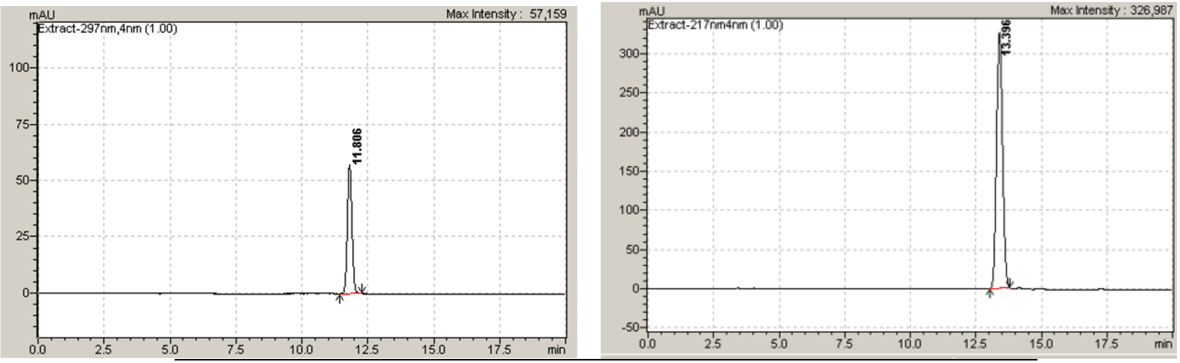

	Retention time (min)	Peak start	Peak end	Area (%)
(R_p)-**1**	11.81	11.45	12.27	>99
(S_p)-**1**	13.39	13.03	13.79	>99

Enantioenriched alcohols (S_p)-**1´** and (R_p)-**1´** were oxidized with MnO_2 to enantioenriched aldehydes (S_p)-**1** and (R_p)-**1**, which were again resolved.

Oxidation of (S_p)-1´

MnO_2 (28 mmol, 2.45 g) was added to a solution of alcohol (S_p)-**1´** (2.34 mmol, 0.56 g) in $CHCl_3$ (40 mL). After stirring for 16 hours at 25 °C, the reaction mixture was filtered through a short pad of silica gel and the solvent were evaporated under reduced pressure affording 555 mg (quantitative) of aldehyde (S_p)-**1** as a white crystalline solid (83:17 e.r.).

Chart S5. Chromatogram of enantioenriched (S_p)-**1** obtained after oxidation of the alcohol (S_p)-**1′**.

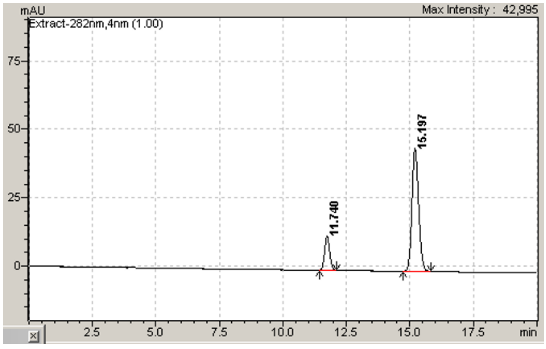

	Retention time (min)	Peak start	Peak end	Area (%)
(R_p)-**1**	11.74	11.46	12.12	17
(S_p)-**1**	15.19	14.74	15.82	83

Synthesis of (*rac*)-1,4(1,4)-dibenzenacyclohexaphane-1²-carbaldehyde O-methyl oxime ((*rac*)-S1)

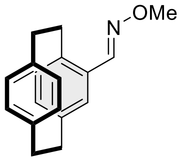

To a solution of (*rac*)-**1** (21 mmol, 5.0 g) in CH_2Cl_2 (50 mL), methoxylamine hydrochloride (25 mmol, 2.1 g) and pyridine (84 mmol, 6.8 mL) were added. The reaction mixture was stirred for 16 hours at 25 °C. Then, volatiles were removed under reduced pressure and flash column chromatography of the residue on silica gel (3/2 CH_2Cl_2/hexanes) of the residue provided 5.23 g (94%) of (*rac*)-**S1** as a white solid.

R_f (3/2 CH_2Cl_2/hexanes) = 0.46

^{1}H NMR (400 MHz, $CDCl_3$) δ 8.09 (s, 1H), 6.78 (d, J = 1.9 Hz, 1H), 6.64–6.59 (m, 1H), 6.59-6.50 (m, 3H), 6.47 (dd, J = 7.5, 3.6 Hz, 2H), 4.03 (d, J = 0.6 Hz, 3H), 3.61 (ddd, J = 13.5, 9.9, 1.8 Hz, 1H), 3.23–2.91 (m, 6H), 2.86 (ddd, J = 13.5, 10.3, 6.7 Hz, 1H).

The recorded values agree with the reported data.[1]

Synthesis of (*R*p)- and (*S*p)-S1.

The same reaction conditions were used for the synthesis of (R_p)- and (S_p)-**S1** starting from (R_p)-**1** (2.54 mmol, 600 mg) and (S_p)-**1** (2.54 mmol, 600 mg), respectively.

(R_p)-**S1**, 654 mg (97%),$[\alpha]_D^{20}$= -315.5 (c 0.42, $CHCl_3$)

(S_p)-**S1**, 645 mg (96%), $[\alpha]_D^{20}$= +345.9 (c 0.43, $CHCl_3$)

Synthesis of (*rac*)-1³-iodo-1,4(1,4)-dibenzenacyclohexaphane-1²-carbaldehyde O-methyl oxime ((*rac*)-S2)

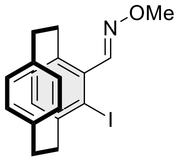

(*rac*)-**S1** (11.0 mmol, 3.0 g), *N*-iodosuccinimide (13.0 mmol, 2.9 g), palladium (II) acetate (2.2 mmol, 494 mg) and silver (I) trifluoroacetate (2.2 mmol, 486 mg) were dissolved in dry 1,2-dichloroethane (50 mL) in a high-pressure tube under Ar atmosphere. The reaction mixture was stirred at 110 °C (oil bath) for 2 hours. The mixture was then filtered over Celite® (CH_2Cl_2 as eluent) and the solvent was evaporated under reduced pressure. Flash column chromatography of the residue on silica gel (1/1 CH_2Cl_2/hexanes) provided 3.49 g (81%) of the title compound (*rac*)-**S2** as a pale yellow solid.

^{1}H NMR (400 MHz, $CDCl_3$) δ 8.05 (s, 1H), 7.01 (dd, J = 7.8, 2.0 Hz, 1H), 6.63–6.60 (m, 2H), 6.58–6.53 (m, 2H), 6.49 (d, J = 7.7 Hz, 1H), 4.03 (s, 3H), 3.79 (ddd, J = 13.0, 9.5, 3.2 Hz, 1H), 3.51–3.43 (m, 1H), 3.32–2.95 (m, 5H), 2.94–2.74 (m, 1H).
The recorded values agree with the reported data.[1]

Synthesis of (R_p)- and (S_p)-S2.

The same reaction conditions were used for the synthesis of (R_p)-S2 (5.09 g, 83%) and (S_p)-**S2** (5.02 g, 82%) starting from (S_p)-**S1** (15.64 mmol, 4.15 g) and (R_p)-**S1** (15.64 mmol, 4.15 g), respectively.

Synthesis of (*rac*)-1³-iodo-1,4(1,4)-dibenzenacyclohexaphane-1²-carbaldehyde ((*rac*)-S3)

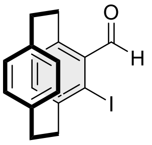

In a MW vial, (*rac*)-**S2** (4.73 mmol, 1.85 g), formaldehyde (aq. 37 %, 47.3 mmol, 3.5 mL), and *p*-toluenesulfonic acid (9.46 mmol, 1.8 g) were dissolved in a mixture of THF/H_2O (11 mL, 10/1). The reaction mixture was stirred for 4 hours under microwave irradiation at 120 °C. After cooling down to room temperature, the reaction mixture was diluted with water (50 mL) and extracted with CH_2Cl_2 (3 × 30 mL). Combined organic phases were dried over Na_2SO_4, filtered and volatiles were removed under reduced pressure. Flash column chromatography of the residue on silica gel (7/3 CH_2Cl_2/hexanes) provided 1.54 g (90%) of the title compound as a colorless solid.
R_f (7/3 CH_2Cl_2/hexanes) = 0.7
^{1}H NMR (300 MHz, $CDCl_3$) δ 9.80 (s, 1H), 7.00 (d, J = 7.8 Hz, 1H), 6.60 (d, J = 2.0 Hz, 3H), 6.50 (d, J = 7.9 Hz, 1H), 6.39 (d, J = 7.9 Hz, 1H), 3.88 (ddd, J = 12.4, 8.2, 4.0 Hz, 1H), 3.64–3.51 (m, 1H), 3.28–3.14 (m, 3H), 3.13–2.94 (m, 2H), 2.82 (ddd, J = 12.7, 9.6, 7.1 Hz, 1H).
The recorded values agree with the reported data.[1]

Synthesis of (R_p)- and (S_p)-S3.

The same procedure was used for the synthesis of (R_p)-**S3** and (S_p)-**S3** starting from (R_p)-**S2** (6.39 mmol, 2.50 g) and (S_p)-**S2** (6.39 mmol, 2.50 g), respectively.
(R_p)-**S3**, 2.11 g (91%), $[\alpha]_D^{20}$ = +152.3 (c 0.44, $CHCl_3$)
(S_p)-**S3**, 2.14 g (92%), $[\alpha]_D^{20}$ = -149.4 (c 0.43, $CHCl_3$)

Synthesis of (*rac*)-1³-ethynyl-1,4(1,4)-dibenzenacyclohexaphane-1²-carbaldehyde ((*rac*)-S4)

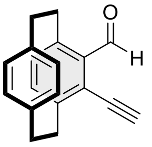

In a flame-dried pressure flask, (*rac*)-**S3** (2.75 mmol, 1.0 g), $Pd(PPh_3)_2Cl_2$ (0.14 mmol, 98 mg) and CuI (0.28 mmol, 53 mg) were dissolved in dry THF (15 mL) under Ar atmosphere. Ethynyltrimethylsilane (3 mmol, 0.43 mL) was then added, followed by Et_3N (7.5 mL). The reaction mixture was stirred at 80 °C (oil bath) for 1 hour. After cooling down to room temperature, the mixture was filtered through a pad of silica and Celite® (CH_2Cl_2 as eluent), and the solvent was evaporated under reduced pressure. K_2CO_3 (8.25 mmol, 1.14 g) was added to a solution of the residue in CH_2Cl_2/MeOH (75 mL, 1/2) and the mixture was stirred at 25 °C for 1 hour. The reaction mixture was diluted with water (50 mL) and extracted with CH_2Cl_2 (3 × 50 mL). Combined organic phases were dried over Na_2SO_4, filtered, and concentrated under reduced pressure.

Flash column chromatography of the residue on silica gel (1/1 hexanes/CH_2Cl_2) provided 596 mg (83%) of the title compound as a light brown solid.
R_f (1/1 hexanes/CH_2Cl_2) = 0.26.
M.p. = 108–109 °C.
^{1}H NMR (400 MHz, $CDCl_3$) δ 10.38 (s, 1H), 6.86 (dd, *J* = 7.9, 2.0 Hz, 1H), 6.69 (d, *J* = 7.9 Hz, 1H), 6.60 (d, *J* = 7.9 Hz, 1H), 6.58 (dd, *J* = 7.9, 2.0 Hz, 1H), 6.49 (dd, *J* = 7.9, 2.0 Hz, 1H), 6.36 (dd, *J* = 7.9, 2.0 Hz, 1H), 4.13 (ddd, *J* = 12.4, 9.8, 2.1 Hz, 1H), 3.72 (s, 1H), 3.66 (ddd, *J* = 13.1, 10.6, 3.3 Hz, 1H), 3.30–3.16 (m, 2H), 3.15–3.10 (m, 1H), 3.09–3.02 (m, 1H), 2.94 (dddd, *J* = 13.1, 10.8, 4.2, 0.6 Hz, 1H), 2.83 (dddd, *J* = 12.7, 10.2, 6.7, 0.6 Hz, 1H).
^{13}C{^{1}H} NMR (101 MHz, $CDCl_3$) δ 193.9, 144.6, 142.9, 140.0, 139.3, 137.7, 137.2, 135.9, 133.6, 132.7, 131.6, 129.7, 128.3, 89.0, 79.1, 34.7, 34.3, 33.9, 33.5.
IR (KBr) ν_{max} 3248, 3064, 3008, 2966, 2891, 2854, 2787, 2767, 2089, 1901, ¸1678, 1550, 1498, 1448, 1232, 1211, 1099, 1018, 991, 874, 798, 681, 582 cm^{-1}.
HRMS (ESI) m/z calcd for $C_{19}H_{17}O$ $[M+H]^+$: 261.1274, found: 261.1276.

Synthesis of (*R*p)- and (*S*p)- S4

The same reaction conditions were used for the synthesis of (R_p)- and (S_p)-**S4** starting from (S_p)-**S3** (5.40 mmol, 1.96 g) and from (R_p)-**S3** (5.52 mmol, 2.00 g), respectively.
(R_p)-**S4**, 1.13 g (81%), $[\alpha]_D^{20}$= +209.8 (*c* 0.49, $CHCl_3$)
(S_p)-**S4**, 1.17 g (82%), $[\alpha]_D^{20}$= -213.5 (*c* 0.66, $CHCl_3$)

2.2 Synthesis of Bisaldehydes S5 and 5

Synthesis of (*rac*)-1^3-((2-formylphenyl)ethynyl)-1,4(1,4)-dibenzenacyclohexaphane-1^2-carbaldehyde ((*rac*)-S5)

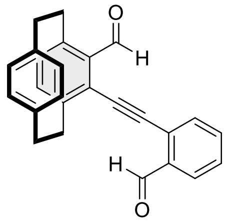

In a flame-dried pressure tube, (*rac*)-**S3** (1.38 mmol, 500 mg), 2-ethynylbenzaldehyde (1.52 mmol, 198 mg), $Pd(PPh_3)_2Cl_2$ (0.07 mmol, 49 mg) and CuI (0.14 mmol, 27 mg) were dissolved in dry THF (10 mL) under Ar atmosphere. Then, Et_3N (5 mL) was added, and the reaction mixture was stirred at 80 °C (oil bath) for 1 hour. After cooling down to room temperature, the mixture was filtered through a pad of silica and Celite® (DCM as eluent) and volatiles were removed under reduced pressure. Flash column chromatography of the residue on silica gel (7/3 CH_2Cl_2/hexanes) provided 357 mg (71%) of the title compounds as a yellow amorphous solid.
R_f (7/3 CH_2Cl_2/hexanes) = 0.35.
Mp = 149–150 °C.
^{1}H NMR (400 MHz, $CDCl_3$) δ 10.65 (s, 1H), 10.49 (s, 1H), 8.01 (dt, *J* = 8.0, 0.9 Hz, 1H), 7.76 (dd, *J* = 7.7, 1.2 Hz, 1H), 7.67 (td, *J* = 7.5, 1.4 Hz, 1H), 7.55 (t, *J* = 7.5 Hz, 1H), 6.84 (dd, *J* = 7.8, 2.0 Hz, 1H), 6.76 (d, *J* = 7.9 Hz, 1H), 6.66 (d, *J* = 7.8 Hz, 1H), 6.62 (dd, *J* = 7.9, 2.0 Hz, 1H), 6.53 (dd, *J* = 7.9, 2.0 Hz, 1H), 6.41 (dd, *J* = 7.9, 2.0 Hz, 1H), 4.16 (ddd, *J* = 12.5, 10.0, 2.0 Hz, 1H), 3.74 (ddd, *J* = 13.4, 10.6, 3.1 Hz, 1H), 3.34–2.98 (m, 5H), 2.88 (ddd, *J* = 12.8, 10.2, 6.5 Hz, 1H).
^{13}C{^{1}H} NMR (101 MHz, $CDCl_3$) δ 193.0, 190.9, 144.4, 143.3, 139.9, 139.1, 137.9, 136.7, 136.1, 135.9, 133.9, 133.7, 133.6, 132.7, 131.6, 129.9, 129.2, 128.5, 128.3, 125.9, 96.5, 91.7, 34.6, 34.3, 34.2, 33.8.

IR (KBr) ν_{max} 2954, 2927, 2195, 1699, 1684, 1591, 1192, 798, 758 cm^{-1}.
HRMS (ESI): *m/z* calcd for $C_{26}H_{20}NaO_2$ [$(M+Na)^+$]: 387.1355, found: 387.1351.

Synthesis of (*R*p)- and (*S*p)-S5.
The same reaction conditions were used for the synthesis of (*S*p)- and (*R*p)-**S5** starting from (*R*p)-**S3** (1.5 g, 4.14 mmol) and (*S*p)-**S3** (1.5 g, 4.14 mmol).
(*S*p)-**S5**, 344 mg (65%), $[\alpha]_D^{20}$= +156.3 (*c* 0.48, $CHCl_3$)
(*R*p)-**S5**, 350 mg (66%), $[\alpha]_D^{20}$= –167.7 (*c* 0.48, $CHCl_3$)

Synthesis of (*rac*)-Bis(1,4(1,4)-dibenzenacyclohexaphane-1²-carbaldehyde)-1³-yl))ethyne ((*rac*)-5)

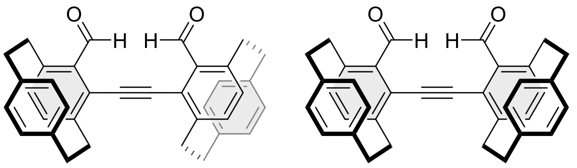

rac *meso*

In a flame-dried pressure tube, (*rac*)-**S3** (2.76 mmol, 1.00 g), alkyne (*rac*)-**S4** (3.04 mmol, 791 mg), $Pd(PPh_3)_2Cl_2$ (0.14 mmol, 98 mg) and CuI (0.28 mmol, 53 mg) were dissolved in dry THF (15 mL) under Ar atmosphere. Then, Et_3N (7.5 mL) was added, and the reaction mixture was stirred at 80 °C (oil bath) for 1 hour. After cooling down to room temperature, the mixture was filtered through a pad of silica and Celite® ($CHCl_3$ as eluent) and volatiles were removed under reduced pressure. Flash column chromatography of the residue on silica gel (7/3 CH_2Cl_2/hexanes) provided 1.35 g (98%) of a diastereomeric mixture of the title compounds as a yellow amorphous solid.

Synthesis of (*S*p,*S*p)- and (*R*p,*R*p)-5.

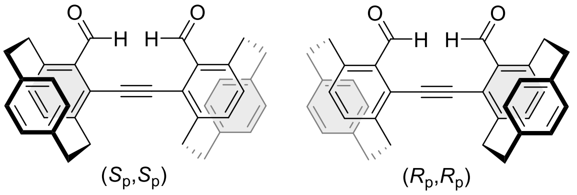

(*S*p,*S*p) (*R*p,*R*p)

The same procedure was used synthesis of (*R*p,*R*p)-**5** from iodide (*S*p)-**S3** (215 mg, 0.82 mmol) and alkyne (*R*p)-**S4** (272 mg, 0.75 mmol), (*S*p,*S*p)-**5** from iodide (*R*p)-**S3** (215 mg, 0.82 mmol) and alkyne (*S*p)-**S4** (272 mg, 0.75 mmol).
(*R*p,*R*p)-**5**, 363 mg (97%), $[\alpha]_D^{20}$= +318 (*c* 0.47, $CHCl_3$),
(*S*p,*S*p)-**5**, 337 mg (96%), $[\alpha]_D^{20}$= -284 (*c* 0.60, $CHCl_3$)

(*S*p,*S*p)-Bis(1,4(1,4)-dibenzenacyclohexaphane-1²-carbaldehyde)-1³-yl))ethyne ((*S*p,*S*p)-5).
R*f* (7/3 CH_2Cl_2/hexanes) = 0.35.
M.p. = decomposition > 200 °C.
^{1}H NMR (400 MHz, $CDCl_3$) δ 10.53 (s, 2H), 6.92 (dd, *J* = 7.9, 2.0 Hz, 2H), 6.79 (d, *J* = 7.9 Hz, 2H), 6.67 (d, *J* = 7.9 Hz, 2H), 6.66 (dd, *J* = 7.9, 2.0 Hz, 2H), 6.57 (dd, *J* = 7.9, 2.0 Hz, 2H), 6.48 (dd, *J* = 7.9, 2.0 Hz, 2H), 4.16 (ddd, *J* = 12.4, 9.8, 2.1 Hz, 2H), 3.83–3.75 (m, 2H), 3.40–3.31 (m, 2H), 3.30–3.07 (m, 8H), 2.91 (ddd, *J* = 12.9, 10.2, 6.6 Hz, 2H).
$^{13}C\{^1H\}$ NMR (101 MHz, $CDCl_3$) δ 193.0, 144.2, 143.5, 140.3, 139.1, 138.0, 136.9, 136.1, 133.7, 132.9, 131.7, 130.1, 128.7, 96.1, 34.8, 34.52, 34.46, 34.0.
IR (KBr) ν_{max} 3064, 3005, 2995, 2935, 2887, 2846, 2779, 2746, 1900, 1763, 1685, 1595, 1564, 1500, 1466, 1394, 1321, 1219, 941, 874, 798, 717, 661, 588, 515 cm^{-1}.
HRMS (ESI) m/z calcd for $C_{36}H_{31}O_2$ $[M+H]^+$: 495.2319, found: 495.2319.

Bis((*R*$_p$,*S*$_p$)-(1,4(1,4)-dibenzenacyclohexaphane-1^2-carbaldehyde)-1^3-yl))ethyne (*meso*-5)

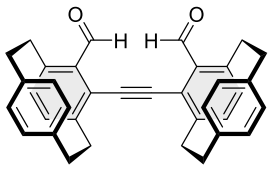

The title compound was prepared according to the same procedure from (S_p)-**6** (634 mg, 1.75 mmol) and (S_p)-**S4** (502 mg, 1.93 mmol), flash column chromatography provided 796 mg (92%) of the title compound.
R_f (7/3 CH_2Cl_2/hexanes) = 0.50.
M.p. = decomposition.
^{1}H NMR (400 MHz, $CDCl_3$) δ 10.57 (s, 2H), 6.84 (dd, J = 7.9, 2.0 Hz, 2H), 6.79 (d, J = 7.9 Hz, 2H), 6.67 (d, J = 7.8 Hz, 2H), 6.65 (dd, J = 7.9, 2.0 Hz, 2H), 6.55 (dd, J = 7.9, 2.0 Hz, 2H), 6.45 (dd, J = 7.9, 2.0 Hz, 2H), 4.17 (ddd, J = 12.5, 10.0, 2.0 Hz, 2H), 3.74–3.67 (m, 2H), 3.33–3.03 (m, 10H), 2.91 (ddd, J = 12.9, 10.2, 6.6 Hz, 2H).
^{13}C{^{1}H} NMR (101 MHz, $CDCl_3$) δ 193.1, 144.1, 143.5, 140.2, 139.1, 138.0, 136.8, 136.1, 133.7, 132.8, 131.7, 130.1, 128.7, 96.2, 34.8, 34.6, 34.5, 34.0.
IR (KBr) ν_{max} 3064, 3005, 2995, 2935, 2887, 2846, 2779, 2746, 1900, 1763, 1685, 1595, 1564, 1500, 1466, 1394, 1321, 1219, 941, 874, 798, 717, 661, 588, 515 cm^{-1}.
HRMS (ESI) m/z calcd for $C_{36}H_{31}O_2$ $[M+H]^+$: 495.2319, found: 495.2319.

2.3 Alkynylation of carbaldehydes

(*rac*)-1-(2-((1^3-(1-Hydroxyprop-2-yn-1-yl)-1,4(1,4)-dibenzenacyclohexaphane-1^2-yl)ethynyl)phenyl)prop-2-yn-1-ol ((*rac*)-2)

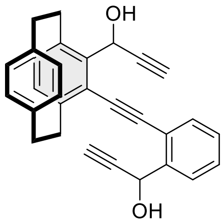

In a flame-dried Schlenk flask, bisaldehyde (*rac*)-**S5** (2.13 mmol, 775 mg) was dissolved in dry THF (35 mL), and the suspension was cooled to 0 °C under Ar atmosphere. Ethynylmagnesium bromide (0.5 M in THF, 8.52 mmol, 17.04 mL) was added dropwise. Afterwards, the reaction mixture was allowed to warm up and stirred at 25 °C for additional 2 hours. The reaction mixture was quenched with saturated aq. solution of NH_4Cl (50 mL), diluted with water (50 mL), and extracted with EtOAc (3 × 30 mL). The combined organic phases were washed with brine (50 mL), dried over Na_2SO_4, filtered, and the solvent was evaporated under reduced pressure. Flash column chromatography of the residue on silica gel (19/1 CH_2Cl_2/EtOAc) provided 831 mg (94%) of a diastereomeric mixture of the title compound as an amorphous light brown solid.
R_f (19/1 CH_2Cl_2/EtOAc) = 0.44 and 0.28.
M.p. = 160–162 °C.
Due to a mixture of four diastereoisomers, it was not possible to assign the peaks to individual stereoisomers. For ^{1}H spectrum characteristic peaks are given and for ^{13}C all peaks are listed.
^{1}H NMR (400 MHz, $CDCl_3$) δ 7.85–7.76 (m, 1H), 7.70–7.57 (m, 1H), 7.48–7.32 (m, 1H), 6.92–6.72 (m, 2H), 6.69–6.59 (m, 2H), 6.53–6.46 (m, 2H), 6.16–5.73 (m, 2H), 3.74–3.50 (m, 2H), 3.34–3.14 (m, 2H), 3.13–2.94 (m, 4H), 2.77–2.66 (m, 2H).
^{13}C{^{1}H} NMR (101 MHz, $CDCl_3$) δ 144.4, 144.0, 143.42, 143.36, 143.2, 141.2, 141.14, 141.11, 141.0, 140.0, 139.9, 139.6, 139.49, 139.46, 139.42, 139.2, 139.12, 139.07, 138.6, 138.48, 138.45, 138.40, 135.9, 135.7, 135.60, 135.50, 134.31, 134.26, 134.22, 134.17, 133.8, 133.2, 133.0, 132.88, 132.84, 132.79, 132.75, 132.71, 132.60, 132.57, 132.55, 132.49, 132.45, 132.37, 132.30, 130.39, 130.37, 130.31, 130.26, 130.1, 129.3, 129.2, 129.1, 129.0, 128.83, 128.80, 128.77, 128.70, 127.2, 127.03, 126.98, 126.94, 126.88, 122.9, 122.8, 122.5, 122.3, 122.2,

122.03, 121.97, 97.1, 96.5, 95.7, 92.4, 92.3, 92.2, 84.2, 84.1, 83.09, 83.05, 82.9, 80.1, 77.4, 75.64, 75.56, 75.51, 75.19, 75.16, 74.8, 74.22, 74.18, 63.33, 63.29, 63.27, 62.9, 62.8, 61.8, 61.4, 61.2, 61.0, 35.64, 35.62, 35.25, 35.21, 34.63, 34.61, 34.02, 33.99, 33.8, 33.4, 33.3, 33.14, 33.12.
IR (KBr) ν_{max} 3535 (br.), 3280, 2931, 2854, 2200, 2114, 1483, 1269, 1020, 760, 667 cm^{-1}.
HRMS (ESI) *m/z* calcd for $C_{30}H_{24}NaO_2$ $[(M+Na)^+]$: 439.1669, found: 439.1665.

Synthesis of (*S*p)- and **(*R*p)-2**.
The same reaction conditions were used for the synthesis of (*Rp*)- and (*Sp*)-**2** starting from (*R*p)-**S5** (900 mg, 2.47 mmol) and (*S*p)-**S5** (900 mg, 2.47 mmol).
(*R*p)-**2**, 920 mg (89%),
(*S*p)-**2**, 915 mg (89%).

(*S*p,*S*p)-Bis((1-(1,4(1,4)-dibenzenacyclohexaphane-1²-yl)prop-2-yn-1-ol)-1³-yl)ethyne ((*S*p,*S*p)-6)

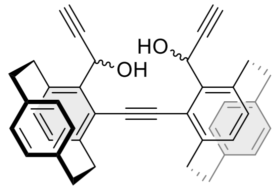

In a flame-dried Schlenk flask, bisaldehyde (*S*p,*S*p)-**5** (0.67 mmol, 330 mg) was suspended in dry THF (15 mL), and the suspension was cooled to 0 °C under Ar atmosphere. Ethynylmagnesium bromide (0.5 M in THF, 2.68 mmol, 5.36 mL) was added dropwise. Afterwards, the reaction mixture was allowed to warm up and stirred at 25 °C for additional 2 hours. The reaction mixture was quenched with saturated aq. solution of NH_4Cl (10 mL), diluted with water (50 mL), and extracted with EtOAc (3 × 30 mL). The combined organic phases were washed with brine (50 mL), dried over $MgSO_4$, filtered, and solvent was evaporated under reduced pressure. Flash column chromatography of the residue on silica gel (97/3 CH_2Cl_2/EtOAc) provided 323 mg (88%) of a diastereomeric mixture of the title compound as an amorphous light brown solid.
R_f (97/3 CH_2Cl_2/EtOAc) = 0.39 and 0.29.
M.p. = 105–110 °C.
Major diastereoisomer – ^{1}H NMR (400 MHz, $CDCl_3$) δ 6.89 (d, *J* = 7.9 Hz, 2H), 6.81 (d, *J* = 7.9 Hz, 2H), 6.72–6.65 (m, 4H), 6.56–6.48 (m, 4H), 6.03 (d, *J* = 2.4 Hz, 1H), 5.91 (d, *J* = 2.4 Hz, 1H), 3.83–3.61 (m, 4H), 3.37–3.23 (m, 4H), 3.15–3.00 (m, 8H), 2.83 (d, *J* = 2.4 Hz, 1H), 2.67 (d, *J* = 2.4 Hz, 1H).
$^{13}C\{^1H\}$ NMR (101 MHz, $CDCl_3$) δ 143.9, 143.2, 140.5, 139.7, 139.5, 139.41, 139.39, 138.7, 138.2, 136.0, 135.9, 134.42, 134.37, 133.1, 132.7, 132.6, 132.5 (2C), 132.4, 130.7, 130.4, 123.3, 122.4, 96.0, 94.8, 84.6, 84.5, 75.7, 74.1, 62.2, 61.4, 35.8, 35.2, 34.8, 34.7, 34.1 (2C), 33.8, 33.6, 33.3.
Minor diastereoisomer – ^{1}H NMR (400 MHz, $CDCl_3$) δ 6.97 (dd, *J* = 7.9, 1.7 Hz, 2H), 6.08 (d, *J* = 2.5 Hz, 2H), 6.72–6.65 (m, 4H), 6.56–6.48 (m, 4H), 6.08 (d, *J* = 2.5 Hz, 2H), 3.83–3.61 (m, 4H), 3.37–3.23 (m, 4H), 3.15–3.00 (m, 8H), 2.73 (d, *J* = 2.4 Hz, 2H).
$^{13}C\{^1H\}$ NMR (101 MHz, $CDCl_3$) δ 143.3, 140.4, 139.5, 139.2, 138.2, 135.4, 134.3, 132.8, 132.6, 132.3, 130.4, 123.6, 95.6, 84.4, 74.2, 61.1, 35.7, 34.6, 34.1, 33.4.
IR (KBr) ν_{max} 3518, 3282, 3010, 2927, 2893, 2582, 2243, 2191, 2112, 1898, 1695, 1595, 1574, 1500, 1464, 1452, 1412, 1319, 1250, 1099, 1018, 955, 910, 864, 796, 733, 663, 519 cm^{-1}.
HRMS (ESI) m/z calcd for $C_{40}H_{34}NaO_2$ $[M+Na]^+$: 569.2451, found: 569.2449.

(R_p,R_p)-Bis((1-(1,4(1,4)-dibenzenacyclohexaphane-1²-yl)prop-2-yn-1-ol)-1³-yl)ethyne ((R_p,R_p)-6)

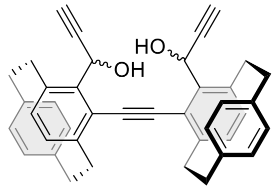

The title compound was prepared according to the same procedure from (R_p,R_p)-bisaldehyde (0.67 mmol, 330 mg), flash column chromatography provided 310 mg (87%) of the title compound.

Bis((R_p,S_p)-1-(1,4(1,4)-dibenzenacyclohexaphane-1²-yl)prop-2-yn-1-ol)-1³-yl)ethyne

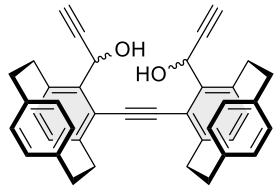

The title compound was prepared according to the same procedure from *meso*-bisaldehyde **5** (750 mg, 1.52 mmol), flash column chromatography provided 730 mg (88%) of the title compound.

R_f (97/3 CH_2Cl_2/EtOAc) = 0.42, 0.29.

M.p. = 118–122 °C.

Major isomer – ^{1}H NMR (400 MHz, $CDCl_3$) δ 6.99–6.88 (m, 4H), 6.73–6.69 (m, 4H), 6.56–6.51 (m, 4H), 6.15 (d, J = 2.5 Hz, 1H), 5.90 (d, J = 2.5 Hz, 1H), 3.88–3.59 (m, 4H), 3.45–3.22 (m, 4H), 3.22–2.97 (m, 8H), 2.78 (d, J = 2.4 Hz, 1H), 2.61 (d, J = 2.5 Hz, 1H).

^{13}C{^{1}H} NMR (101 MHz, $CDCl_3$) δ 144.1, 142.7, 141.1, 139.9, 139.4, 139.3, 139.2, 138.3, 137.9, 136.0, 135.9, 134.5, 134.3, 133.6, 133.1, 132.8, 132.6, 132.4, 132.3, 130.9, 130.7, 130.2, 123.8, 122.2, 97.0, 94.4, 84.8, 84.1, 75.4, 73.6, 62.0, 61.3, 35.8, 35.24, 35.22, 35.17, 34.3, 33.8, 33.7, 33.4.

Minor isomer – ^{1}H NMR (400 MHz, $CDCl_3$) δ 6.88–6.81 (m, 4H), 6.68–6.65 (m, 4H), 6.51–6.48 (m, 4H), 5.98 (d, J = 2.5 Hz, 2H), 3.88–3.59 (m, 4H), 3.45–3.22 (m, 4H), 3.22–2.97 (m, 8H), 2.72 (d, J = 2.5 Hz, 2H).

^{13}C{^{1}H} NMR (101 MHz, $CDCl_3$) δ 143.1, 140.2, 139.6, 139.4, 139.3, 138.2, 135.5, 134.3, 133.0, 132.3, 130.9, 123.5, 95.0, 84.5, 74.3, 61.3, 35.7, 35.1, 34.1, 33.5.

IR (KBr) ν_{max} 3518, 3282, 3010, 2927, 2893, 2582, 2243, 2191, 2112, 1898, 1695, 1595, 1574, 1500, 1464, 1452, 1412, 1319, 1250, 1099, 1018, 955, 910, 864, 796, 733, 663, 519 cm^{-1}.

HRMS (ESI) m/z calcd for $C_{40}H_{34}NaO_2$ $[M+Na]^+$: 569.2451, found: 569.2449.

3 Synthesis of mono-pCp-dispiro[2,1-*c*]indenofluorenes

3.1 Cyclotrimerizations of (*rac*)-2

Table S1. Cyclotrimerization of (*rac*)-**2** using different catalytic systems.

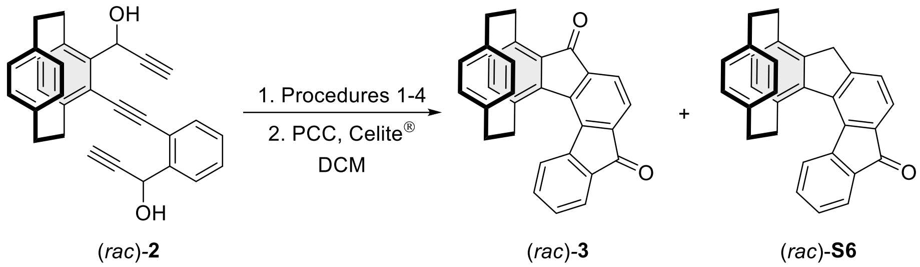

Entry	Catalyst	Ligand	Solvent	T (°C)	**3** (%)	**S6** (%)
1	$RhCl(PPh_3)_3$	-	DCE	90	63	0
2	$RhCl(PPh_3)_3$	-	PhMe	110	65	6
3	Ni(cod)DQ[a]	PPh_3	PhMe	110	19	-
4	$Rh(cod)_2BF_4$	dppb	DCE	90	41	12
5	Cp*Ru(cod)Cl	-	DCE	90	21	-
6[b]	$RhCl(PPh_3)_3$	-	DCE	100	83	-

[a] DQ = duroquinone, cod = 1,5-cycloctadiene. [b] = Under microwave irradiation, reaction time 30 min instead of 16 h.

Procedure 1: $RhCl(PPh_3)_3$ catalyzed [2+2+2] cyclotrimerization in DCE followed by oxidation reaction.

In a flame-dried MW vial, (*rac*)-**2** (0.48 mmol, 200 mg) and $RhCl(PPh_3)_3$ (0.02 mmol, 19 mg) were dissolved in dry 1,2-dichloroethane (8 mL) under Ar atmosphere. The reaction mixture was stirred at 100 °C under microwave irradiation for 30 min. After cooling down to room temperature, the mixture was concentrated under reduced pressure. Celite® (414 mg) and pyridinium chlorochromate (1.92 mmol, 414 mg) were added to a solution of crude bis-pCp-IF-diols in CH_2Cl_2 (15 mL). After stirring for 1 hour at 25 °C, the reaction mixture was concentrated under reduced pressure. Purification by flash column chromatography on silica gel (CH_2Cl_2) yielded 165 mg (83%) of (*rac*)-**3** as an orange crystalline solid.

Procedure 2: Ni(cod)(DQ) catalyzed [2+2+2] cyclotrimerization followed by oxidation reaction.

A dry microwave vial was charged with Ni(cod)(DQ) (0.0121 mmol, 3.9 mg) and PPh_3 (0.024 mmol, 6.3 mg) in dry toluene (3 mL). After 15 min of stirring, the triynediol (*rac*)-**2** (0.12 mmol, 50 mg) was added to the solution. Afterwards, the vial was sealed and heated at 110 °C for 16 h. Then the reaction mixture was cooled down to 20 °C, filtered through a short pad of Celite® and the solvent was evaporated under reduced pressure. Pyridinium chlorochromate (0.48 mmol, 104 mg) and Celite® (104 mg) were added to a solution of the crude diol in CH_2Cl_2 (5 mL) and the mixture was stirred at 25 °C for 3 hours. Afterwards, the reaction mixture was filtered through a pad of 1:4 silica gel/Celite® (Et_2O eluent), and volatiles were removed under reduced pressure. Flash column chromatography of the residue on silica gel (5/1 hexanes/EtOAc) provided 9.6 mg (19%) of compound (*rac*)-**3** as orange solid.

Procedure 3: $Rh(cod)_2BF_4$ catalyzed [2+2+2] cyclotrimerization followed by oxidation reaction.

A dry microwave vial was charged with $Rh(cod)_2BF_4$ (0.012 mmol, 4.9 mg) and dppb (0.014 mmol, 6.1 mg) in dry DCE (3 mL) under argon atmosphere. H_2 gas was bubbled into the solution for 30 min.[3] Afterwards, triynediol (*rac*)-**2** (0.12 mmol, 50 mg) was added under argon atmosphere. The reaction was stirred at 90 °C for 16 hours. Then, the reaction mixture was concentrated under reduced pressure. Pyridinium chlorochromate (0.48 mmol, 104 mg) and Celite® (104 mg) were added to a solution of the crude diol in CH_2Cl_2 (5 mL) and stirred at 25 °C for 3 hours. Afterwards, the reaction mixture was filtered through a pad of 1:4 silica gel/Celite® (Et_2O eluent), and volatiles were removed under reduced pressure. Flash column chromatography of the residue on silica gel (5/1 hexanes/EtOAc) provided 20 mg (40%) of compound (*rac*)-**3** as orange solid and 5.1 mg (11%) of (*rac*)-**S6** as a light-yellow solid.

Procedure 4: Cp*Ru(cod)Cl catalyzed [2+2+2] cyclotrimerization followed by oxidation reaction.

A dry microwave vial was charged with Cp*Ru(cod)Cl (0.003 mmol, 1.1 mg) and triynediol (*rac*)-**2** (0.06 mmol, 25 mg) in a dry DCE (1.5 mL) under argon atmosphere. The reaction was stirred at 90 °C for 16 hours. Then, the reaction mixture was concentrated under reduced pressure. Pyridinium chlorochromate (0.24 mmol, 52 mg) and Celite® (52 mg) were added to a solution of the crude diol in CH_2Cl_2 (5 mL) and stirred at 25 °C for 2 hours. Afterwards, the reaction mixture was filtered through a pad of 1:4 silica gel/Celite® (Et_2O eluent), and volatiles were removed under reduced pressure. Flash column chromatography of the residue on silica gel (5/1 hexanes/EtOAc) provided 5.3 mg (21%) of compound (*rac*)-**3** as an orange solid.

(*rac*)-1^5,1^8-Dihydro-1(1,4)-indeno[2,1-c]fluorena-4(1,4)-benzenacyclohexaphane-1^5,1^8-dione ((*rac*)-3)

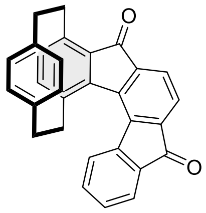

R_f (CH_2Cl_2) = 0.44.
M.p. = 258–259 °C.
^1H NMR (400 MHz, $CDCl_3$) δ 7.79 (dt, J = 7.4, 1.0 Hz, 1H), 7.71 (s, 2H), 7.58–7.47 (m, 2H), 7.39 (ddd, J = 7.3, 6.2, 2.3 Hz, 1H), 6.79 (dd, J = 7.8, 1.9 Hz, 1H), 6.67 (dd, J = 7.3, 1.3 Hz, 2H), 6.51 (d, J = 8.0 Hz, 1H), 6.39 (dd, J = 7.9, 1.9 Hz, 1H), 6.19 (dd, J = 8.1, 1.9 Hz, 1H), 4.25–4.16 (m, 1H), 3.25–3.18 (m, 3H), 3.15–2.98 (m, 2H), 2.89–2.27 (m, 2H).
$^{13}C\{^1H\}$ NMR (101 MHz, $CDCl_3$) δ 193.3, 192.7, 145.0, 144.4, 141.7, 141.5, 140.7, 140.34, 140.30, 139.4, 139.3, 137.8, 137.5, 137.3, 136.2, 134.9, 134.6, 133.9, 132.1, 129.7 (2C), 129.4, 124.63, 124.61, 124.5, 124.3, 36.1, 35.5, 33.3, 32.4.
HRMS (APPI): m/z calcd for $C_{30}H_{20}O_2$ [M^+]: 412.1458, found: 412.1457.

[3] Thiel, I.; Horstmann, M.; Jungk, P.; Keller, S.; Fisher, F.; Drexler, H.-J.; Heller, D.; Hapke, M. *Chem. Eur. J.* **2017**, *23*, 17048–17057.

(*rac*)-1^5,1^8-Dihydro-1(1,4)-indeno[2,1-c]fluorena-4(1,4)-benzenacyclohexaphan-1^8-one ((*rac*)-S6)

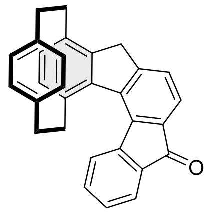

R_f (5/1 hexanes/EtOAc) = 0.53.
M.p. = 240–241 °C.
^{1}H NMR (400 MHz, $CDCl_3$) δ 7.75–7.62 (m, 3H), 7.50–7.43 (m, 2H), 7.30 (td, J = 7.4, 1.0 Hz, 1H), 6.76–6.66 (m, 3H), 6.56 (d, J = 7.8 Hz, 1H), 5.81 (d, J = 8.2 Hz, 1H), 3.78 (d, J = 22.6 Hz, 1H), 3.69 (d, J = 22.6 Hz, 1H), 3.33–3.08 (m, 5H), 3.01–2.71 (m, 3H).
$^{13}C\{^1H\}$ NMR (101 MHz, $CDCl_3$) δ 194.2, 152.5, 145.7, 145.5, 141.6, 139.6, 139.1, 138.3, 137.8, 136.3, 135.2, 134.7, 134.3, 134.0, 133.5, 132.0, 129.7, 129.4, 128.8, 128.7, 126.5, 124.4, 124.2, 123.9, 122.2, 37.5, 36.4, 35.3, 33.8, 32.9.
HRMS (APPI): *m/z* calcd for $C_{30}H_{22}O$ [M^+]: 398.1665, found: 398.1663.

Note: Compound **S6** is formed during oxidation of the cyclized triynediol. However, a plausible reaction mechanism explaining its formation is not clear at the moment.

3.2 Cyclotrimerizations of (S_p)-3 and (R_p)-3

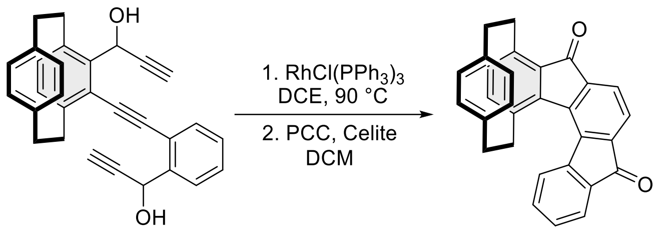

The same reaction conditions as those in **Procedure 1** were used for the synthesis of (R_p)- and (S_p)-**3**, starting from (R_p)-**2** (200 mg, 0.48 mmol) and (S_p)-**2** (200 mg, 0.48 mmol), respectively.
(S_p)-**3**, 149 mg (75%), >99% ee, $[\alpha]_D^{20}$= +940 (c 0.10, $CHCl_3$),
(R_p)-**3**, 161 mg (81%), >99% ee, $[\alpha]_D^{20}$= -894 (c 0.10, $CHCl_3$).

HPLC conditions: Chiralpak® IB, Heptane/*i*-PrOH 95/5, flow rate 1 mL/min, UV 254 nm.

Chart S6. Chromatogram of (*rac*)-**3**.

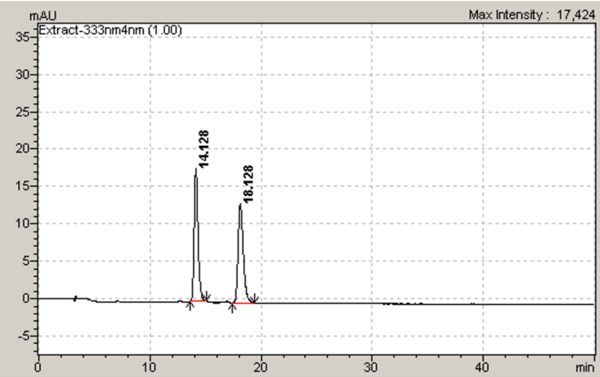

Retention time	Peak start	Peak end	Area(%)
14.13	13.61	15.08	50
18.13	17.41	19.41	50

Chart S7. Chromatogram of (S_p)-**3** (left chart) and (R_p)-**3** (right chart)

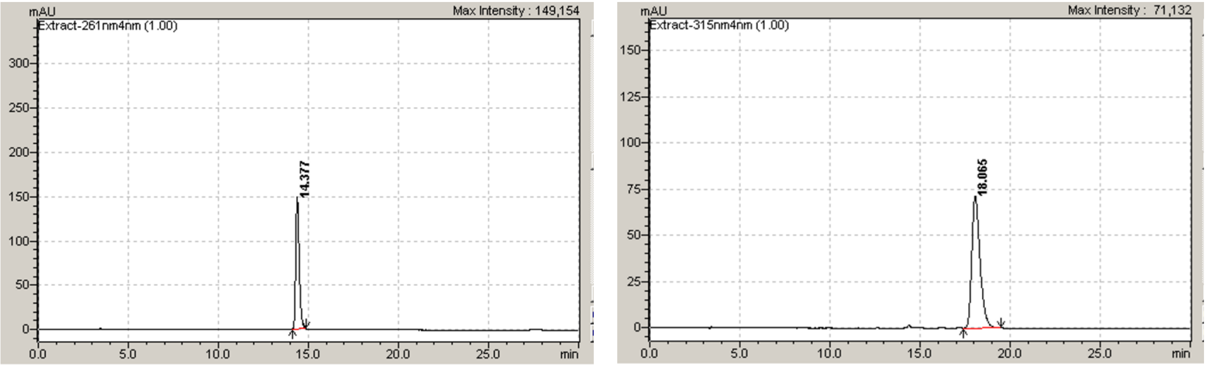

	Retention time	Peak start	Peak end	Area (%)
(S_p)-**3**	14.38	14.12	14.88	>99
(R_p)-**3**	18.07	17.41	19.49	>99

3.3 Spirocyclization

Synthesis of (*rac*)-dispiro[fluorene-9,5'-1(1,4)-indeno[2,1-c]fluorena-4(1,4)-benzenacyclohexaphane-8',9''-fluorene] ((*rac*)-4)

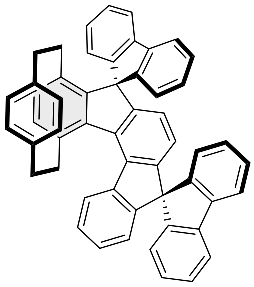

n-BuLi (1.6 M in hexanes, 0.73 mmol, 0.45 mL) was added dropwise to a solution of 2-bromobiphenyl (0.73 mmol, 0.13 mL) in dry THF (2 mL) at -15 °C. The resulting mixture was stirred for 1 hour at the same temperature, followed by the dropwise addition through cannula of (*rac*)-**3** (0.12 mmol, 50 mg) in dry THF (4 mL) and stirred for 30 min at -15 °C, it was allowed to reach 25 °C and stirred overnight. Then, the reaction mixture was poured into the saturated aq. solution of NH_4Cl and extracted with CH_2Cl_2 (3 × 5 mL). The combined organic layers were dried over Na_2SO_4, filtered, and concentrated under reduced pressure. The residue was purified by column chromatography on silica gel (5/1 hexanes/EtOAc). Fractions containing mixture of alcohols were combined, concentrated under reduced pressure. A suspension of the residue in CH_3COOH (3 mL) and HCl (0.2 mL) was refluxed for 2 hours. The resulting mixture was neutralized with the saturated aq. solution of K_2CO_3, extracted with CH_2Cl_2 (3 × 10 mL), dried over Na_2SO_4, filtered, and concentrated under reduced pressure. Flash column chromatography of the residue on silica gel (toluene) afforded 46 mg (55%) of the title compound as a white solid.

R_f (3/1 hexanes/toluene) = 0.20.

M.p. = 301–302 °C.

^{1}H NMR (400 MHz, $CDCl_3$) δ 8.16 (d, J = 7.8 Hz, 1H), 7.90 (d, J = 7.6 Hz, 1H), 7.86–7.80 (m, 2H), 7.78–7.73 (m, 1H), 7.58 (d, J = 7.6 Hz, 1H), 7.51 (td, J = 7.5, 1.1 Hz, 1H), 7.43–7.30 (m, 4H), 7.23–7.03 (m, 8H), 6.94 (td, J = 7.5, 1.2 Hz, 1H), 6.85 (dd, J = 7.7, 1.9 Hz, 1H), 6.77 (d, J = 8.0 Hz, 2H), 6.68–6.61 (m, 2H), 6.56 (d, J = 7.7 Hz, 1H), 6.40–6.24 (m, 4H), 6.16 (d, J = 7.7 Hz, 1H), 3.78–3.68 (m, 1H), 3.53–3.41 (m, 1H), 3.38–3.26 (m, 1H), 3.10–2.99 (m, 1H), 2.68–2.60 (m, 1H), 2.40–2.38 (m, 1H), 2.32–2.24 (m, 1H).

^{13}C{^{1}H} NMR (101 MHz, $CDCl_3$) δ 151.3, 149.8, 149.6, 149.5, 148.8, 147.6, 145.9, 143.5, 142.7, 142.3, 141.5, 140.4, 140.3, 140.0, 139.9, 136.4, 136.1, 135.9, 134.3, 134.0, 132.6, 131.9,

131.4, 130.1, 128.4, 128.0, 127.92, 127.90, 127.85, 127.73, 127.70, 127.5, 127.2, 126.9, 126.6, 124.6, 124.0, 123.82, 123.79, 123.7, 121.95, 121.92, 120.38, 120.35, 119.89, 119.87, 66.4, 66.2, 37.3, 35.8, 34.4, 30.9.

HRMS (APPI): *m/z* calcd for $C_{54}H_{36}$ [M^+]: 684.2812, found: 684.2798.

Synthesis of (S_p,*P*)- and (R_p,*M*)-4.

The same reaction conditions were used for the synthesis of (R_p,*M*)- and (S_p,*P*)-**4**, starting from (R_p,*M*)-**3** (80 mg, 0.194 mmol) and (S_p,*P*)-**3** (80 mg, 0.194 mmol), respectively.

(S_p,*P*)-**4**, 30 mg, 23%, >99% ee, $[\alpha]_D^{20}$= +66.7 (*c* 0.66, $CHCl_3$),

(R_p,*M*)-**4**, 24 mg, 18%, >99% ee, $[\alpha]_D^{20}$= -60.6 (*c* 0.66, $CHCl_3$).

<u>HPLC conditions:</u> Chiralpak® IB, Heptane/*i*-PrOH 99/1, flow rate 1 mL/min, UV 254 nm.

Chart S8. Chromatogram of (*rac*)-**4.**

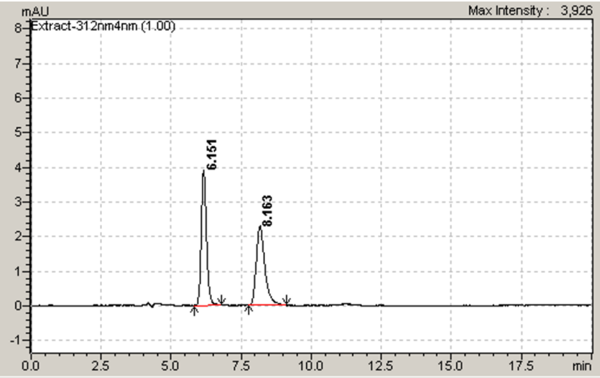

	Retention time	Peak start	Peak end	Area (%)
(S_p,*P*)-**4**	6.15	5.82	6.79	50.8
(R_p,*M*)-**4**	8.16	7.77	9.12	49.1

Chart S9. Chromatogram of (S_p,*P*)-**4** (left chart) and (R_p,*M*)-**4** (right chart)

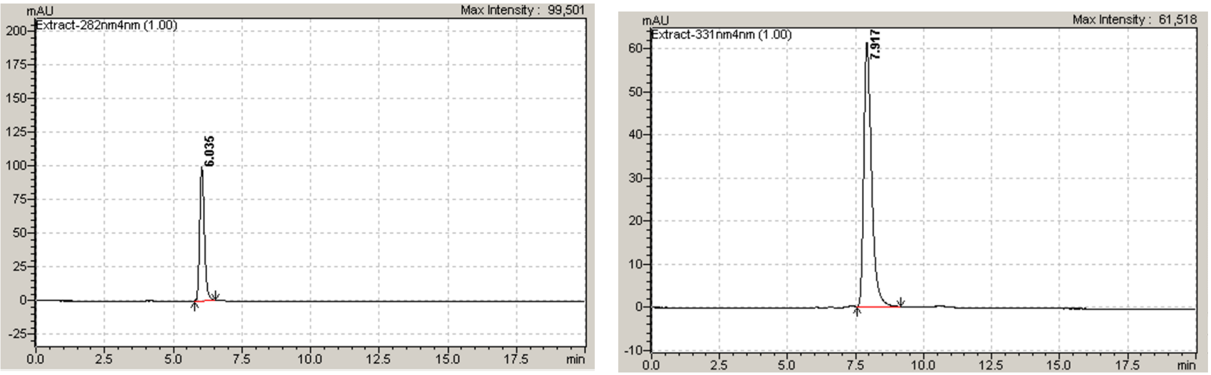

	Retention time	Peak start	Peak end	Area (%)
(S_p,*P*)-**4**	6.03	5.78	6.54	>99
(R_p,*M*)-**4**	7.92	7.55	9.16	>99

4 Synthesis of bis-pCp-dispiro[2,1-*c*]indenofluorenes

4.1 Cyclotrimerizations of (*S*p,*S*p)-6

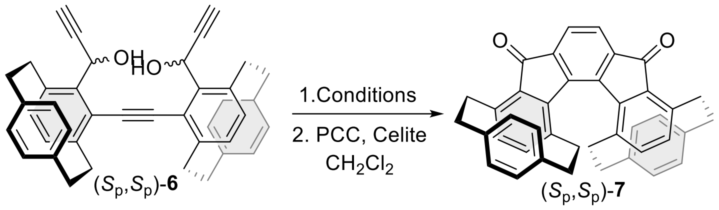

Table S2. Cyclotrimerization of enantiopure (*S*p,*S*p)-**6** using different catalytic systems.

Entry[a]	Catalyst	Ligand (10 mol%)	Solvent	Yield (%)[b]
1	CpCo(dmfu)[P(OEt)$_3$] (10 mol%)	-	THF	43
2	Ni(cod)DQ (10 mol%)	dppf	THF	38
3	Ni(cod)DQ (10 mol%)	dppm	THF	40
4	RhCl(PPh$_3$)$_3$ (10 mol%)	-	THF	44
5	RhCl(PPh$_3$)$_3$ (10 mol%)	-	DCE	52
6	RhCl(PPh$_3$)$_3$ (10 mol%)	-	toluene	51
7	RhCl(PPh$_3$)$_3$ (5 mol%)	-	DCE	48
8[c]	RhCl(PPh$_3$)$_3$ (5 mol%)	-	DCE	48

[a] 0.2 mmol scale, 100 ℃, 1 h, microwave irradiation.

[b] Isolated yields after oxidation (over 2 steps).

[c] Reaction time 30 mins.

(*S*p,*P*,*S*p)-2,3,8,9,16,17,22,23-octahydro-1,10:15,24-di(epiethane[1,2]diylidene)-4,7:18,21-diethenodicyclododeca[*a*,*i*]-*as*-indacene-11,14-dione ((*S*p,*P*,*S*p)-7).

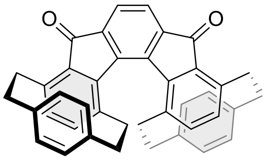

In a flame-dried MW vial, (*S*p,*P*,*S*p)-**6** (0.20 mmol, 109 mg) and RhCl(PPh$_3$)$_3$ (0.01 mmol, 9.3 mg) were dissolved in dry 1,2-dichloroethane (4 mL) under Ar atmosphere. The reaction mixture was stirred at 100 °C under microwave irradiation for 30 min. After cooling down to room temperature, the mixture was concentrated under reduced pressure. Celite® (172 mg) and pyridinium chlorochromate (0.80 mmol, 172 mg) were added to a solution of crude bis-pCp-IF-diols in CH_2Cl_2 (10 mL). After stirring for 1 hour at 25 °C, the reaction mixture was concentrated under reduced pressure. Flash column chromatography of the residue on silica gel (CH_2Cl_2) yielded 51 mg (47%) of the title compound as an orange crystalline solid.

$[\alpha]_D^{20}$= +670.9 (*c* 0.43, $CHCl_3$).

R_f (CH_2Cl_2) = 0.22.

M.p. = 186–190 °C.

$[\alpha]_D^{20}$= +670.9 (*c* 0.429, $CHCl_3$).

^{1}H NMR (400 MHz, $CDCl_3$) δ 7.82 (s, 2H), 6.70 (dd, *J* = 7.8, 1.9 Hz, 2H), 6.65 (d, *J* = 8.0 Hz, 2H), 6.57 (d, *J* = 8.0 Hz, 2H), 6.55 (dd, *J* = 7.8, 1.9 Hz, 2H), 6.34 (dd, *J* = 7.9, 2.0 Hz, 2H), 5.93 (dd, *J* = 7.9, 2.0 Hz, 2H), 4.31 (ddd, *J* = 12.8, 8.7, 4.1 Hz, 2H), 3.26–3.14 (m, 4H), 3.04–2.94 (m, 2H), 2.88–2.69 (m, 6H), 2.55–2.44 (m, 2H).

$^{13}C\{^{1}H\}$ APT NMR (101 MHz, $CDCl_3$) δ 193.8 (C), 146.3 (C), 141.8 (C), 141.4 (C), 140.5 (CH), 140.0 (C), 139.8 (C), 139.4 (C), 138.8 (C), 137.1 (CH), 135.8 (C), 133.5 (CH), 132.7 (CH), 129.28 (CH), 129.27 (CH), 124.1 (CH), 35.6 (CH_2), 34.5 (CH_2), 33.4 (CH_2), 31.8 (CH_2).
IR (ATR) ν_{max} 3064, 3026, 3005, 2922, 2848, 1691, 1599, 1549, 1250, 1232, 1113, 1030, 933, 795, 658, 515 cm^{-1}.
HRMS (ESI) m/z calcd for $C_{40}H_{31}O_2$ $[M+H]^+$: 543.2319, found: 543.2322.
HPLC conditions: Chiralpak® IB, heptane/*i*-PrOH 95/5, 1 mL.min^{-1}, 30 min, UV 254 nm.

(R_p,*M*,R_p)-2,3,8,9,16,17,22,23-octahydro-1,10:15,24-di(epiethane[1,2]diylidene)-4,7:18,21-diethenodicyclododeca[*a*,*i*]-*as*-indacene-11,14-dione ((R_p,*M*,R_p)-7).

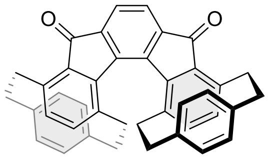

In a flame-dried MW vial, (R_p,*M*,R_p)-**6** (0.2 mmol, 109 mg) and $RhCl(PPh_3)_3$ (0.01 mmol, 9.3 mg) was dissolved in dry DCE (4 mL) under Ar atmosphere. The reaction mixture was stirred at 100 °C under microwave irradiation for 30 min. After cooling down to room temperature, the solvent was evaporated under reduced pressure. Celite® (172 mg) and pyridinium chlorochromate (0.8 mmol, 172 mg) were added to the solution of crude diols in CH_2Cl_2 (15 mL). After stirring for 1 h at 25 °C, the reaction was concentrated under reduced pressure. Flash column chromatography of the residue on silica gel (CH_2Cl_2) provided 52 mg (48%) of the title compound as an orange crystalline solid.
$[\alpha]_D^{20}$= -685.5 (*c* 0.38, $CHCl_3$).

Chart S10. Chromatogram of (*rac*)-**7**

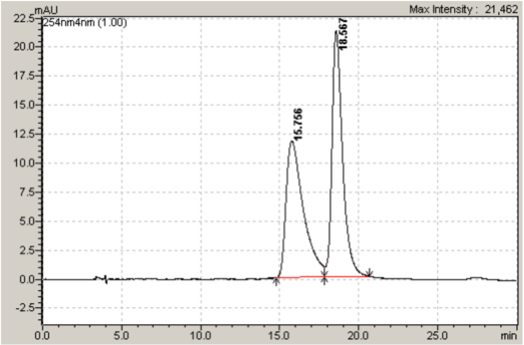

	Retention time	Peak start	Peak end	Area%
1	15.756	14.805	17.845	48
2	18.567	17.845	20.683	52

Chart S11. Chromatogram of (S_p,P,S_p)-**7** (left chart) and (R_p,M,R_p)-**7** (right chart)

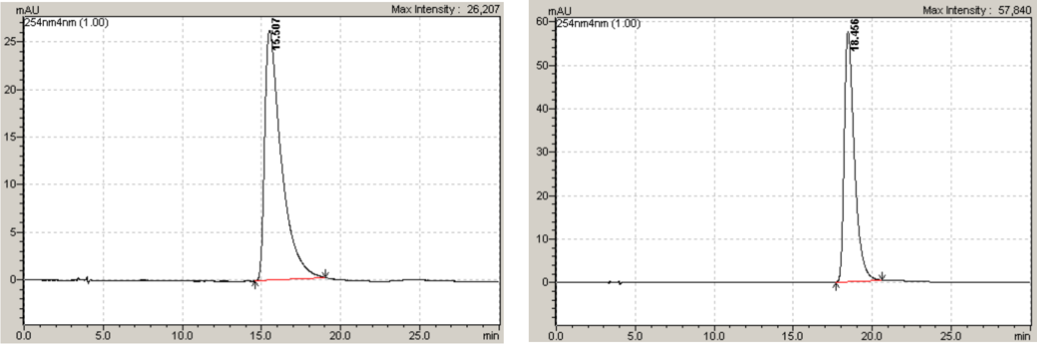

	Retention time	Peak start	Peak end	Area%
(S_p,P,S_p)-**7**	15.507	14.624	19.051	>99
(R_p,M,R_p)-**7**	18.456	17.675	20.619	>99

(R_p,S_p)-2,3,8,9,16,17,22,23-octahydro-1,10:15,24-di(epiethane[1,2]diylidene)-4,7:18,21-diethenodicyclododeca[*a*,*i*]-*as*-indacene-11,14-dione (*meso*-7)

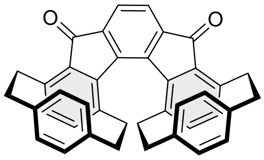

In a flame-dried MW vial, *meso*-**6** (0.46 mmol, 250 mg) and $RhCl(PPh_3)_3$ (23 µmol, 9.3 mg) were dissolved in dry 1,2-dichloroethane (4 mL) under Ar atmosphere. The reaction mixture was stirred at 100 °C under microwave irradiation for 30 min. After cooling down to room temperature, the mixture was concentrated under reduced pressure. Celite® (172 mg) and pyridinium chlorochromate (0.80 mmol, 172 mg) were added to a solution of crude bis-pCp-IF-diols in CH_2Cl_2 (10 mL). After stirring for 1 hour at 25 °C, the reaction mixture was concentrated under reduced pressure. Flash column chromatography of the residue on silica gel (CH_2Cl_2) yielded 51 mg (47%) of the title compound as an orange crystalline solid.

R_f (CH_2Cl_2) = 0.22.

M.p. = 241–245 °C.

^{1}H NMR (400 MHz, $CDCl_3$) δ 7.56 (s, 2H), 6.83 (d, J = 7.8 Hz, 2H), 6.74 (d, J = 7.8 Hz, 2H), 6.57 (d, J = 8.0 Hz, 2H), 6.50 (d, J = 8.0 Hz, 4H), 6.33 (d, J = 7.8 Hz, 2H), 4.29–4.19 (m, 2H), 4.06 (ddd, J = 14.5, 10.6, 5.3 Hz, 2H), 3.44–3.17 (m, 8H), 3.05 (ddd, J = 15.3, 11.1, 5.2 Hz, 2H), 2.84 (td, J = 11.5, 5.4 Hz, 2H).

^{13}C{^{1}H} APT NMR (101 MHz, $CDCl_3$) δ 193.2 (C), 146.3 (C), 144.1 (C), 141.8 (C), 141.0 (CH), 140.6 (C), 140.5 (C), 138.9 (C), 138.4 (C), 136.9 (CH), 134.9 (C), 133.5 (CH), 132.1 (CH), 130.9 (CH), 123.1 (2 CH), 36.1 (CH_2), 35.2 (CH_2), 34.1 (CH_2), 31.4 (CH_2).

IR (ATR) ν_{max} 3064, 3026, 3005, 2922, 2848, 1691, 1599, 1549, 1250, 1232, 1113, 1030, 933, 795, 658, 515 cm^{-1}.

HRMS (ESI) *m/z* calcd for $C_{40}H_{31}O_2$ [M+H]$^+$: 543.2319, found: 543.2322.

4.2 Spirocyclization

Synthesis of (S_p,P,S_p)-2',3',8',9',16',17',22',23'-octahydrodispiro[fluorene-9,11'-[1,10:15,24]di(epiethane[1,2]diylidene)[4,7:18,21]diethenodicyclododeca[*a*,*i*]-*as*-indacene-14',9''-fluorene] ((S_p,P,S_p)-8)

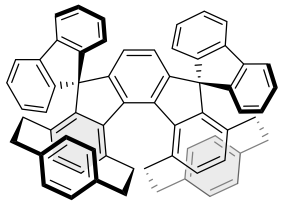

In a flame-dried Schlenk flask, *n*-BuLi (1.6 M in hexanes, 6.08 mmol, 1.16 mL) was added dropwise to a solution of 2-iodobiphenyl (1.08 mmol, 0.19 mL) in dry THF (5 mL) at -78 °C under Ar atmosphere in a Schlenk flask. After stirring for 1 hour, solution of (S_p,P,S_p)-**7** (0.18 mmol, 100 mg) in THF (10 mL) was added dropwise, allowed to warm up and the mixture was stirred for 2 hours at 25 °C. The reaction mixture was quenched with saturated aq. solution of NH_4Cl (10 mL), diluted with water (10 mL), and extracted with CH_2Cl_2 (3 × 20 mL). Combined organic phases were dried over Na_2SO_4, filtered, and concentrated under reduced pressure. A suspension of crude diols in acetic acid (10 mL) and conc. aq. solution of HCl (0.1 mL) was heated under reflux for 2 hours. After cooling down to room temperature, the mixture was diluted with water (30 mL), neutralized with saturated aq. solution of K_2CO_3, and extracted with $CHCl_3$ (3 × 20 mL). Combined organic phases were dried over Na_2SO_4, filtered, and the solvent was evaporated under reduced pressure. Flash column chromatography of the residue on silica gel (1/1 hexanes/CH_2Cl_2), followed by trituration with hexane to give 59 mg (40%) of the title compound as a white amorphous solid.

$[\alpha]_D^{20}$= -11.6 (*c* 0.478, $CHCl_3$)

R_f (1/1 hexane/CH_2Cl_2) = 0.50.

M.p. = 247–248 °C.

^{1}H NMR (400 MHz, $CDCl_3$) δ 7.92 (d, J = 7.3 Hz, 2H), 7.79 (d, J = 7.5 Hz, 2H), 7.64 (d, J = 7.5 Hz, 2H), 7.55 (td, J = 7.5, 0.9 Hz, 2H), 7.38 (td, J = 7.5, 1.1 Hz, 2H), 7.27 (td, J = 7.5, 1.0 Hz, 2H), 7.07 (td, J = 7.5, 1.0 Hz, 2H), 6.82–6.70 (m, 4H), 6.62 (dd, J = 8.0, 1.3 Hz, 2H), 6.57 (d, J = 7.6 Hz, 2H), 6.46 (d, J = 7.9 Hz, 2H), 6.37 (s, 2H), 6.13 (d, J = 7.7 Hz, 2H), 6.06 (dd, J = 8.1, 1.4 Hz, 2H), 3.35–3.26 (m, 2H), 3.22–3.04 (m, 4H), 2.95–2.85 (m, 2H), 2.69–2.60 (m, 2H), 2.46–2.25 (m, 6H).

^{13}C{^{1}H} NMR (101 MHz, $CDCl_3$) δ 151.6 (C), 150.0 (C), 147.7 (C), 146.3 (C), 143.7 (C), 141.7 (C), 140.2 (C), 139.8 (C), 139.6 (C), 136.7 (C), 136.1 (CH), 136.0 (C), 135.1 (CH), 132.6 (CH), 132.5 (C), 131.9 (CH), 131.4 (CH), 129.9 (CH), 128.5 (CH), 128.0 (CH), 127.8 (CH), 127.1 (CH), 126.9 (CH), 123.2 (CH), 120.3 (CH), 120.26 (CH), 120.20 (CH), 66.9 (C), 36.6 (CH_2), 35.7 (CH_2), 34.5 (CH_2), 31.2 (CH_2).

IR (ATR) ν_{max} 3041, 3010, 2968, 2920, 2852, 1599, 1576, 1500, 1473, 1444, 1410, 1365, 1302, 1174, 1161, 1097, 1024, 926, 870, 808, 739, 638, 517 cm^{-1}.

HRMS (APPI) *m/z* calcd for $C_{64}H_{47}$ [M+H]$^+$: 815.3672, found: 815.3664.

<u>HPLC conditions:</u> Chiralpak® IB, heptane/*i*-PrOH 95/5, 1 mL.min^{-1}, 30 min, UV 254 nm.

(R_p,M,R_p)-2',3',8',9',16',17',22',23'-octahydrodispiro[fluorene-9,11'-[1,10:15,24]di(epiethane[1,2]diylidene)[4,7:18,21]diethenodicyclododeca[*a*,*i*]-*as*-indacene-14',9''-fluorene] ((R_p,M,R_p)-8)

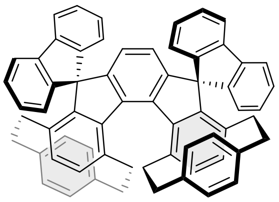

The same procedure as above was used for synthesis of (R_p,M,R_p)-**8** from (R_p,M,R_p)-**7** (100 mg, 0.18 mmol), 53 mg (36%) of the title compound was obtained.
$[\alpha]_D^{20}$= +10.9 (c 0.46, $CHCl_3$).

Chart S12. Chromatogram of (*rac*)-**8**

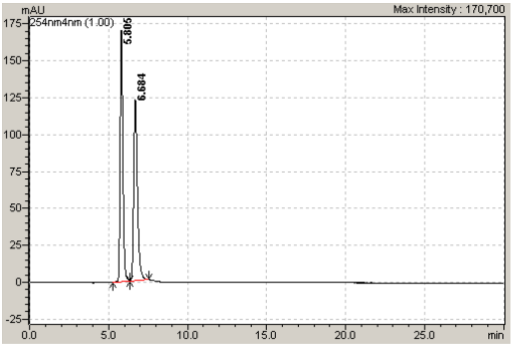

	Retention time	Peak start	Peak end	Area%
1	5.805	5.248	6.368	50
2	6.684	6.368	7.541	50

Chart S13. Chromatogram of (S_p,P,S_p)-**8** (left chart) and (R_p,M,R_p)-**8** (right chart)

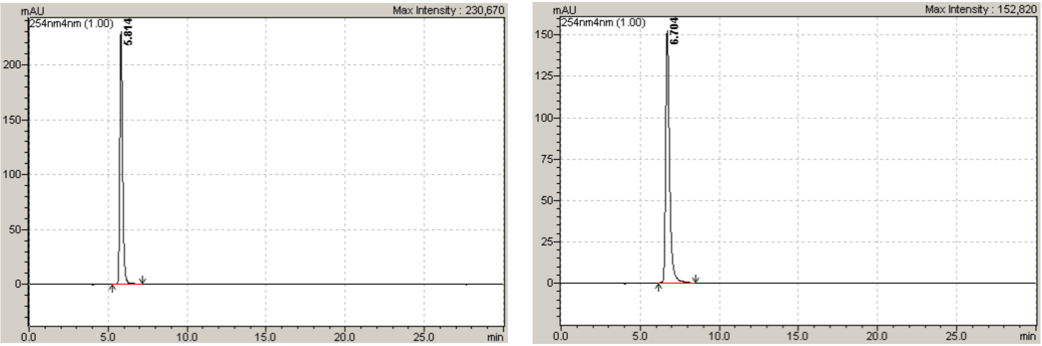

	Retention time	Peak start	Peak end	Area%
(S_p,P,S_p)-**8**	5.814	5.280	7.147	>99
(R_p,M,R_p)-**8**	6.704	6.144	8.501	>99

Dispiro[fluorene-9,5'-indeno[2,1-c]fluorene-8',9''-fluorene] (DSIF).

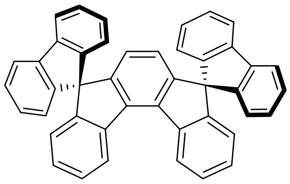

The same procedure as above was used for synthesis of **DSIF** from the maternal diketone (92 mg, 0.33 mmol). Flash column chromatography provided 100 mg (55%) of the title compound as a colorless solid.
R_f (4/1 hexanes/CH_2Cl_2) = 0.29.
^{1}H NMR (400 MHz, $CDCl_3$) δ 8.72 (d, J = 7.9 Hz, 2H), 7.82 (d, J = 7.6 Hz, 4H), 7.54 (t, J = 7.5 Hz, 2H), 7.35 (t, J = 7.5 Hz, 4H), 7.20 (t, J = 7.5 Hz, 2H), 7.11 (t, J = 7.5 Hz, 4H), 6.86–6.80 (m, 6H), 6.42 (s, 2H).
^{13}C{^{1}H} NMR (101 MHz, $CDCl_3$) δ 149.8, 149.7, 149.2, 142.2, 141.9, 137.1, 128.0, 127.9, 127.8, 127.7, 124.6, 124.3, 123.7, 123.4, 120.1, 66.2.
The recorded data agree with the reported values.[4]

[4] Romain, M.; Thiery, S.; Shirinskaya, A.; Declairieux, C.; Tondelier, D.; Geffroy, B.; Jeannin, O.; Rault-Berthelot, J.; Métivier, R.; Poriel, C. *Angew.Chem. Int.Ed.* **2015**, *54*, 1176–1180.

5 Photophysical Properties

UV−vis absorption spectra were recorded on Thermo Scientific Helios γ with wolfram and deuterium lamp. Steady-state fluorescence spectra were monitored on an FLS 980 spectrofluorometer (Edinburgh Instruments). Fluorescence quantum yields were determined using a Quantaurus-QY Plus spectrofluorometer with integrating sphere (HamamatsuC13534-33). Samples for fluorescence measurements were prepared as CH_2Cl_2 solutions in long-neck 1 cm path-length cuvettes. Absorbance was 0.1 or less at the excitation wavelength.

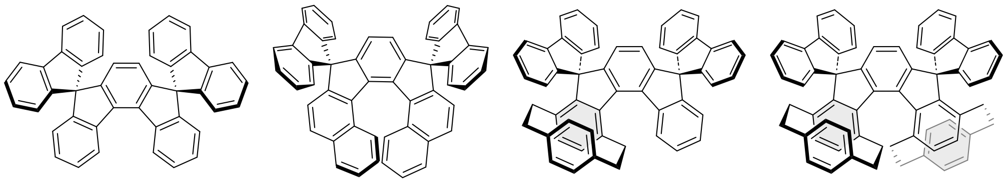

Figure S1. Structures of dispiro-compounds **DSIF**, **[7]-helical DSIF**, **4**, and **8**.

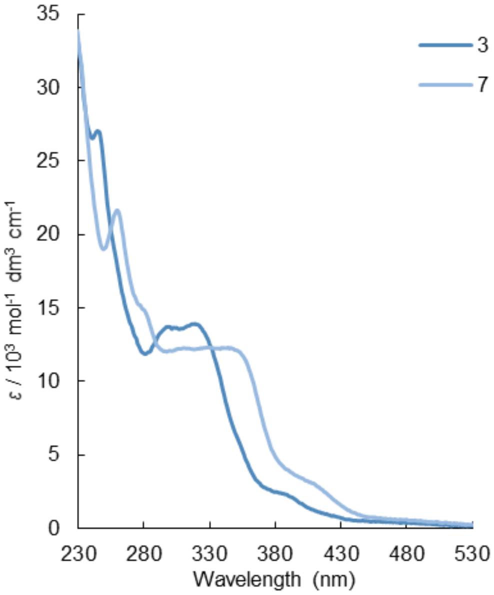

Figure S2. UV-vis spectra of diketones **3** and **7**, solutions in CH_2Cl_2 ($c = 1 \times 10^{-5}$ M).

Table S3. Photophysical properties of **4**, **8**, and their comparison with structurally related compounds.

Compound	λ_{abs}, nm (ε_λ, $\times 10^4 \cdot mol^{-1} \cdot dm^3 \cdot cm^{-1}$)	λ_{lum} (nm)	Φ_{lum} (%)
**DSIF**	261 (7.76), 309 (6.54), 317 (4.76), 339 (0.99)	346, 360	87
**[7]-helical DSIF**	299 (1.71), 312 (2.06), 333 (1.27), 370 (2.45), 388 (2.10)	409, 423	87
rac-**4**	297 (1.72), 311 (2.11), 333 (1.75)	386	41
rac-**8**	314 (2.23), 348 (1.86), 362 (1.87)	410	61

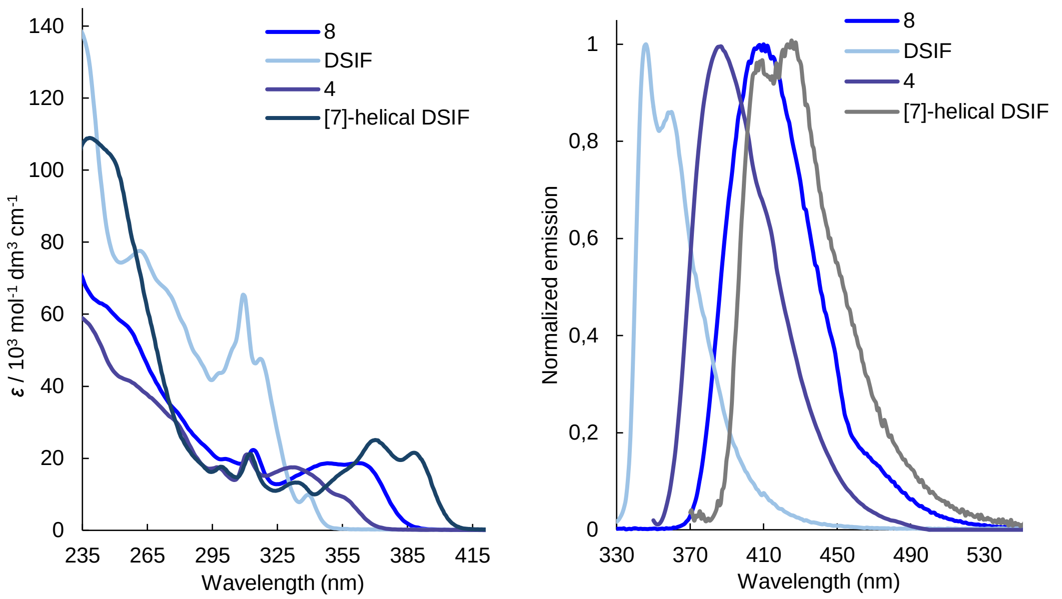

Figure S3. UV-vis spectra of DSIF derivatives, solutions in CH_2Cl_2 ($c = 1 \times 10^{-5}$ M) and emission spectra of compounds **4**, **8**, **DSIF**, and **[7]-helical** solutions in CH_2Cl_2 (λ_{exc} = 310 nm, 350 nm for [7]-helical).

6 Chiroptical Properties

ECD spectra of CH_2Cl_2 solutions (10^{-6} M) of (S_p)/(R_p)-**4** and (S_p,P,S_p)/(R_p,M,R_p)-**8** were measured at room temperature using a Jasco J-815 instrument. CPL measurements were performed using a home-built CPL spectrofluoropolarimeter (constructed with the help of the JASCO Company). The samples were excited using a 90° geometry with a 150 W LS Xenon ozone-free lamp. The concentrations of the samples were measured at ca. 10^{-5} M in dichloromethane at room temperature. Sample solutions of **4** were excited at 334 nm (15 accumulations for each enantiomer) and of **8** at 315 nm (12 accumulations for each enantiomer).

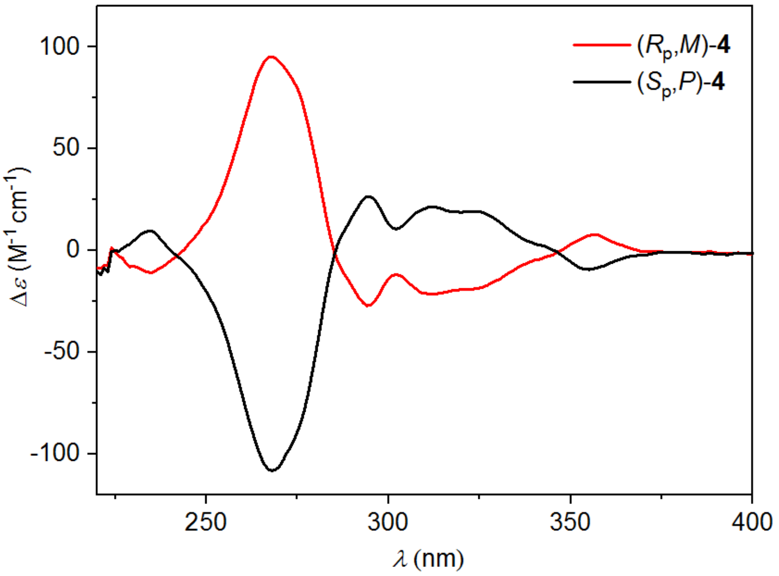

Figure S4. ECD spectra of **4**.

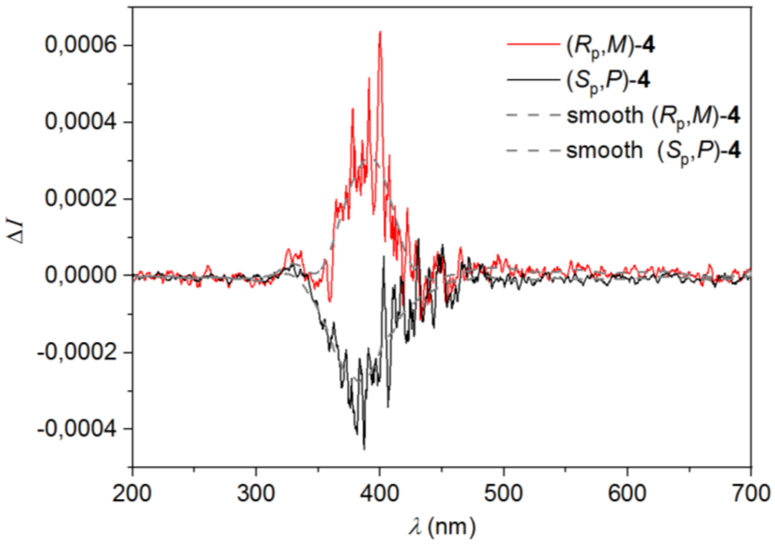

Figure S5. CPL spectra of **4**.

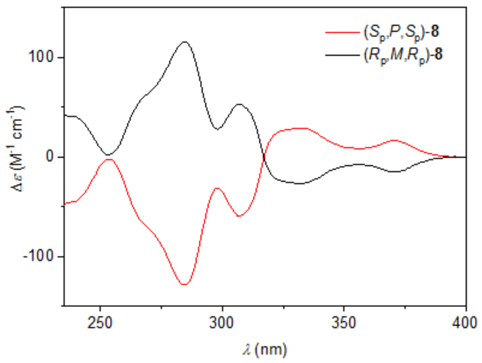

Figure S6. ECD spectra of **8**.

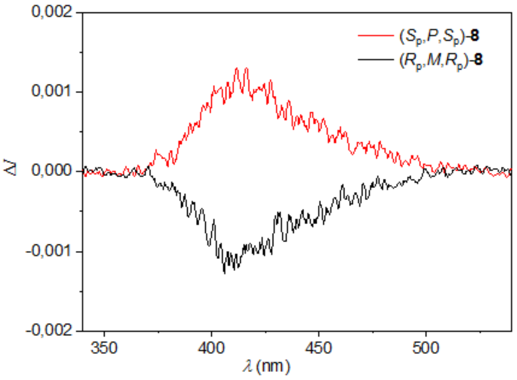

Figure S7. CPL spectra of **8**.

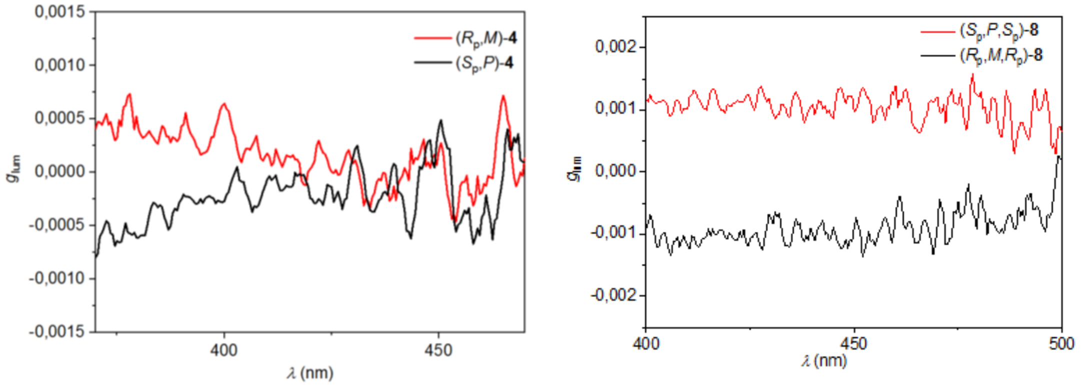

Figure S8. Emission dissymmetry factor spectra for **4** and **8** enantiomers.

The performance of chiral emitters can be evaluated by several parameters. The most often used parameters to evaluate chiral emitters are photoluminescence quantum yield (Φ_{PL}), luminescence dissymmetry factor (g_{lum}), and CPL brightness (B_{CPL}), see below:[5, 6]

$$g_{\mathrm{lum}} = 2\frac{I_{\mathrm{L}} - I_{\mathrm{R}}}{I_{\mathrm{L}} + I_{\mathrm{R}}} \quad (\mathrm{Eq.\,1}) \qquad \text{or} \qquad g_{\mathrm{lum}} = \frac{4\,|\mu_{ij}|\,|\boldsymbol{m}_{ji}|\,cos\,\theta_{ij}}{|\mu_{ij}^2| + |\boldsymbol{m}_{ji}^2|} = \frac{4R}{D} \quad (\mathrm{Eq.\,2})$$

I_{L} and I_{R} = the emission intensities of left and right-handed circularly polarized light.

[5] Mori, T. in Circularly Polarized Luminescence of Isolated Small Organic Molecules. (Ed. Mori, T.), Springer Nature Singapore Pte Ltd, **2020**, Ch. 1, 1-10.
[6] Arrico, L; Di Bari, L.; Zinna, F. *Chem. Eur. J.* **2021**, *27*, 2920–2934.

For $i \rightarrow j$ transition

μ_{ji} and $\boldsymbol{m}_{ji}$	=	the electric and magnetic transition dipole vectors
θ_{ij}	=	the angle between μ_{ji} and m_{ji}.

$$B_{CPL} = \left| \frac{1}{2} \times \varepsilon_\lambda \times \Phi \times g_{lum} \right|$$

ε_λ	=	molar extinction coefficient
Φ	=	luminescence quantum yield (0–1.0)
g_{lum}	=	luminescence dissymmetry factor

Table S4. Dissymmetry factors (g_{lum}) of dispiroindeno[2,1-*c*]fluorene derivatives.[a]

Compound	λ_{ext}[b] nm (ε_λ)[c]	Φ_{lum}	g_{lum} (× 10^{-3})	B_{CPL}
(S_p,*P*)-**4**	334 (1.73)		-0.3	1.06
(R_p,*M*)-**4**	334 (1.73)		0.3	1.06
(S_p,*P*,S_p)-**8**	315 (2.28)	0.61	-1.1	7.65
(R_p,*M*,R_p)-**8**	315 (2.28)	0.61	1.1	7.65

[a] Dichloromethane solution, $c \approx 1 \times 10^{-5}$ M.
[b] Excitation wavelength.
[c] Molar absorption coefficient (× $10^4 \cdot mol^{-1} \cdot dm^3 \cdot cm^{-1}$)

Calculation of g_{lum} factor for **4** and **8**.

Density Functional Theory (DFT) computational studies were done using Gaussian 16, Revision C.01. Computations utilized the M06 functional and the Def2TZVP basis set on all atoms. Geometry optimization of the first excited-state geometries used time-dependant DFT computations considering eight states. Empirical dispersion was accounted for by the D3 version of Grimme's dispersion correction. Effects of dichloromethane as solvent were included by employing the Solvent Accessible Surface model.

4, calculated $g_{lum} = 0.42 \times 10^{-3}$
8, calculated $g_{lum} = 1.15 \times 10^{-3}$

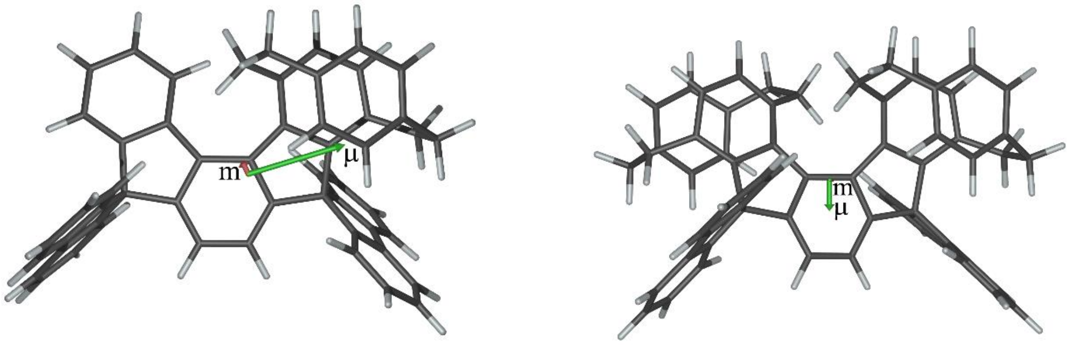

Figure S9. Simulation of electronic and magnetic transition dipole moments of **4** (left, (angle between electric dipole and magnetic vectors: 100.7°) and **8** (right, (angle between electric dipole and magnetic vectors: 176.6°).

7 X-ray Diffraction Data

The diffraction data of single crystals of *rac*-**2**, **3**, **S6,** *rac*-**4**, *rac*-**7**, **8** were recorded on Bruker D8 VENTURE Kappa Duo PHOTONIII by IµS micro-focus sealed tube either MoKα (λ = 0.71073Å) or CuKα (λ = 1.54178 Å) at 120 K (temperature of the crystal preserved by Cryostream Cooler 800). The structures were solved by direct methods (XT)[7] and refined by full matrix least squares based on F^2 (SHELXL2019).[8] The hydrogen atoms on carbons were fixed into idealized positions (riding model) and assigned temperature factors $H_{iso}(H) = 1.2\ U_{eq}$ (pivot atom).

The structures of most samples were determined by standard procedure, however some of them required detailed description.

The crystal of **8** contains disordered molecules of chloroform. In one position this molecule was possible to be resolved by splitting chlorine atoms into several partially occupied positions. However, satisfactory disorder model for other molecules was not found, therefore, to improve the precision of the principal molecule the SQUEEZE procedure within PLATON[9] was applied to remove the contribution of solvent to diffraction data.

X-ray crystallographic data have been deposited at the Cambridge Crystallographic Data Centre (CCDC) can be obtained free of charge from the Centre via its website (www.ccdc.cam.ac.uk/structures/). The corresponding ccdc numbers are in Tables S5-S9.

---

[7] SHELXT: Sheldrick, G.M. (2015). *Acta Cryst*. **A71**, 3-8.
[8] SHELXL: Sheldrick, G.M. (2015). *Acta Cryst.* **C71**, 3-8.
[9] PLATON Spek,A.L. (2009) Acta Cryst., D65, 148-155.

Table S5. Crystal data, data collection, and refinement parameters for **2** and **3**.

Compound	**2**	**3**
CCDC	2463385	2463377
Formula	$C_{30}H_{24}O_2$	$C_{30}H_{20}O_2$
M.W.	416.49	412.46
Crystal system	Monoclinic	Monoclinic
Space group	$P2_1/c$ (No.14)	$P2_1/c$ (No.14)
a [Å]	12.0425 (6)	8.1279 (4)
b [Å]	11.0539 (4)	19.1837 (9)
c [Å]	16.2750 (7)	13.1760 (6)
α [°]		
β [°]	104.812 (2)	107.264 (1)
γ [°]		
Z	4	4
V[Å^3]	2094.48 (16)	1961.89 (16)
Temperature	120	120
D_x [g cm^{-3}]	1.321	1.396
Wavelength, Å	0.71073	1.54178
Crystal size [mm]	0.57 × 0.36 × 0.22	0.57 × 0.41 × 0.23
Crystal color, shape	Prism, orange	Prism, orange
μ [mm^{-1}]	0.08	0.68
T_{min},T_{max}	0.705, 0.746	0.698, 0.858
Measured reflections	25378	17332
Independent diffractions (R_{int}^a)	4791, (0.025)	3819, (0.037)
Observed diffract. [I>2σ(I)]	4492	3693
No. of parameters	289	289
R^b	0.039	0.037
$wR(F^2)$ for all data	0.101	0.090
GOF^c	1.02	1.04
Residual electron density [e/Å^3]	0.52, -0.27	0.25, -0.21

$^aR_{\text{int}} = \Sigma|F_o^2 - F_{o,\text{mean}}^2|/\Sigma F_o^2$;
$^bR(F) = \Sigma||F_o| - |F_c||/\Sigma|F_o|$; $wR(F^2) = [\Sigma(w(F_o^2 - F_c^2)^2)/(\Sigma w(F_o^2)^2)]^{½}$;
$^c\text{GOF} = [\Sigma(w(F_o^2 - F_c^2)^2)/(N_{\text{diffrs}} - N_{\text{params}})]^{½}$

Table S6. Crystal data, data collection, and refinement parameters for **S6**, **4**, and **7**.

**Compound**	**S6**	**4**	**7**
CCDC	2463379	2463380	2463381
Formula	$C_{30}H_{22}O$	$C_{54}H_{36}$	$C_{40}H_{30}O_2$
M.w.	398.47	684.83	542.64
Crystal system	Monoclinic	Monoclinic	Monoclinic
Space group	$P2_1/n$ (No. 14)	$C2/c$ (No.15)	$P2_1/n$ (No.14)
a [Å]	8.0074 (2)	28.1149 (7)	14.2019 (6)
b [Å]	19.1256 (4)	14.0802 (4)	14.4609 (6)
c [Å]	13.3210 (3)	19.8172 (6)	14.3051 (6)
α [°]			
β [°]	106.797 (1)	116.212 (1)	113.140 (1)
γ [°]			
Z	4	8	4
$V [Å^3]$	1953.02 (8)	7038.2 (3)	2701.5 (2)
Temperature [K]	120	120	120
D_x [g cm^{-3}]	1.355	1.293	1.334
Wavelength [Å]	0.71073	1.54178	1.54178
Crystal size [mm]	0.40 × 0.25 × 0.24	0.61 × 0.05 × 0.03	0.30 × 0.16 × 0.12
Crystal color, shape	Prism, yellow	Bar, colourless	Prism, orange
μ [mm^{-1}]	0.08	0.55	0.63
T_{min}, T_{max}	0.968, 0.981	0.728, 0.984	0.835, 0.929
Measured reflections	43976	37198	49673
Independent diffractions(R_{int}^a)	4484, (0.028)	6381, (0.042)	5797, (0.031)
Observed diffract. [I>2σ(I)]	4265	5495	5430
No. of parameters	280	487	379
R^b	0.039	0.038	0.039
$wR(F^2)$ for all data	0.098	0.100	0.100
GOF^c	1.05	1.02	1.04
Residual electron density [e/Å^3]	0.34, −0.20	0.21, -0.20	0.28, -0.20

$^aR_{int} = \Sigma|F_o^2 - F_{o,mean}^2|/\Sigma F_o^2$; $^bR(F) = \Sigma||F_o| - |F_c||/\Sigma|F_o|$; $wR(F^2) = [\Sigma(w(F_o^2 - F_c^2)^2)/(\Sigma w(F_o^2)^2)]^{½}$;

$^c\mathrm{GOF} = [\Sigma(w(F_o^2 - F_c^2)^2)/(N_{diffrs} - N_{params})]^{½}$

Table S7. Crystal data, data collection, and refinement parameters for *meso*-**7** and **8**.

Compound	***meso*-7**	**8**
CCDC	2463382	2463383
Formula	$C_{40}H_{30}O_2 \cdot 0.090(H_2O)$	$2(C_{64}H_{46}) \cdot CHCl_3$
M.w.	544.26	1749.38
Crystal system	Triclinic	Monoclinic
Space group	*P* -1 (No. 2)	$P2_1/n$ (No. 14)
a [Å]	7.2260 (2)	18.0113 (5)
b [Å]	13.5047 (3)	13.2622 (3)
c [Å]	13.6531 (3)	39.4772 (10)
α [°]	81.253 (1)	
β [°]	88.452 (1)	101.852 (1)
γ [°]	86.153 (1)	
Z	2	4
$V [Å^3]$	1313.68 (5)	9228.9 (4)
Temperature	120	120
D_x [g cm^{-3}]	1.376	1.259
Wavelength	1.54178	1.54178
Crystal size [mm]	0.26 × 0.17 × 0.12	0.32 × 0.26 × 0.06
Crystal color, shape	Prism, orange	Plate, colourless
μ [mm^{-1}]	0.65	1.32
T_{min},T_{max}	0.849, 0.929	0.679, 0.923
Measured reflections	31315	102265
Independent diffractions (R_{int}^a)	4979, (0.024)	16869, (0.039)
Observed diffract. [I>2σ(I)]	4796	15392
No. of parameters	383	1199
R^b	0.036	0.082
$wR(F^2)$ for all data	0.093	0.228
GOF^c	1.05	1.06
Residual electron density [e/Å^3]	0.22, −0.18	0.94, -1.10

$^aR_{int} = \Sigma|F_o^2 - F_{o,mean}^2|/\Sigma F_o^2$;
$^bR(F) = \Sigma||F_o|-|F_c||/\Sigma|F_o|$; $wR(F^2) = [\Sigma(w(F_o^2-F_c^2)^2)/(\Sigma w(F_o^2)^2)]^{½}$;
^{c}GOF $= [\Sigma(w(F_o^2-F_c^2)^2)/(N_{diffrs}-N_{params})]^{½}$

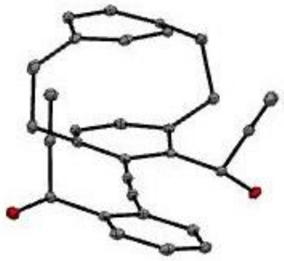

Figure S10. ORTEP drawing of **2**. Ellipsoids are drawn with 30% probability.

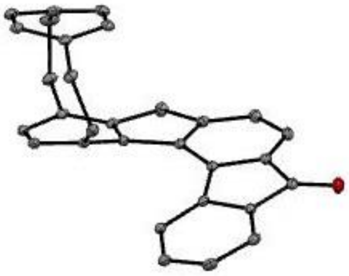

Figure S11. ORTEP drawing of **S6**. Ellipsoids are drawn with 30% probability.

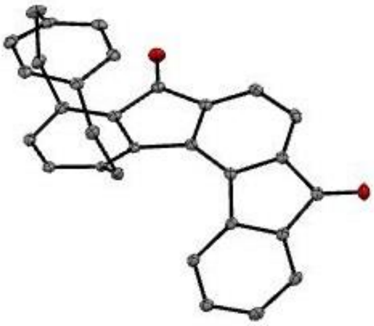

Figure S12. ORTEP drawing of **3**. Ellipsoids are drawn with 30% probability.

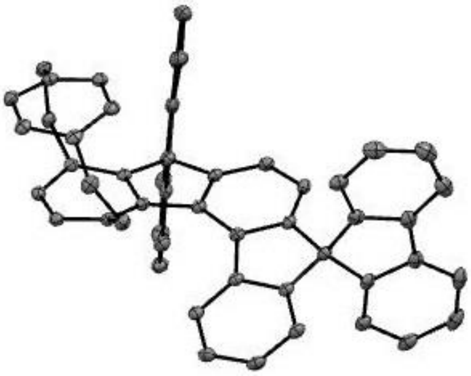

Figure S13. ORTEP drawing of **4**. Ellipsoids are drawn with 30% probability.

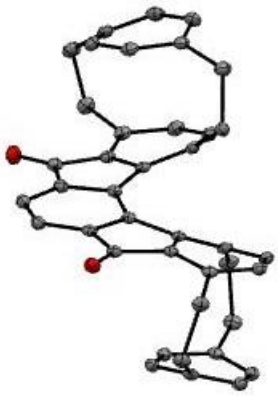

Figure S14. ORTEP drawing of *rac*-**7**. Ellipsoids are drawn with 30% probability.

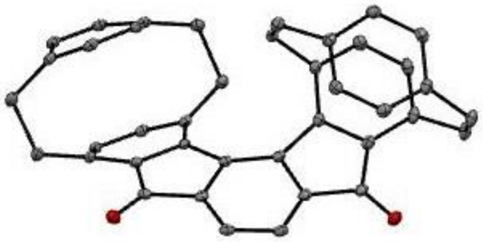

Figure S15. ORTEP drawing of *meso*-**7**. Ellipsoids are drawn with 30% probability.

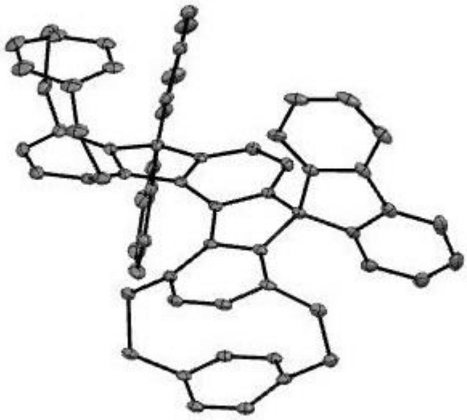

Figure S16. ORTEP drawing of **8**. Ellipsoids are drawn with 30% probability.

8 Copies of ^{1}H and $^{13}C\{^{1}H\}$ NMR Spectra

(*rac*)-4-Formyl[2.2]paracyclophane (1)

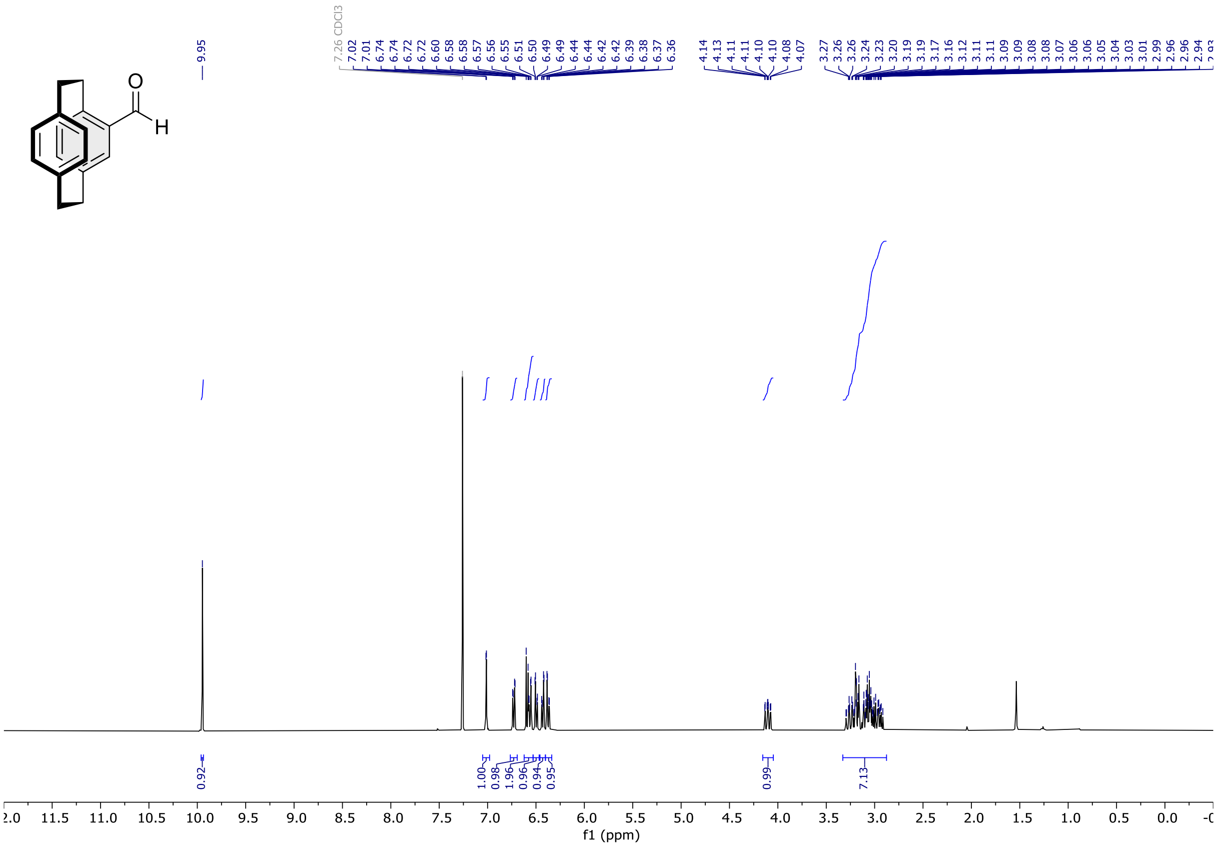

Figure S17. ^{1}H NMR spectrum of compound **1** ($CDCl_3$, 400 MHz).

(*rac*)-[2.2]Paracyclophan-*O*-methylaldoxime (S1)

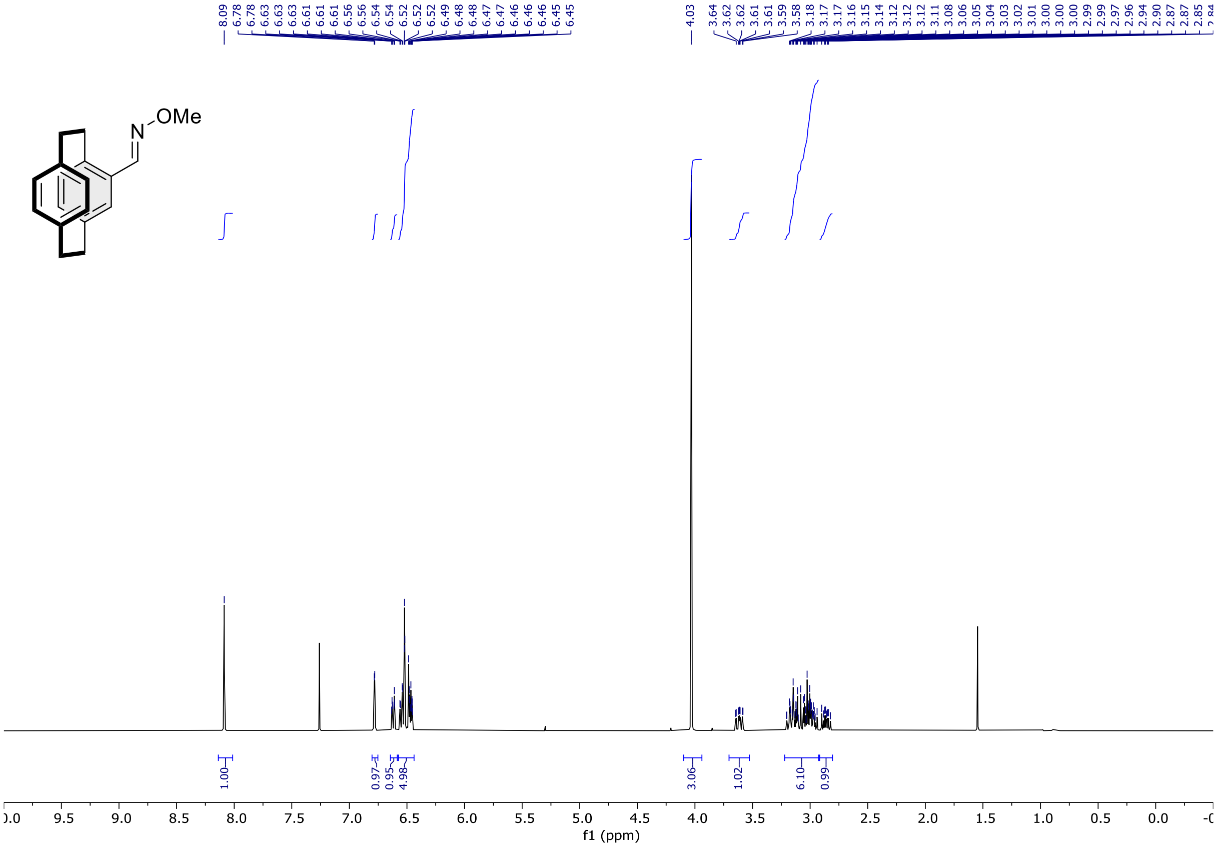

Figure S18. ^{1}H NMR spectrum of compound **S1** ($CDCl_3$, 400 MHz).

(*rac*)-13-iodo-1,4(1,4)-dibenzenacyclohexaphane-12-carbaldehyde O-methyl oxime (S2)

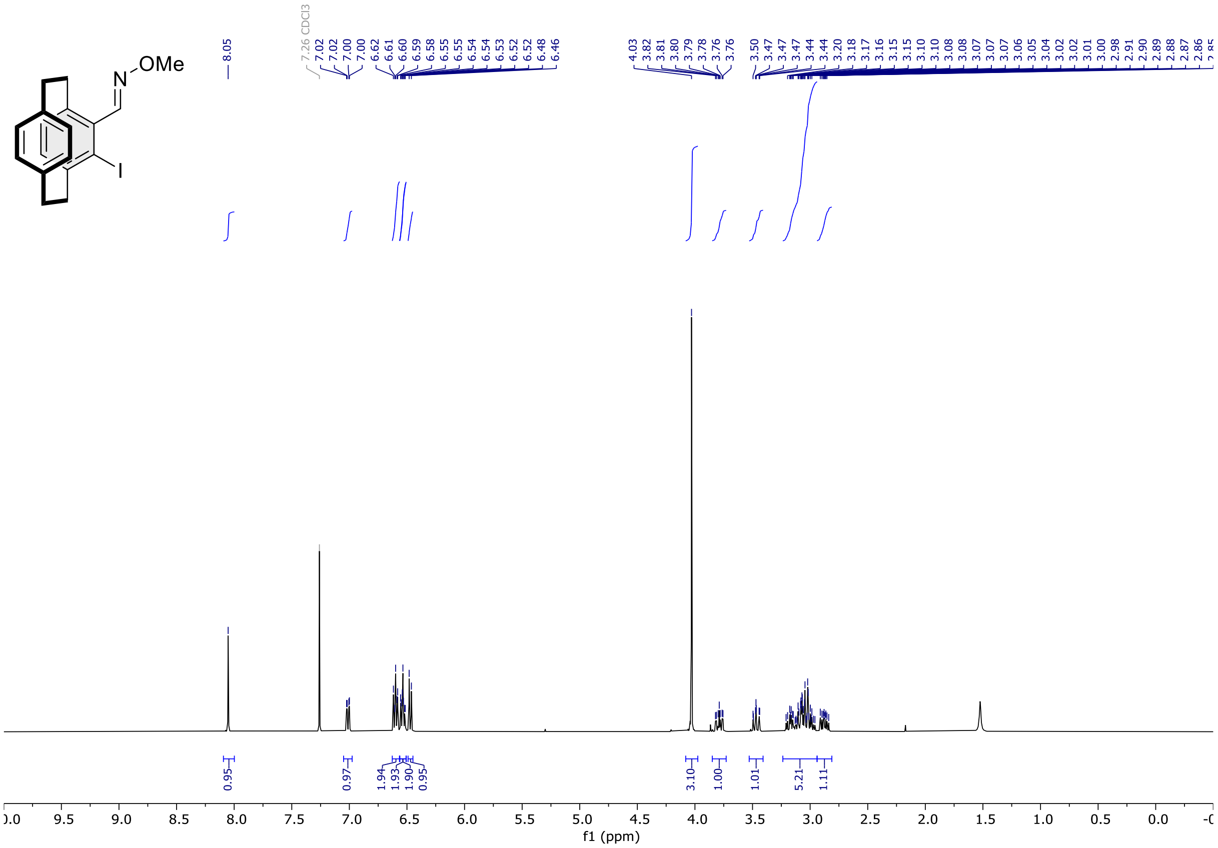

Figure S19. ^{1}H NMR spectrum of compound **S2** ($CDCl_3$, 400 MHz).

(*rac*)-4-Formyl-5-iodo[2.2]paracyclophane (S3)

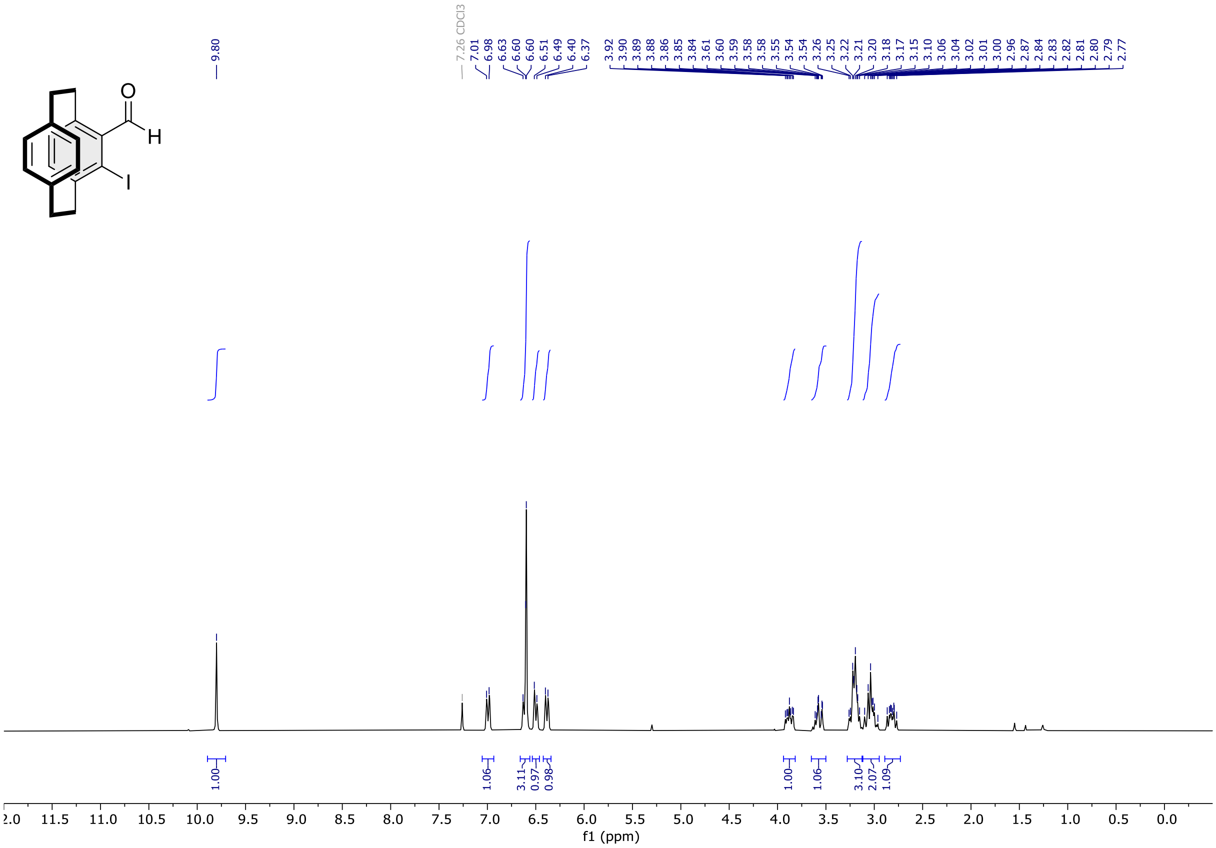

Figure S20. ^{1}H NMR spectrum of compound **S3** ($CDCl_3$, 400 MHz).

(*rac*)-13-ethynyl-1,4(1,4)-dibenzenacyclohexaphane-12-carbaldehyde (S4)

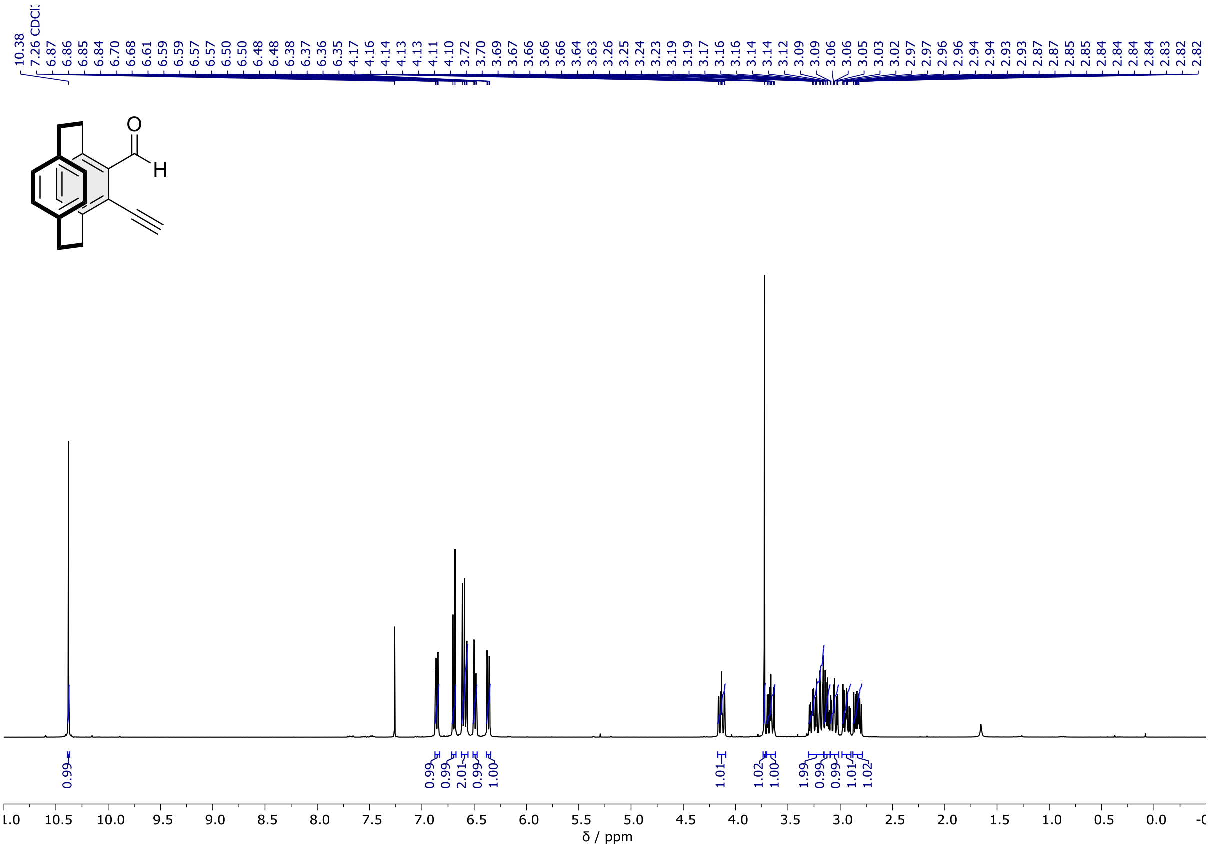

Figure S21. ^{1}H NMR spectrum of compound **S4** ($CDCl_3$, 400 MHz).

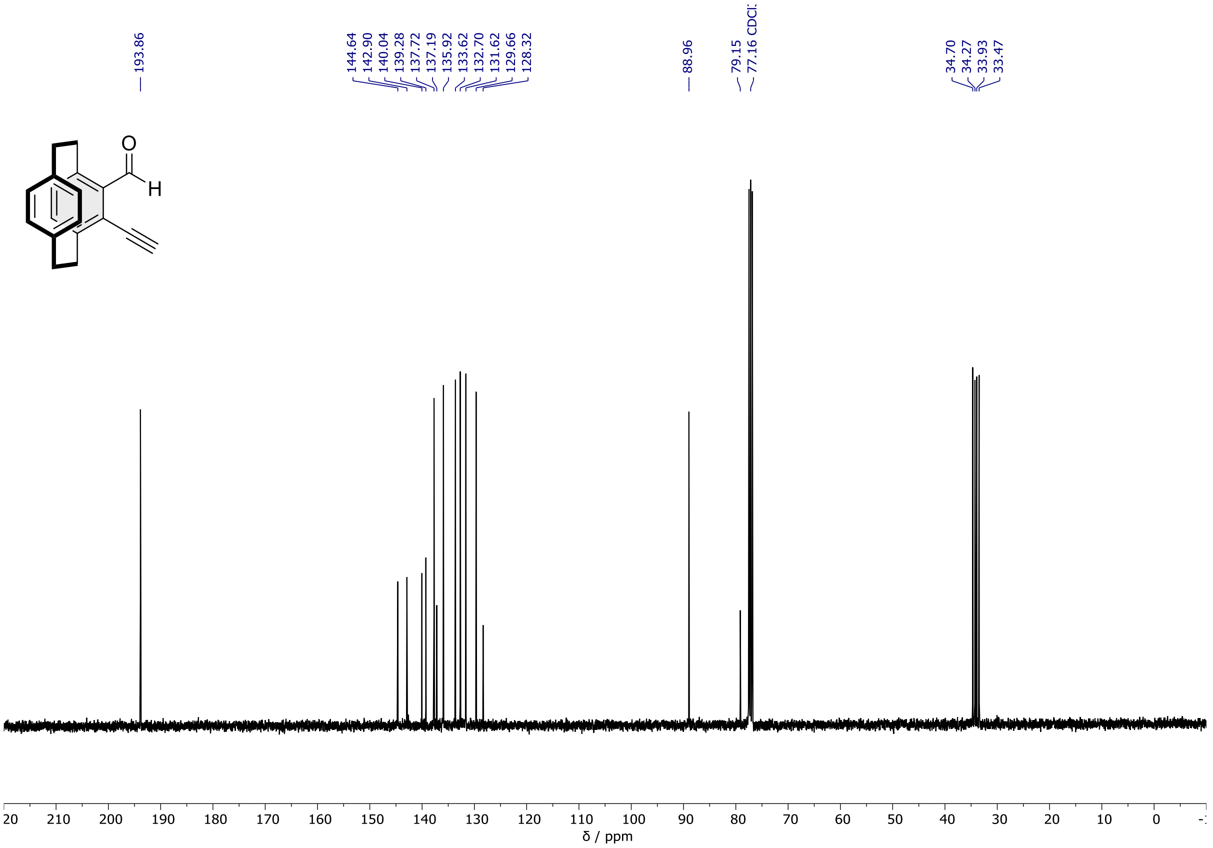

Figure S22. $^{13}C\{^{1}H\}$ NMR spectrum of compound **S4** ($CDCl_3$, 101 MHz).

(*rac*)-4-[2-(2-Formylphenyl)ethynyl]-5-formyl[2.2]paracyclophane (S5)

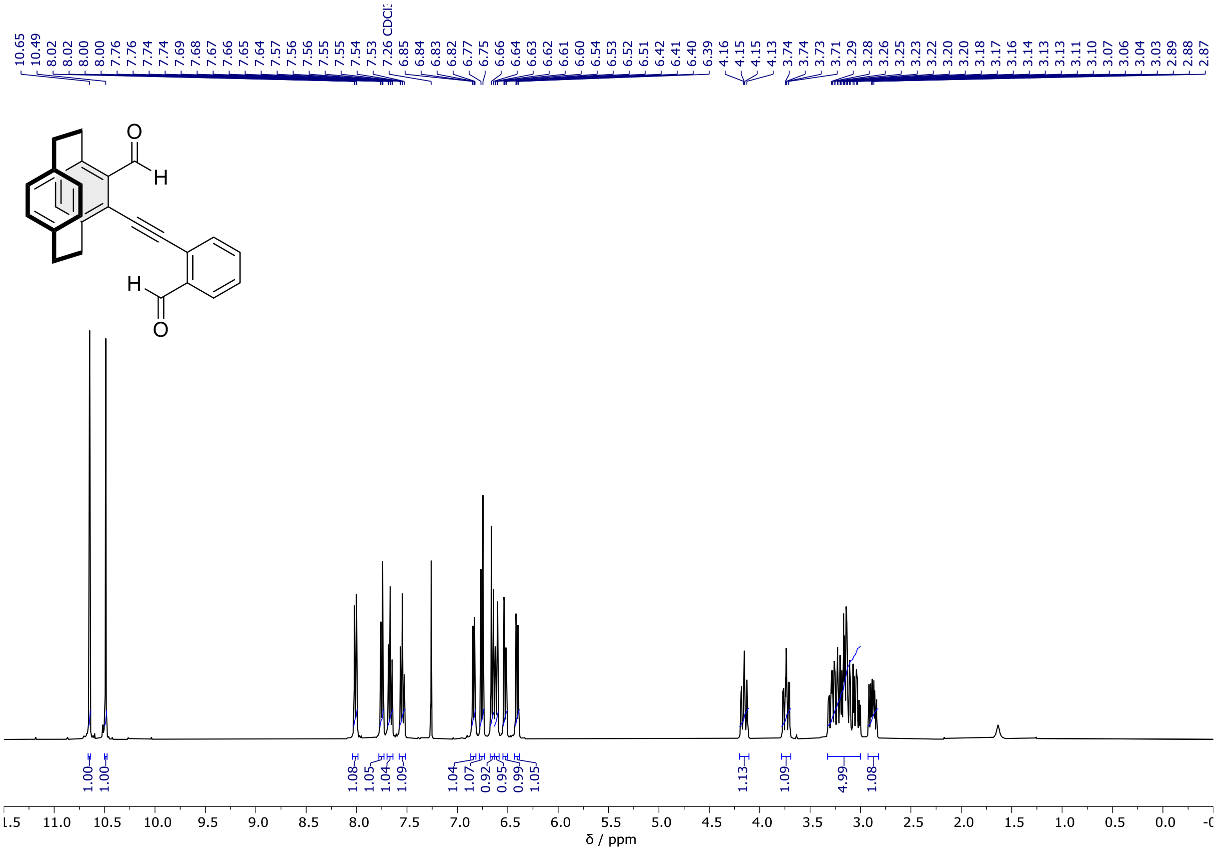

Figure S23. ^{1}H NMR spectrum of compound **S5** ($CDCl_3$, 400 MHz).

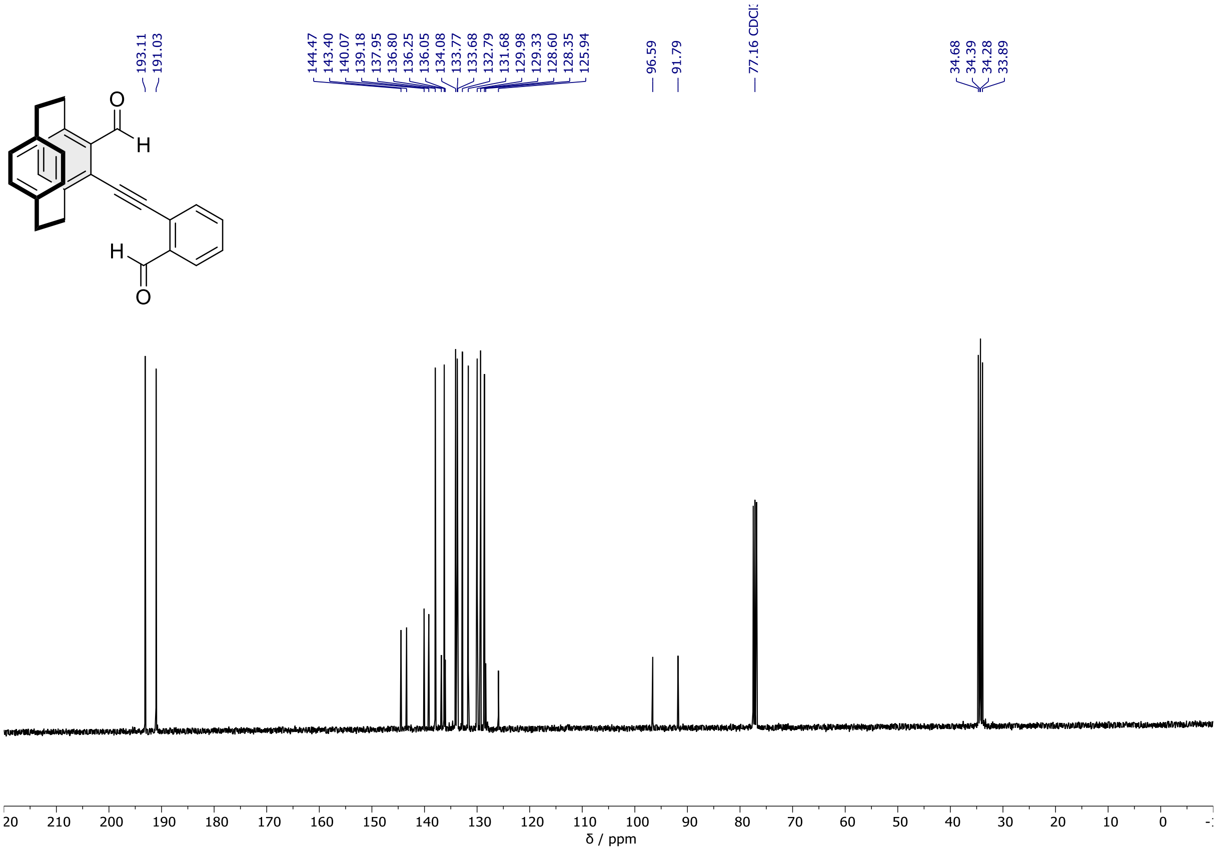

Figure S24. $^{13}C\{^{1}H\}$ NMR spectrum of compound **S5** ($CDCl_3$, 101 MHz).

1-(2-((1³-(1-hydroxyprop-2-yn-1-yl)-1,4(1,4)-dibenzenacyclohexaphane-1²-yl)ethynyl)phenyl)prop-2-yn-1-ol (2).

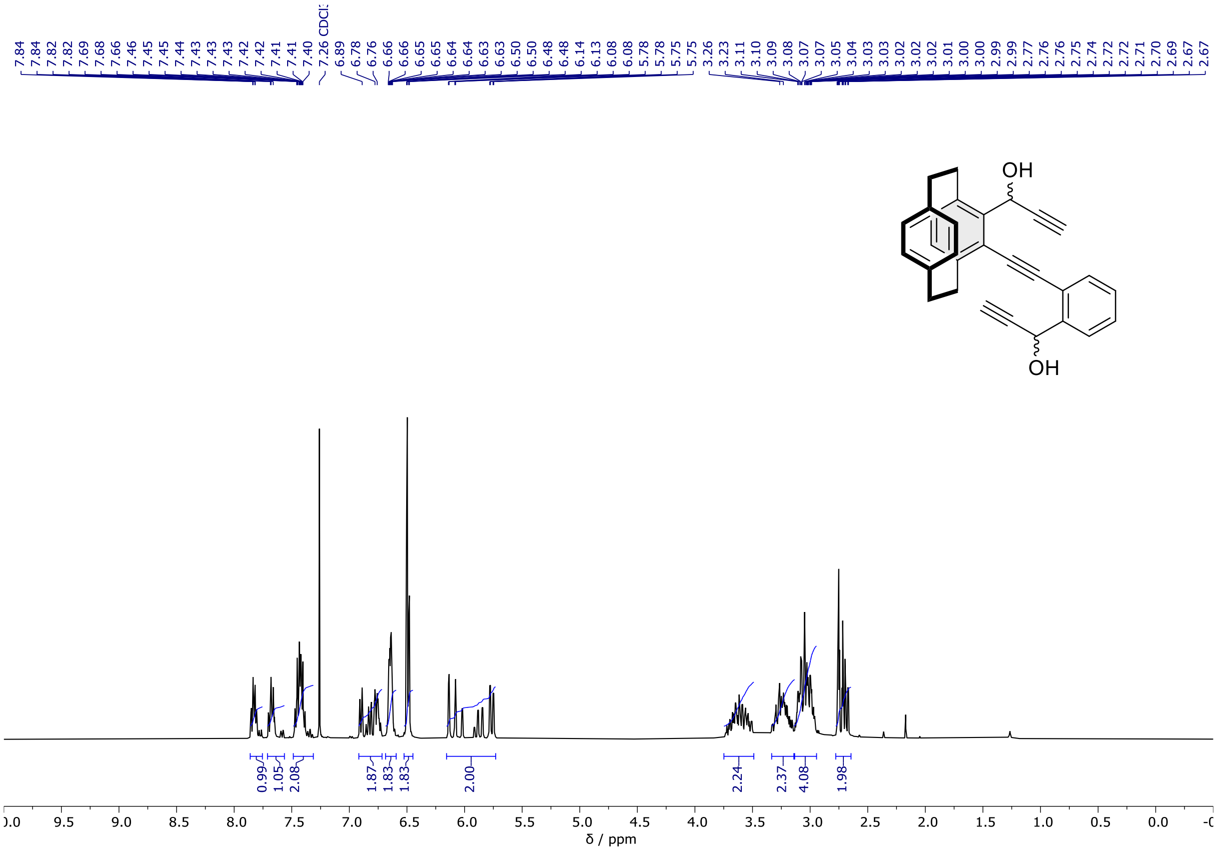

Figure S25. ^{1}H NMR spectrum of compound **2** ($CDCl_3$, 400 MHz).

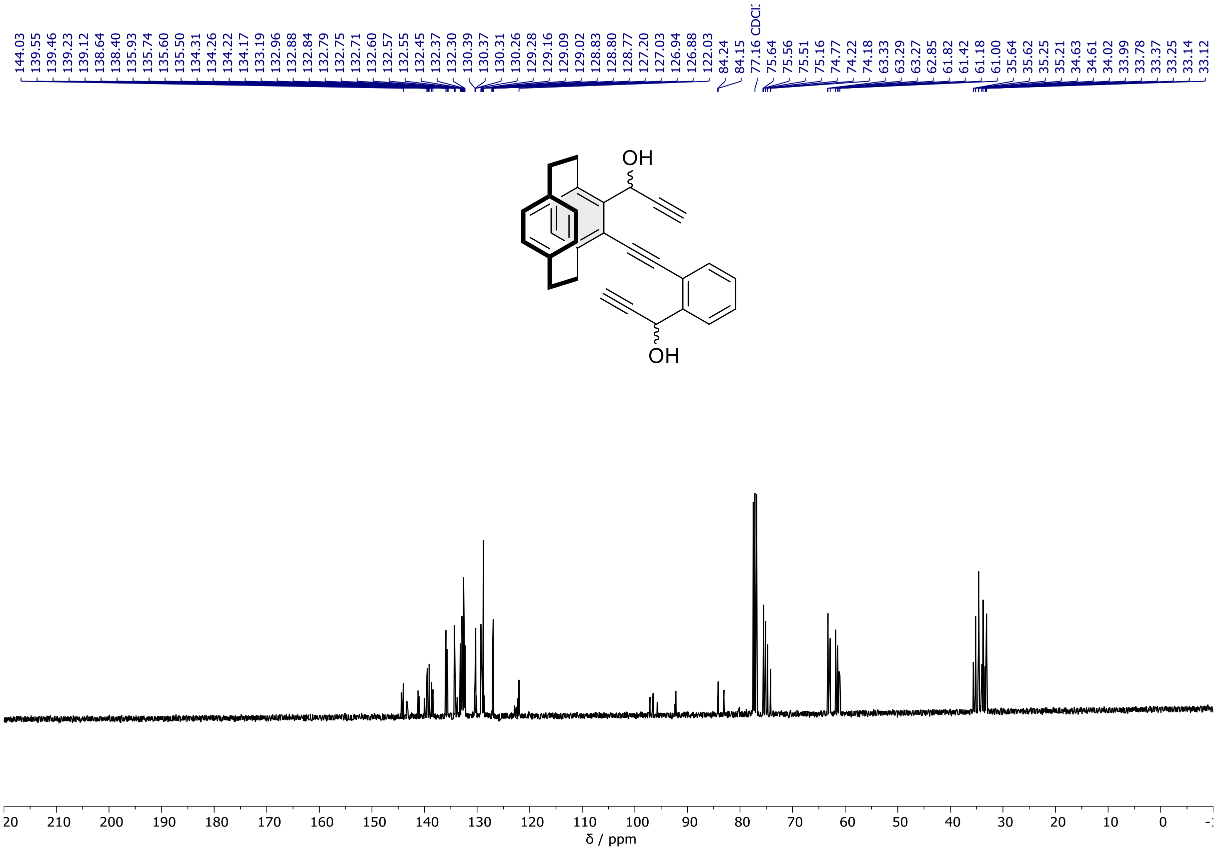

Figure S26. $^{13}C\{^{1}H\}$ NMR spectrum of compound **2** ($CDCl_3$, 101 MHz).

1⁵,1⁸-dihydro-1(1,4)-indeno[2,1-c]fluorena-4(1,4)-benzenacyclohexaphane-1⁵,1⁸-dione (3)

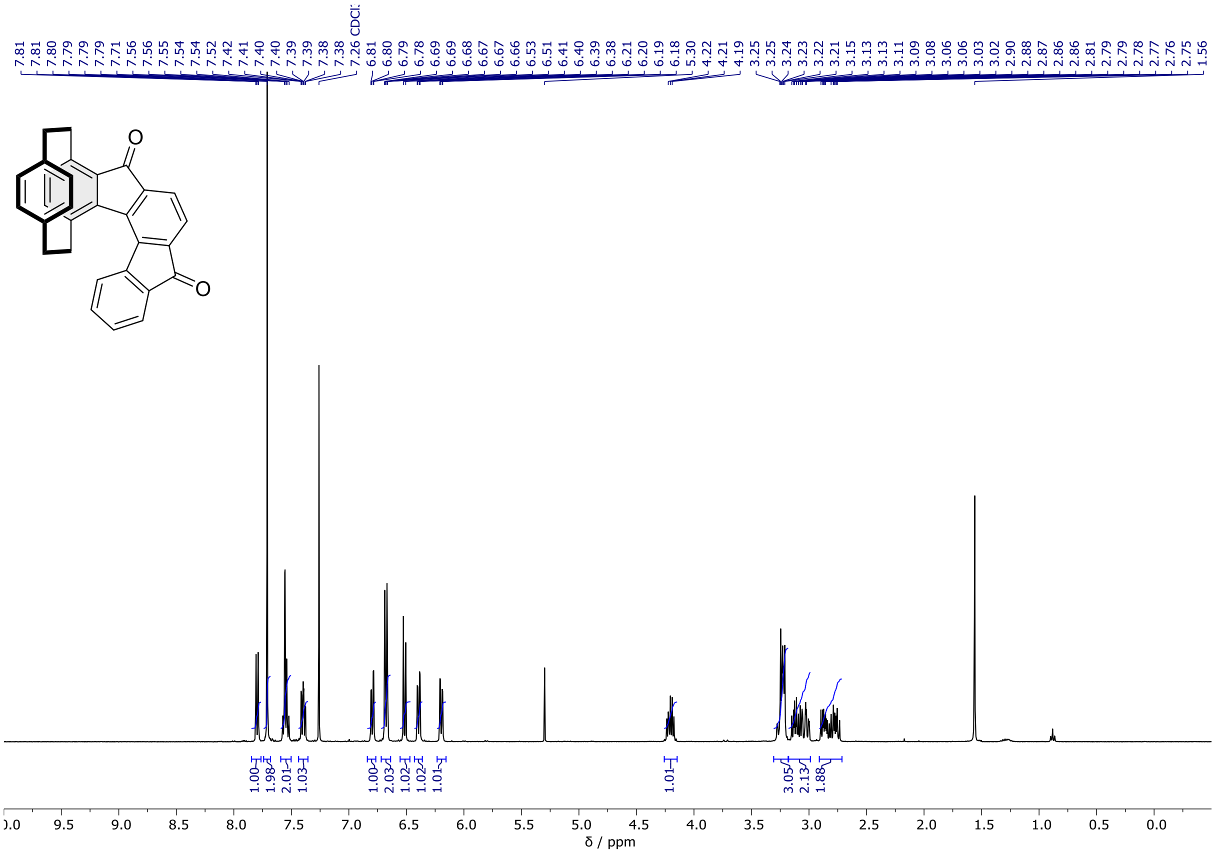

Figure S27. ^{1}H NMR spectrum of compound **3** ($CDCl_3$, 400 MHz).

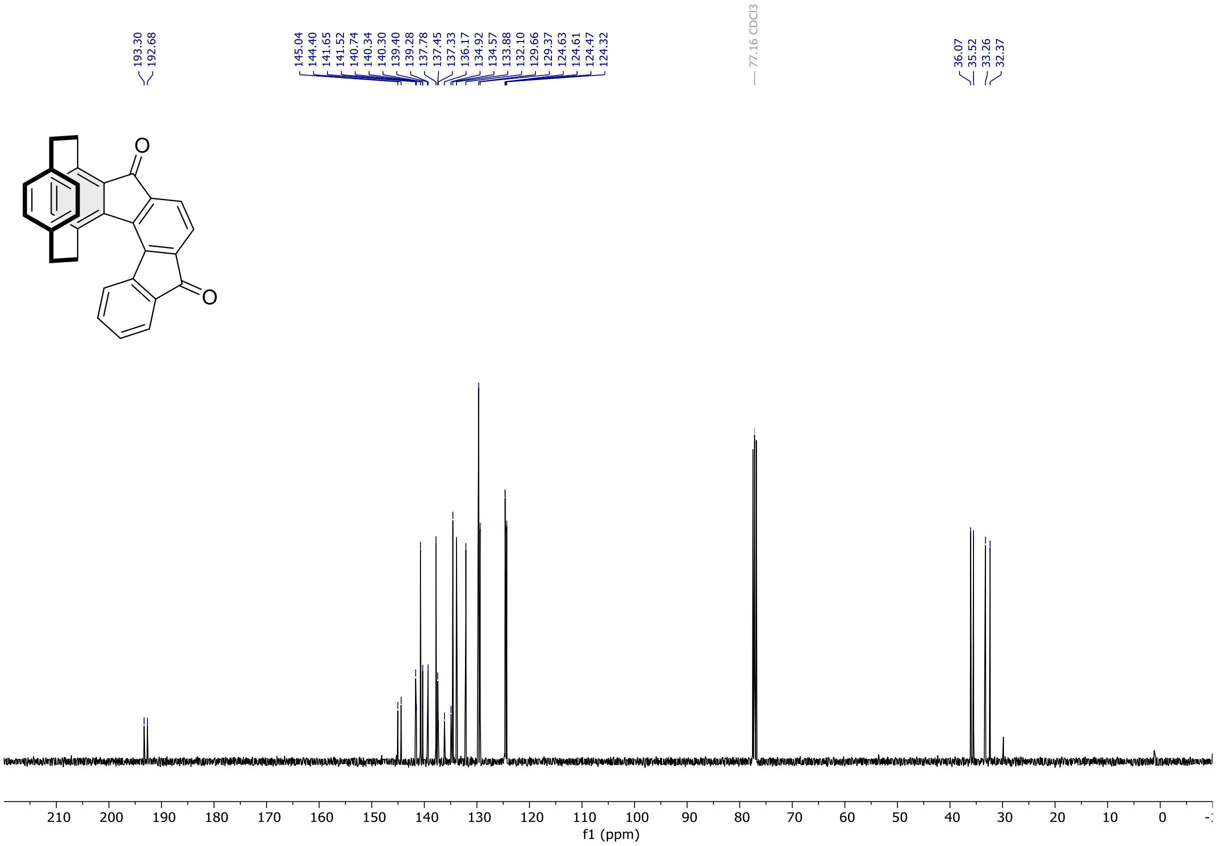

Figure S28. ^{13}C{^{1}H} NMR spectrum of compound **3** ($CDCl_3$, 101 MHz).

1⁵,1⁸-dihydro-1(1,4)-indeno[2,1-c]fluorena-4(1,4)-benzenacyclohexaphan-1⁸-one (S6)

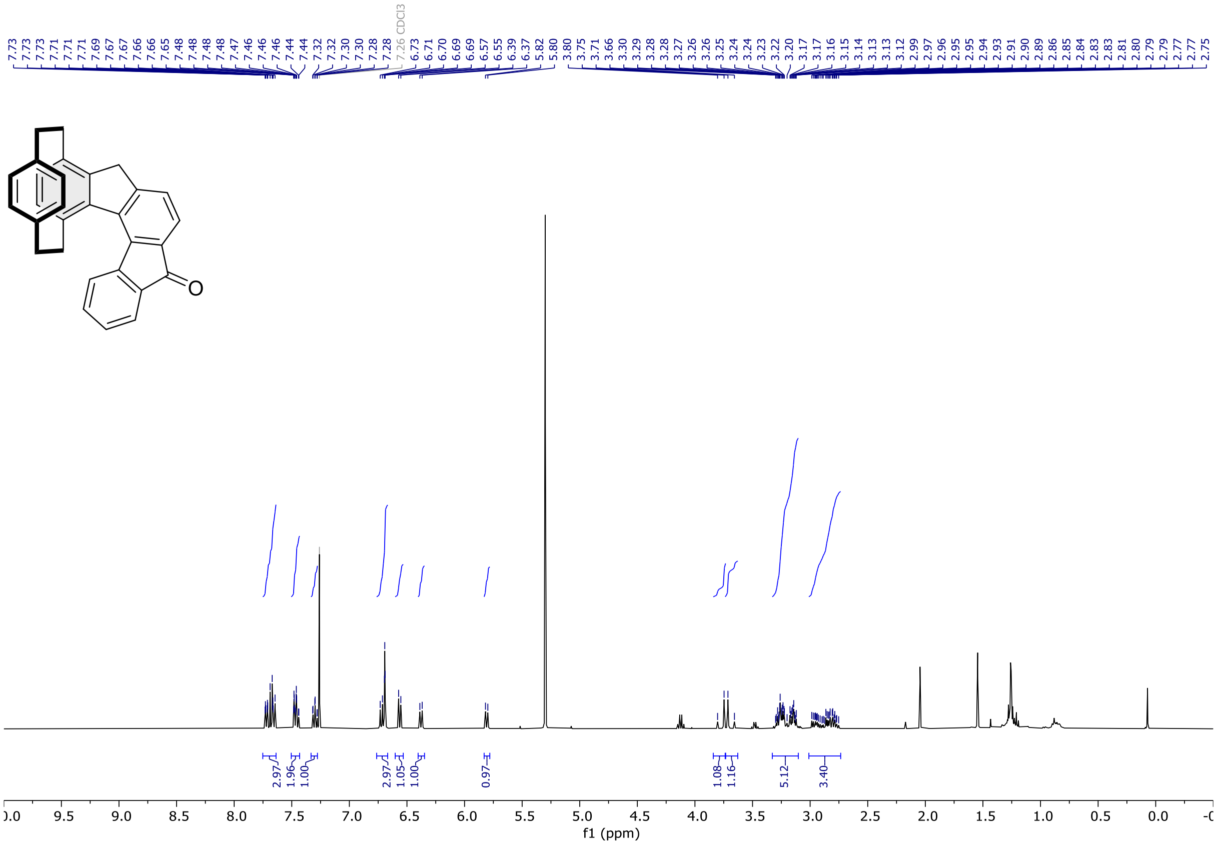

Figure S29. ^{1}H NMR spectrum of compound **S6** ($CDCl_3$, 400 MHz).

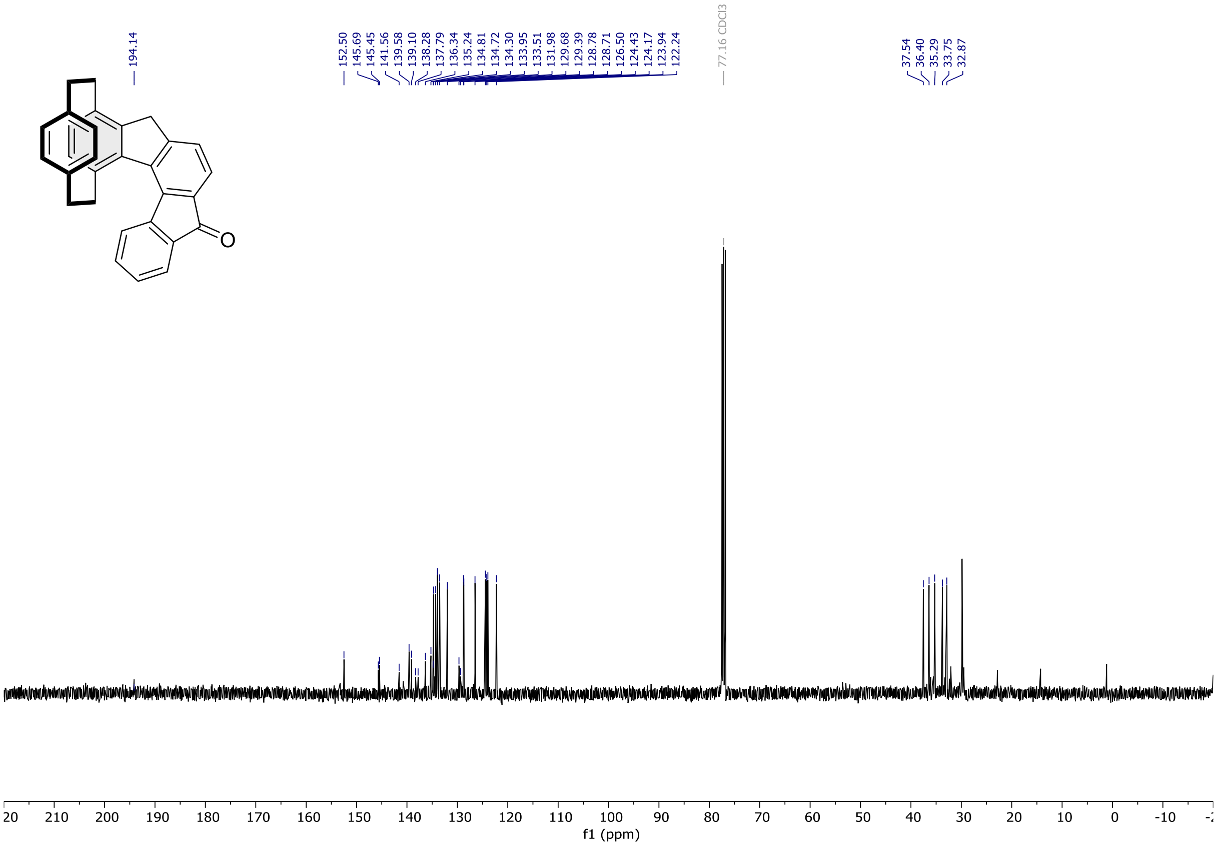

Figure S30. $^{13}C\{^{1}H\}$ NMR spectrum of compound **S6** ($CDCl_3$, 101 MHz).

Dispiro[fluorene-9,5'-1(1,4)-indeno[2,1-c]fluorena-4(1,4)-benzenacyclohexaphane-8',9''-fluorene] (4)

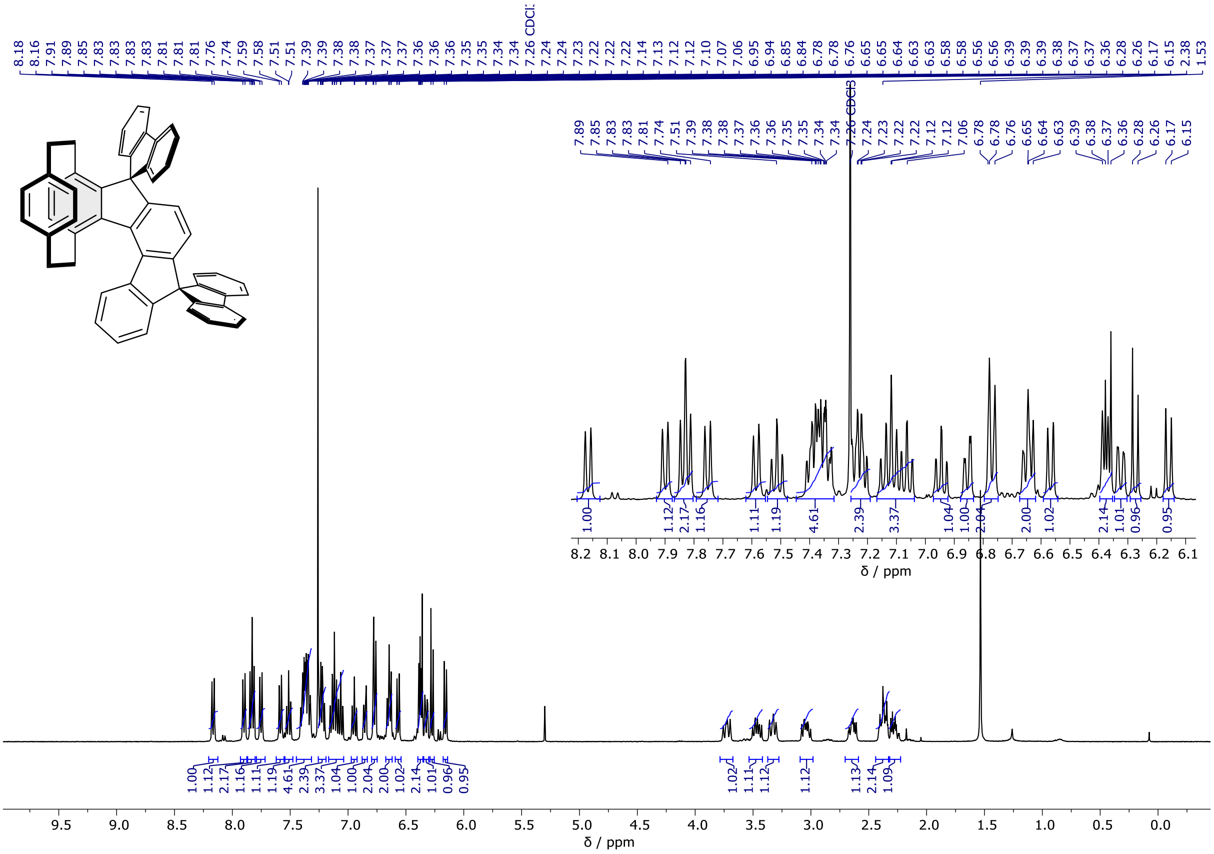

Figure S31. ^{1}H NMR spectrum of compound **4** ($CDCl_3$, 400 MHz).

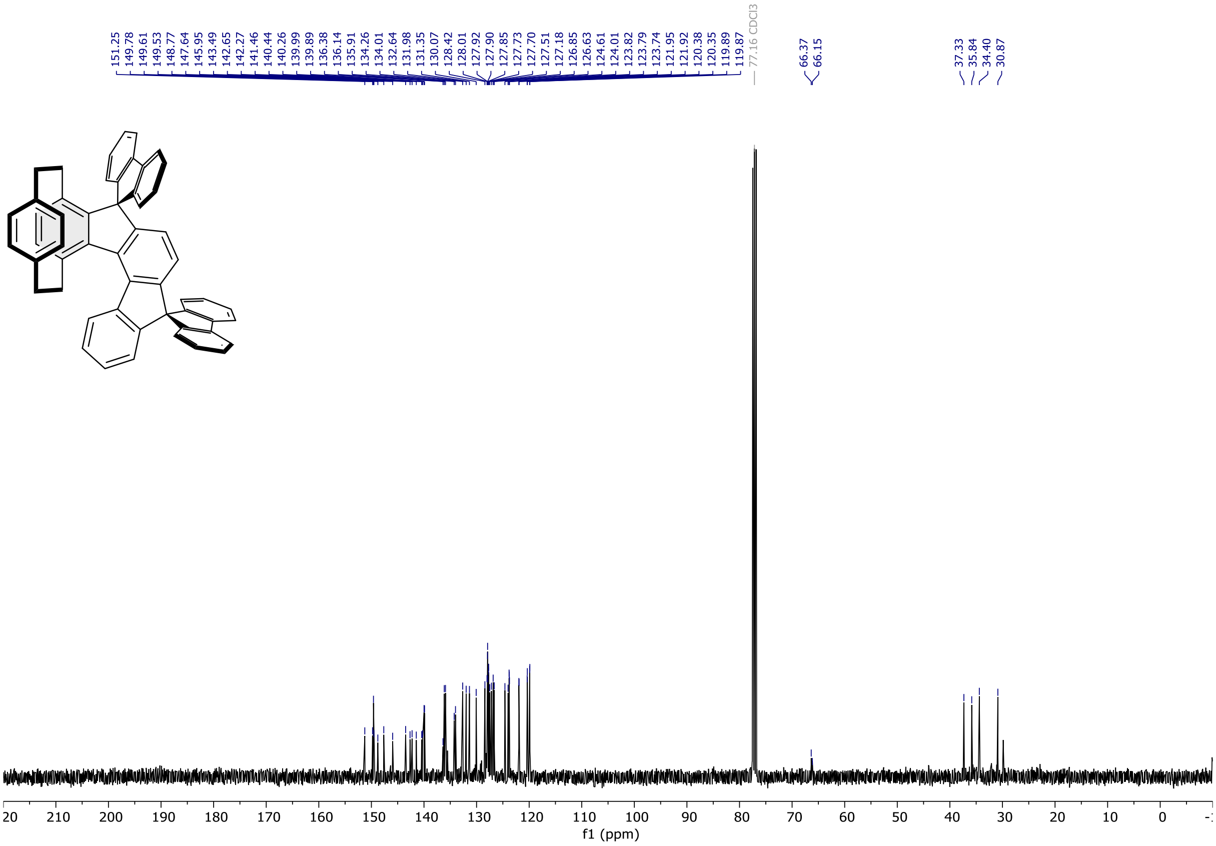

Figure S32. ^{13}C{^{1}H} NMR spectrum of compound **4** ($CDCl_3$, 101 MHz).

Bis(1,4(1,4)-dibenzenacyclohexaphane-1^2-carbaldehyde)-1^3-yl))ethyne (5)

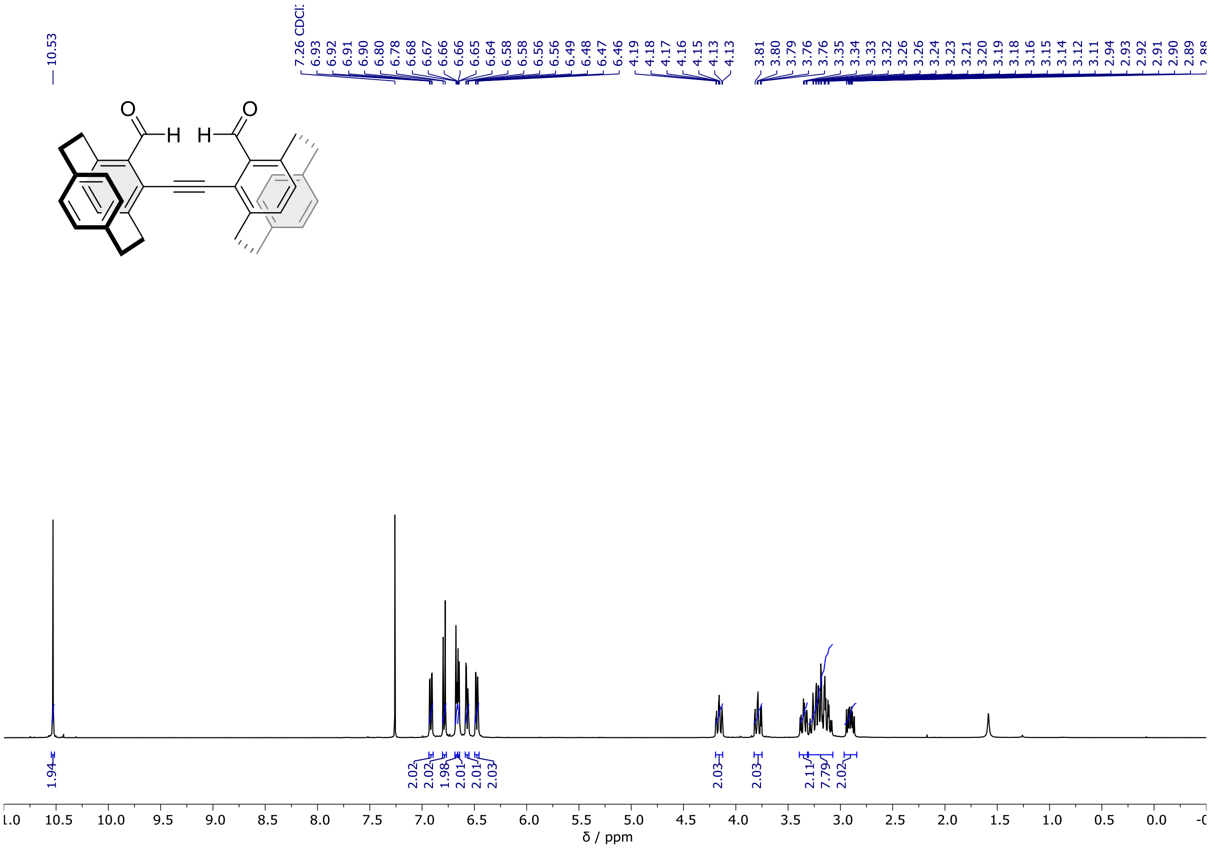

Figure S33. ^{1}H NMR spectrum of compound **5** (CDCl$_3$, 400 MHz).

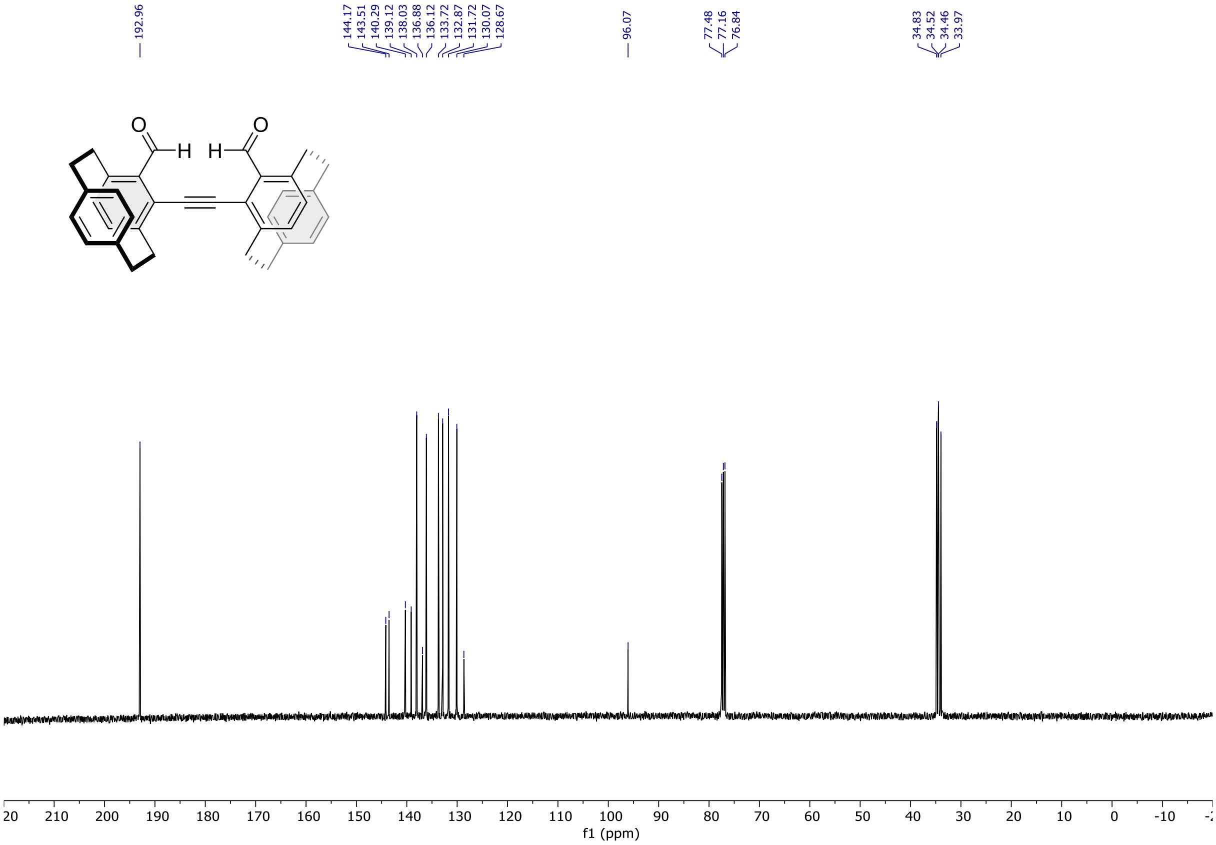

Figure S34. ^{13}C{^{1}H} NMR spectrum of compound **5** (CDCl$_3$, 101 MHz).

***meso*-Bis(1,4(1,4)-dibenzenacyclohexaphane-1²-carbaldehyde)-1³-yl))ethyne (*meso*-5)**

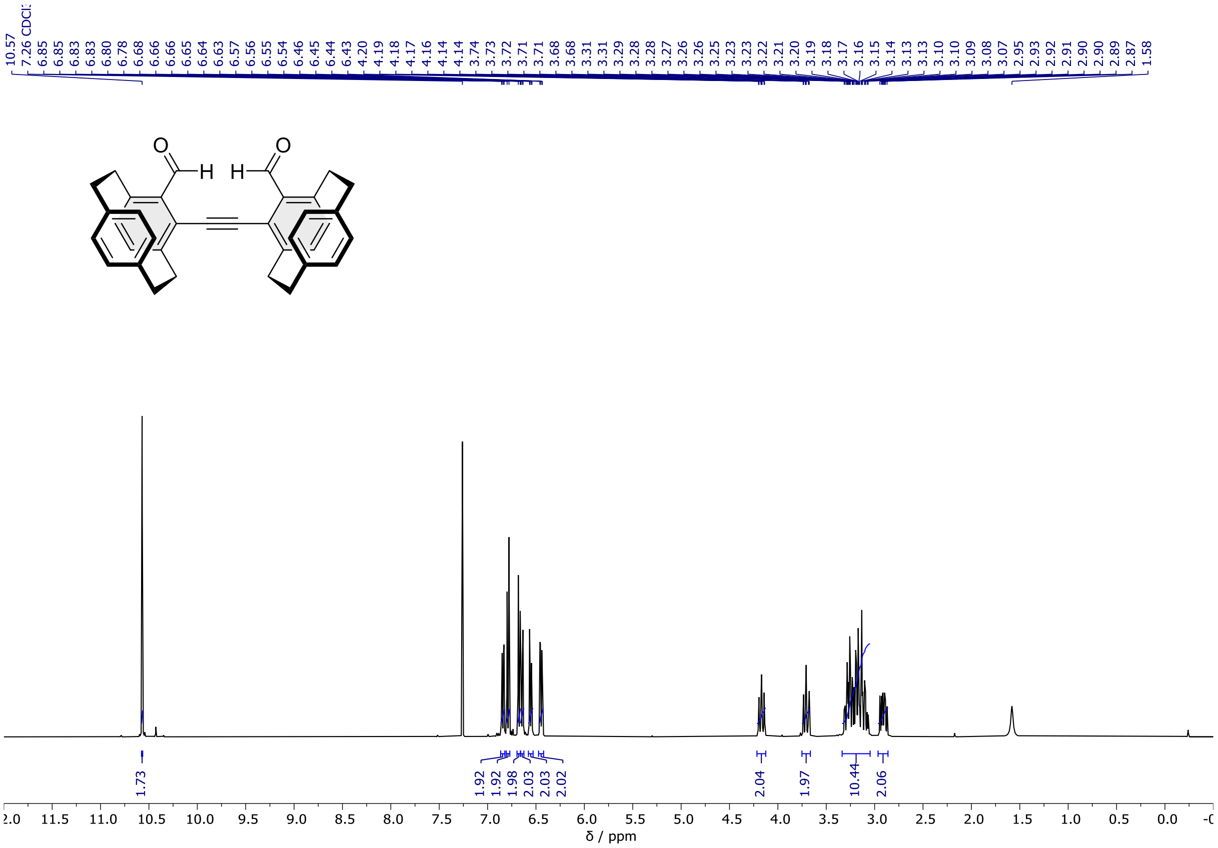

Figure S35. ^{1}H NMR spectrum of compound *meso*-**5** ($CDCl_3$, 400 MHz).

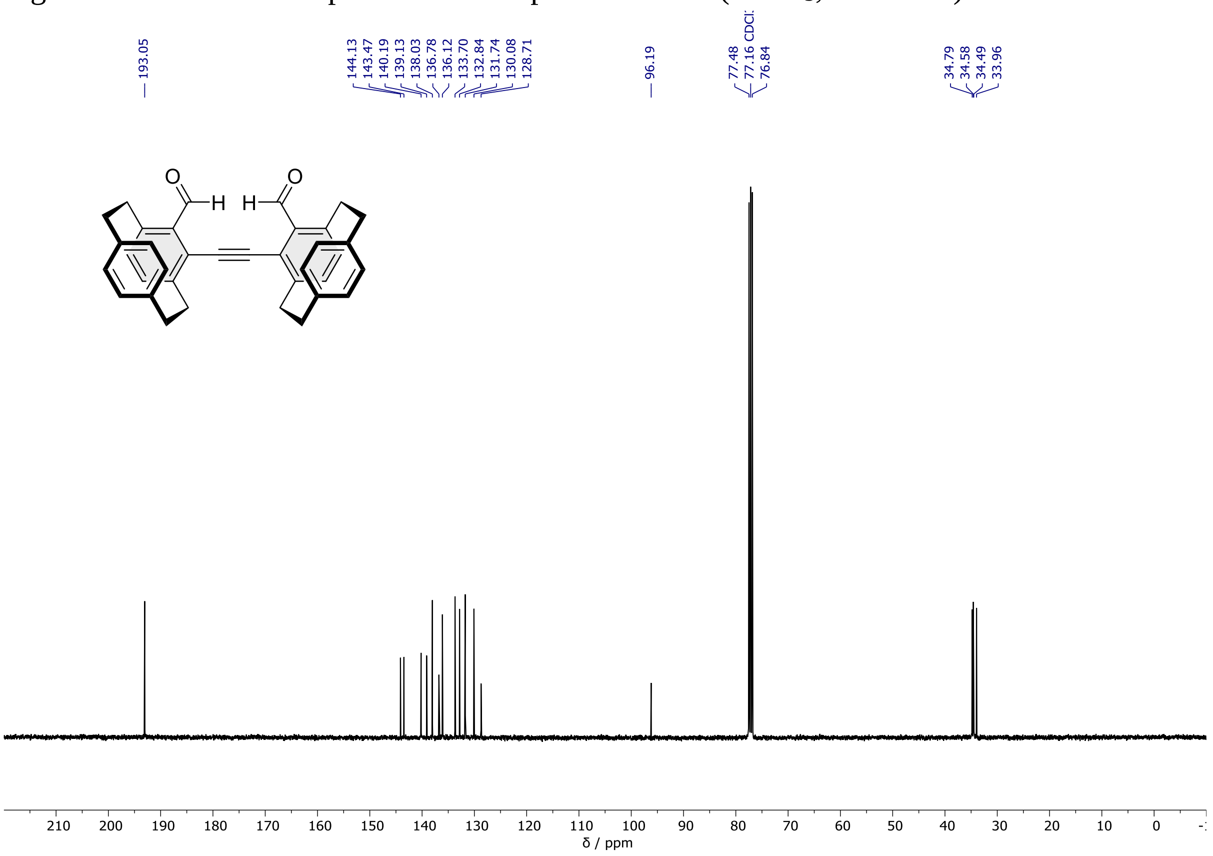

Figure S36. ^{13}C{^{1}H} NMR spectrum of compound *meso*-**5** ($CDCl_3$, 101 MHz).

Bis((1-(1,4(1,4)-dibenzenacyclohexaphane-1²-yl)prop-2-yn-1-ol)-1³-yl)ethyne (6)

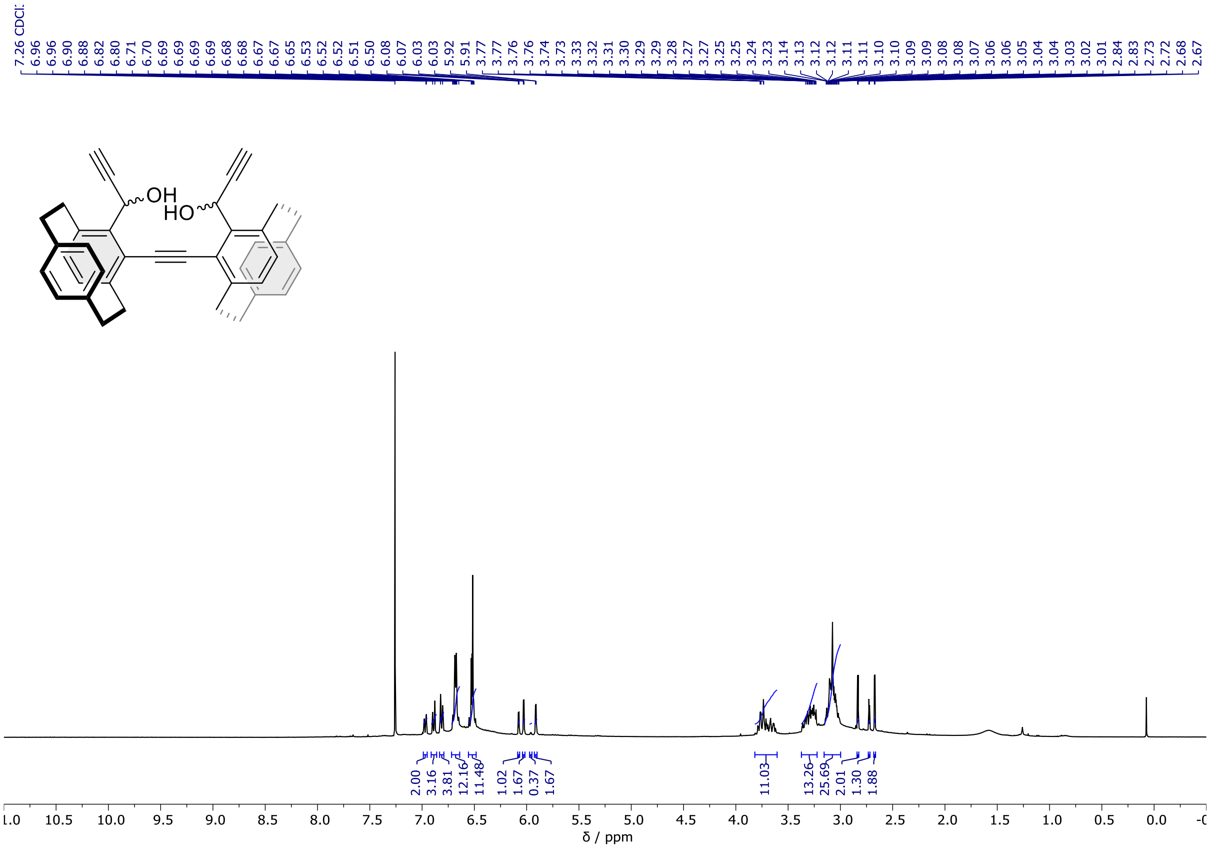

Figure S37. ^{1}H NMR spectrum of compound **6** ($CDCl_3$, 400 MHz).

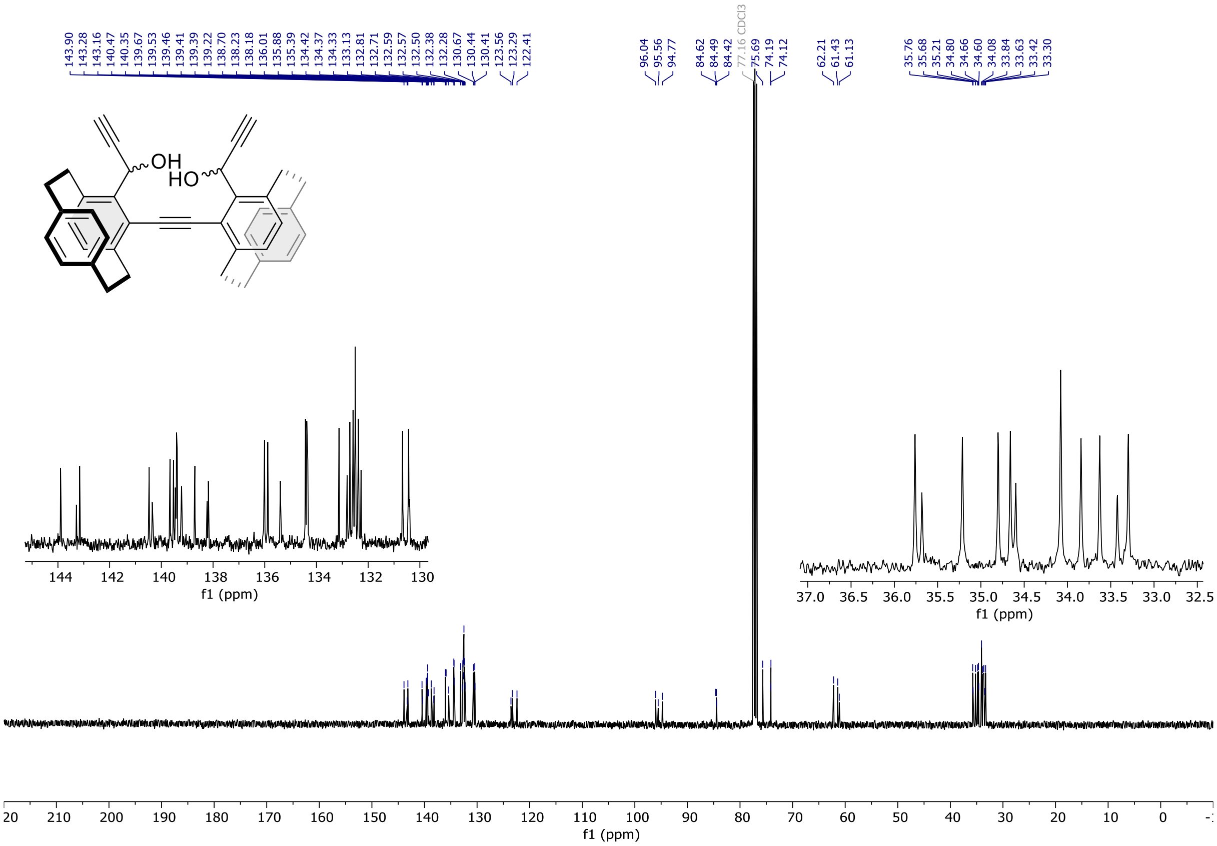

Figure S38. $^{13}C\{^{1}H\}$ NMR spectrum of compound **6** ($CDCl_3$, 101 MHz).

Bis((1-(1,4(1,4)-dibenzenacyclohexaphane-1²-yl)prop-2-yn-1-ol)-1³-yl)ethyne (*meso*-6)

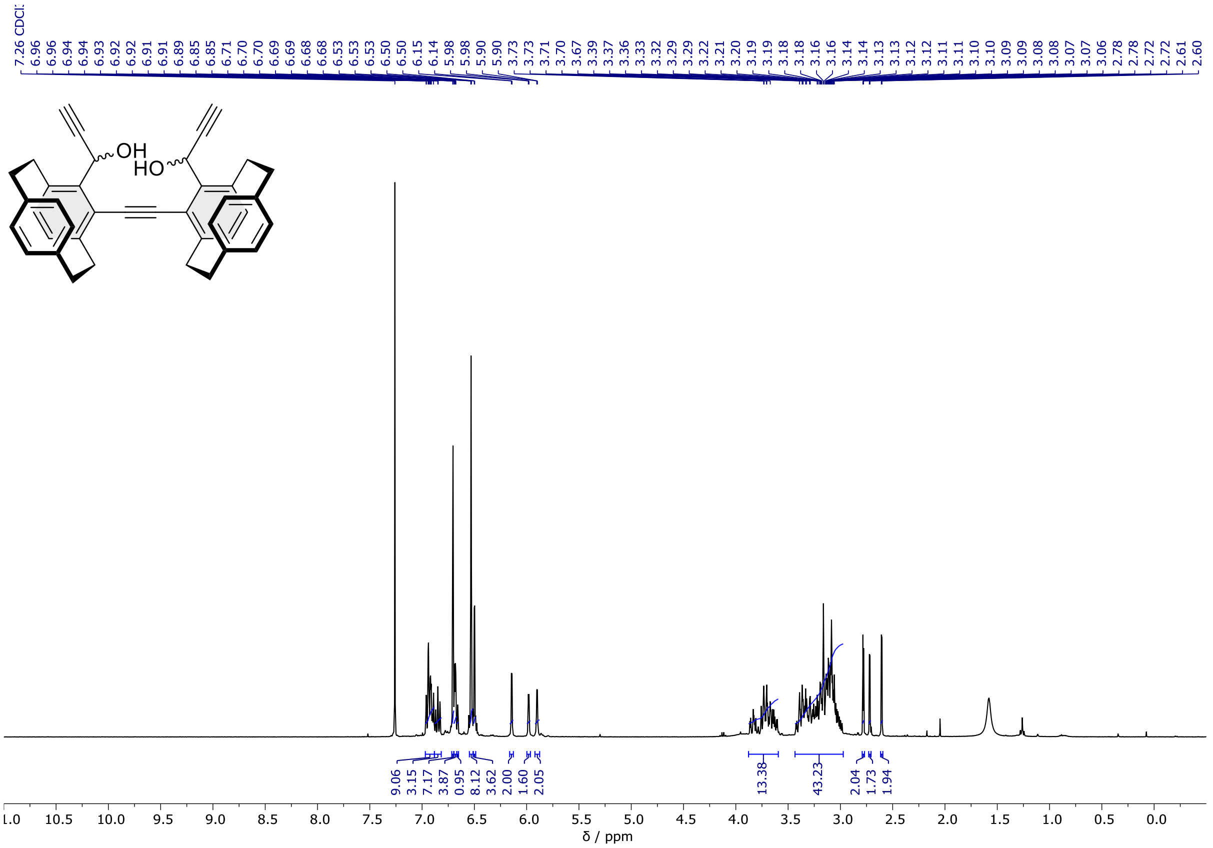

Figure S39. ^{1}H NMR spectrum of compound *meso*-**6** ($CDCl_3$, 400 MHz).

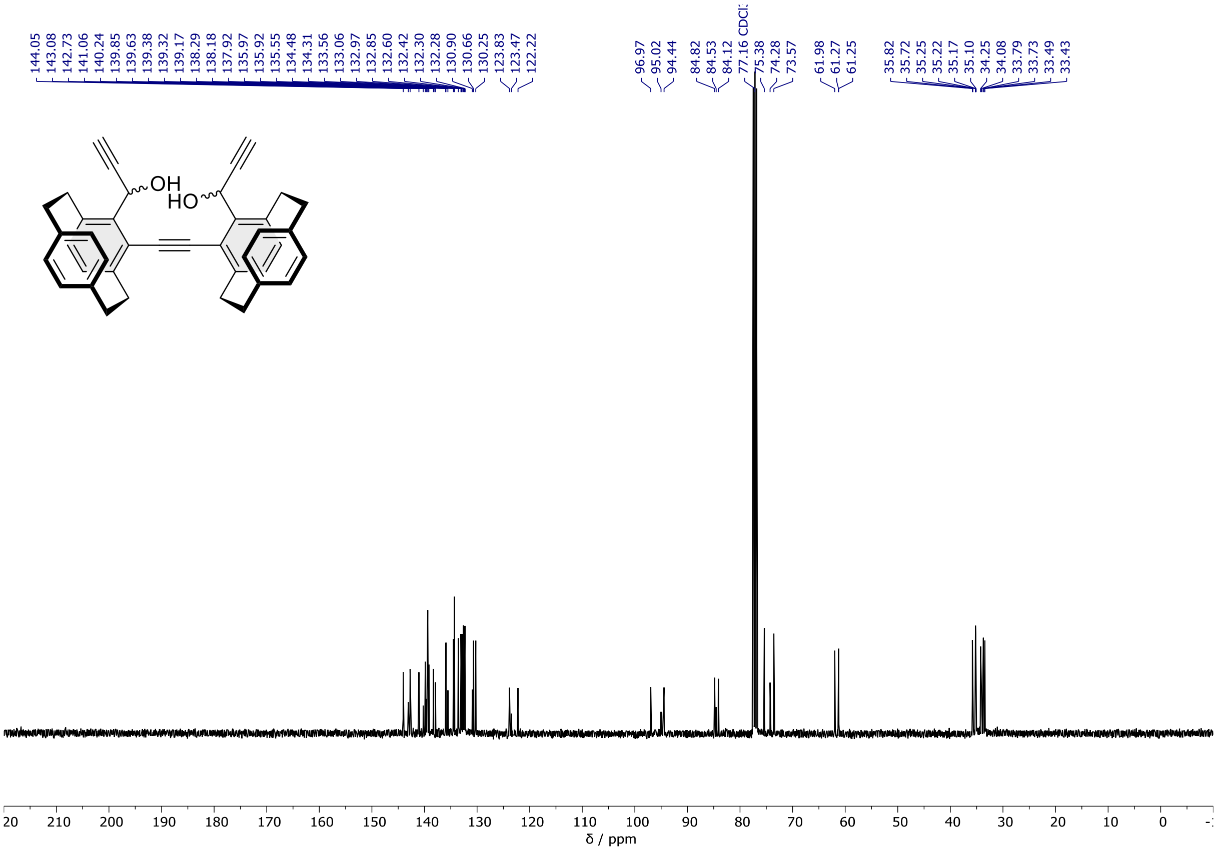

Figure S40. $^{13}C\{^{1}H\}$ NMR spectrum of compound *meso*-**6** ($CDCl_3$, 101 MHz).

2,3,8,9,16,17,22,23-octahydro-1,10:15,24-di(epiethane[1,2]diylidene)-4,7:18,21-diethenodicyclododeca[*a*,*i*]-*as*-indacene-11,14-dione (7)

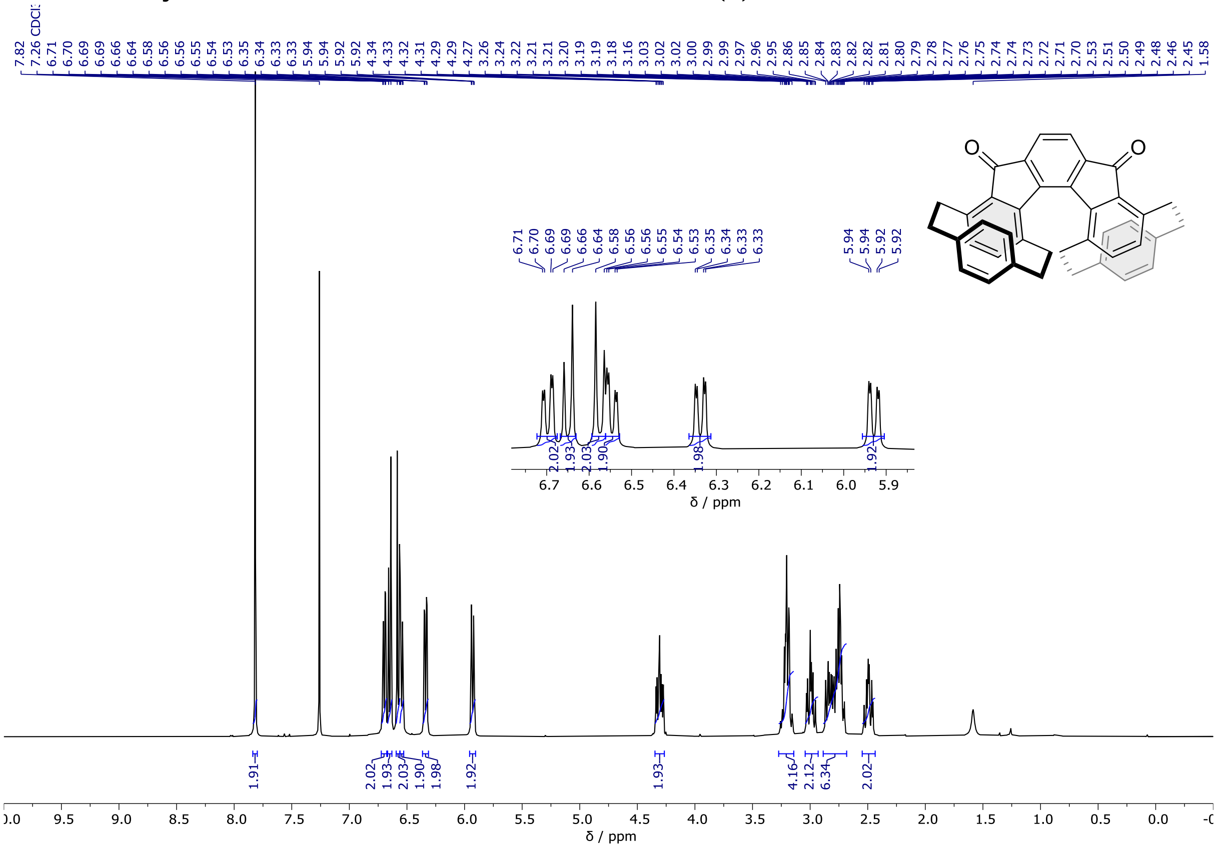

Figure S41. ^{1}H NMR spectrum of compound **7** ($CDCl_3$, 400 MHz).

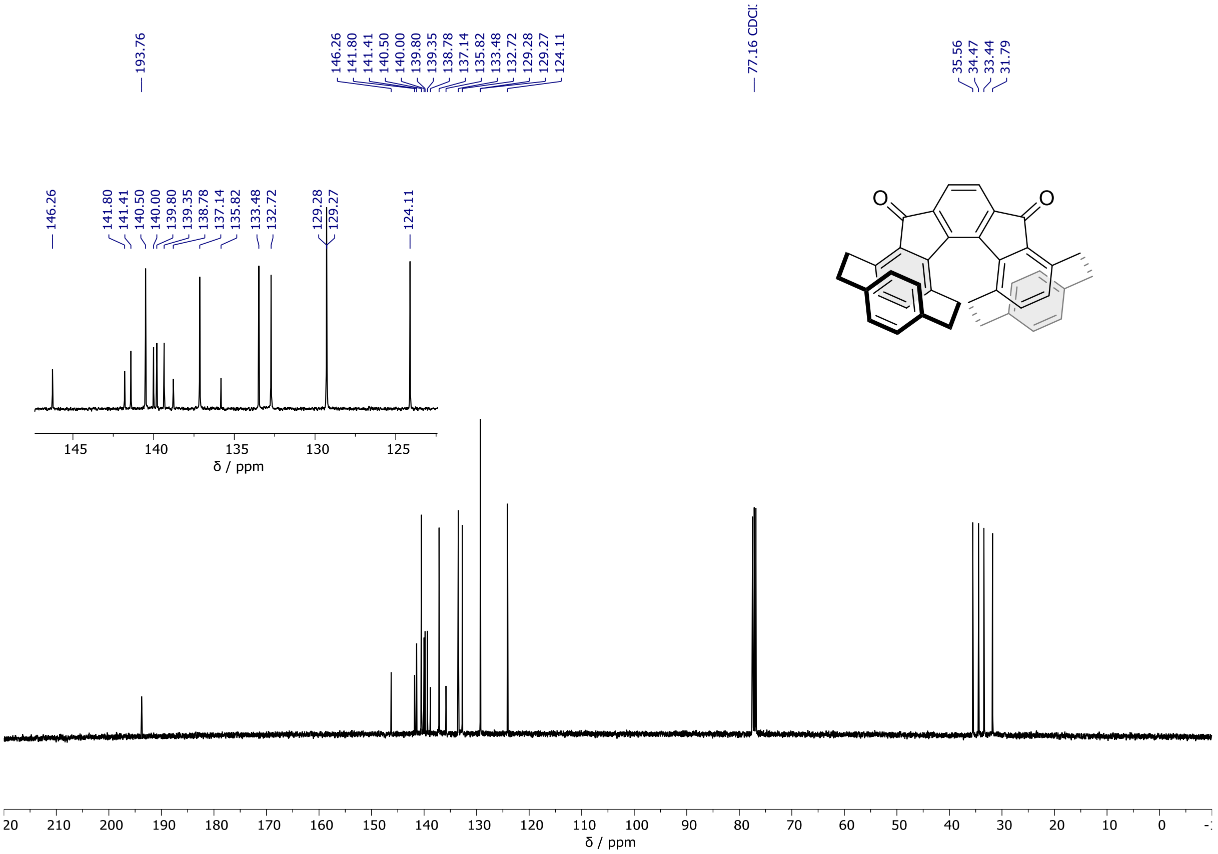

Figure S42. $^{13}C\{^{1}H\}$ NMR spectrum of compound **7** ($CDCl_3$, 101 MHz).

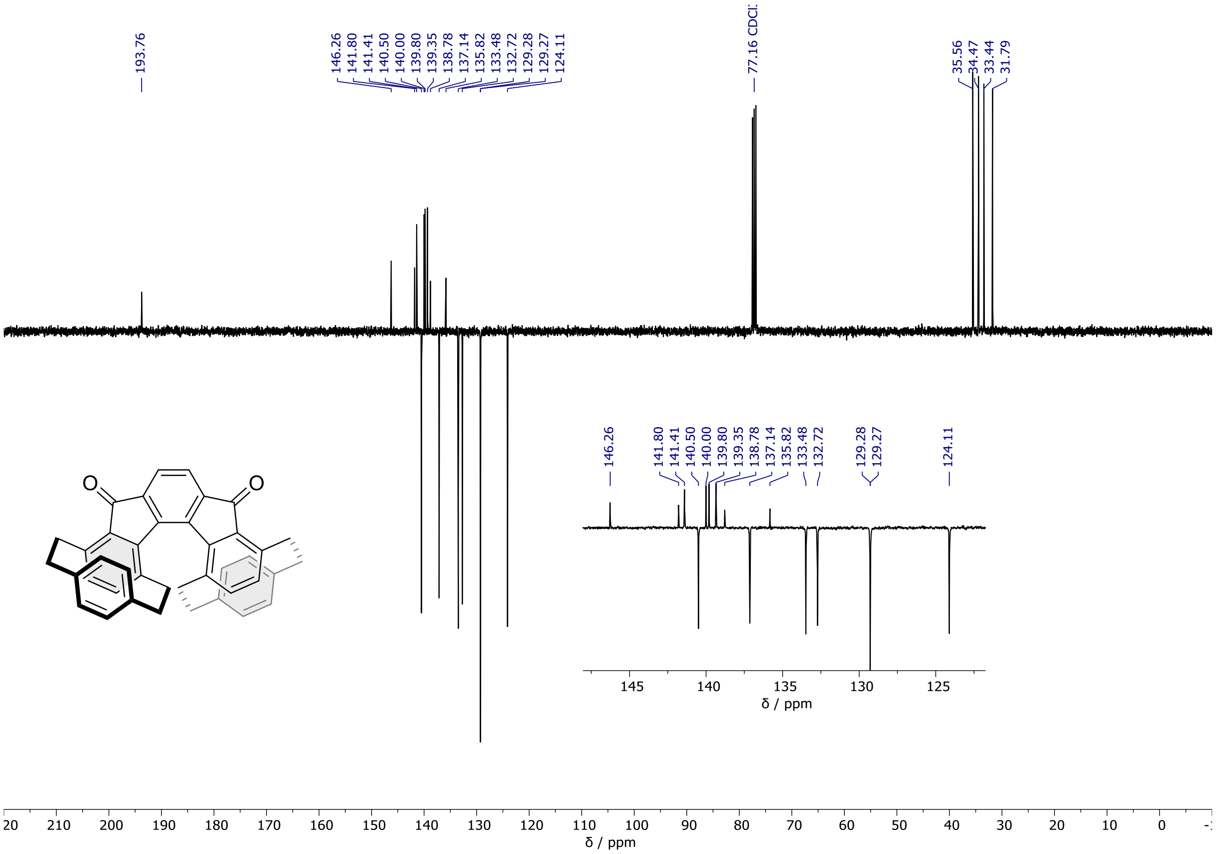

Figure S43. $^{13}C\{^{1}H\}$ APT NMR spectrum of compound **7** ($CDCl_3$, 101 MHz).

(R_P,S_P)-2,3,8,9,16,17,22,23-octahydro-1,10:15,24-di(epiethane[1,2]diylidene)-4,7:18,21-diethenodicyclododeca[*a,i*]-*as*-indacene-11,14-dione (*meso*-7).

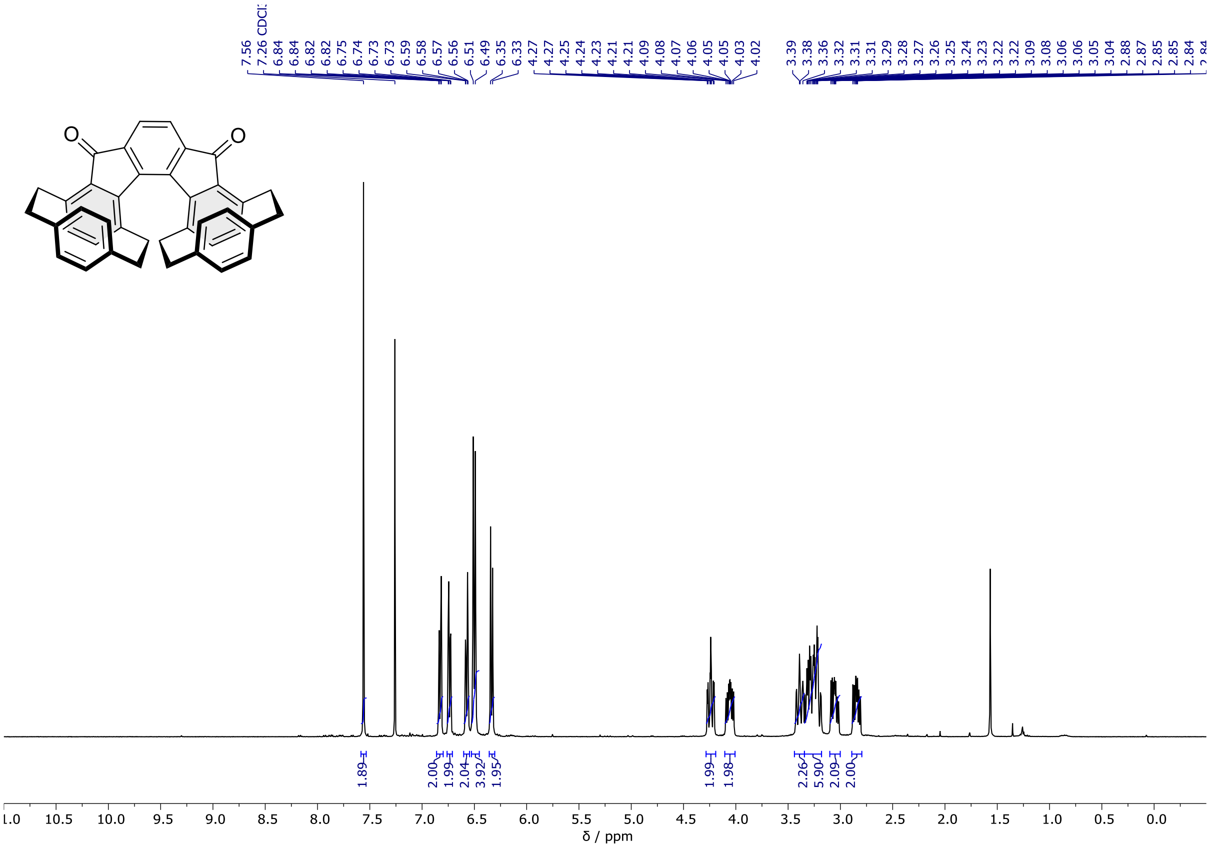

Figure S44. ^{1}H NMR spectrum of compound *meso*-**7** ($CDCl_3$, 400 MHz).

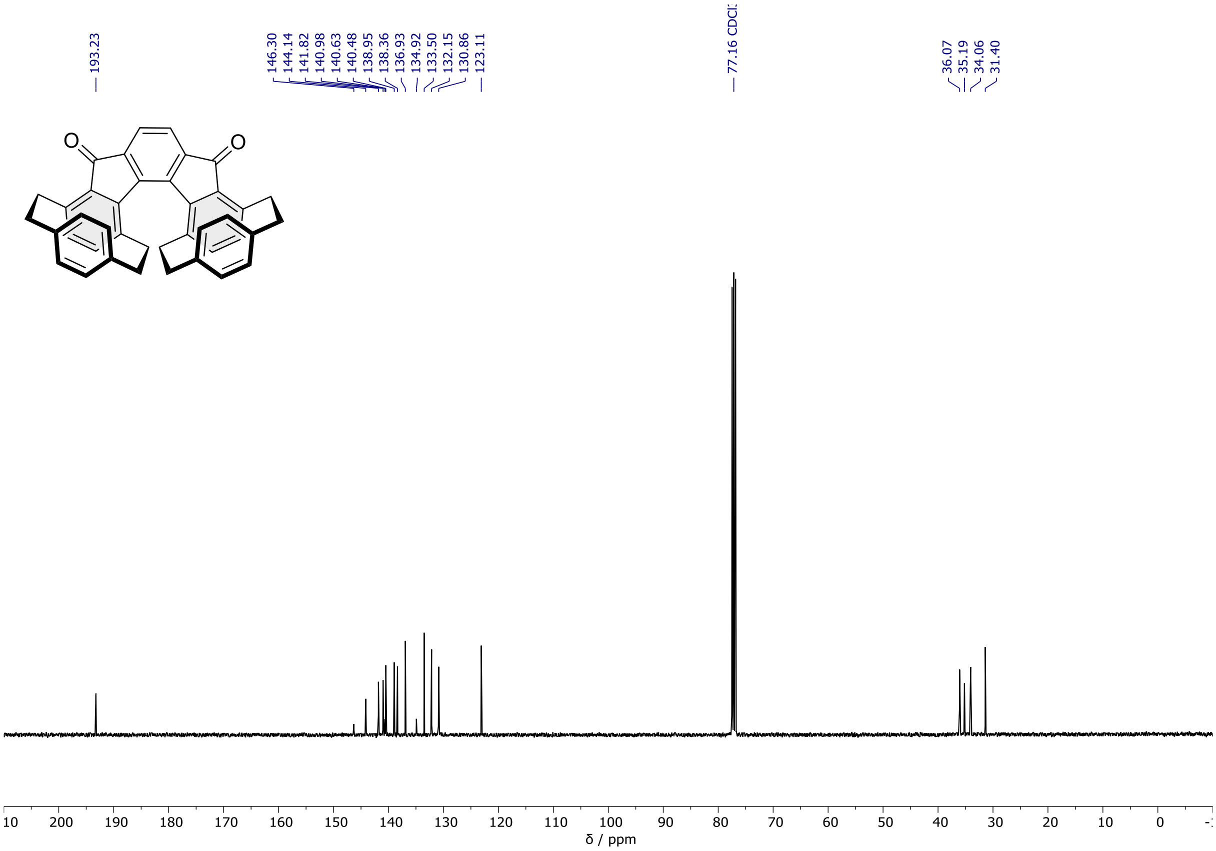

Figure S45. ^{13}C{^{1}H} NMR spectrum of compound *meso*-**7** ($CDCl_3$, 101 MHz).

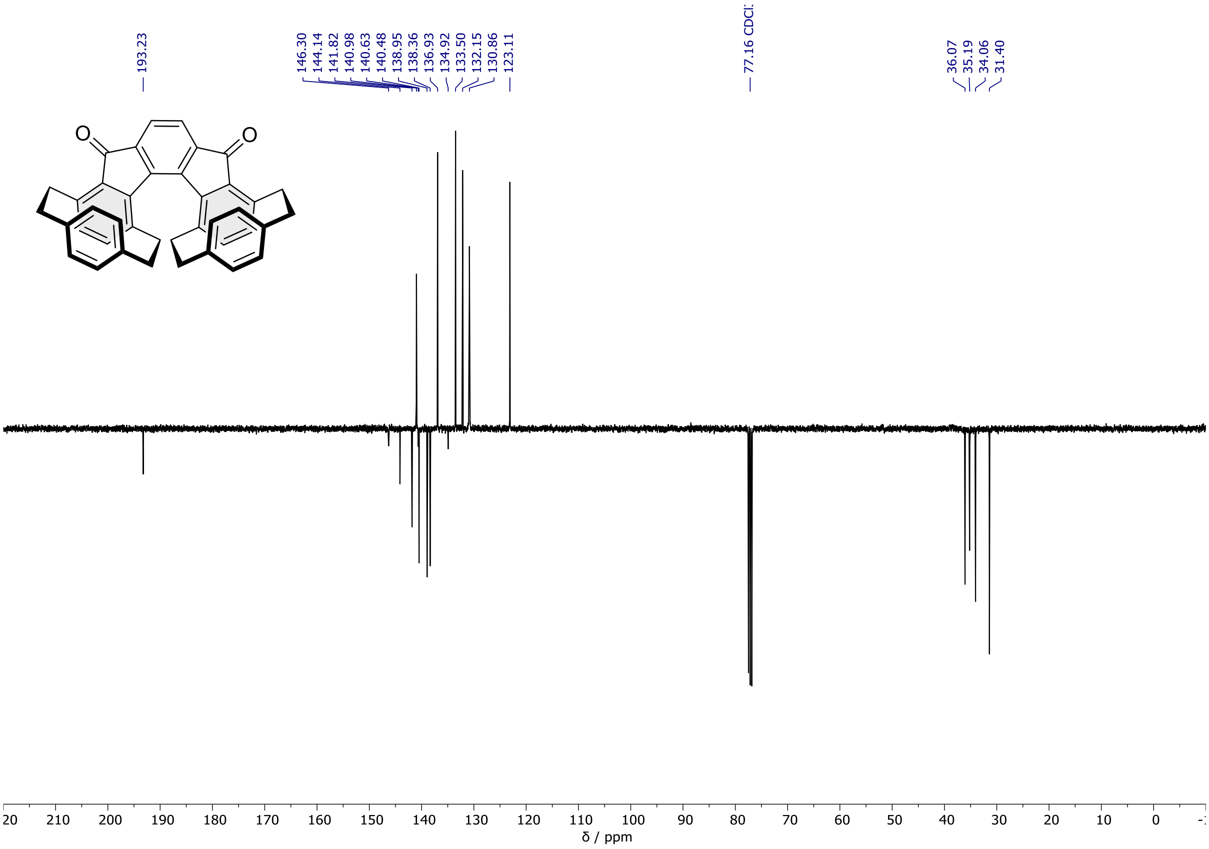

Figure S46. $^{13}C\{^{1}H\}$ APT NMR spectrum of compound *meso*-**7** ($CDCl_3$, 101 MHz).

2',3',8',9',16',17',22',23'-octahydrodispiro[fluorene-9,11'-[1,10:15,24]di(epiethane[1,2]diylidene)[4,7:18,21]diethenodicyclododeca[*a,i*]-*as*-indacene-14',9''-fluorene] (8)

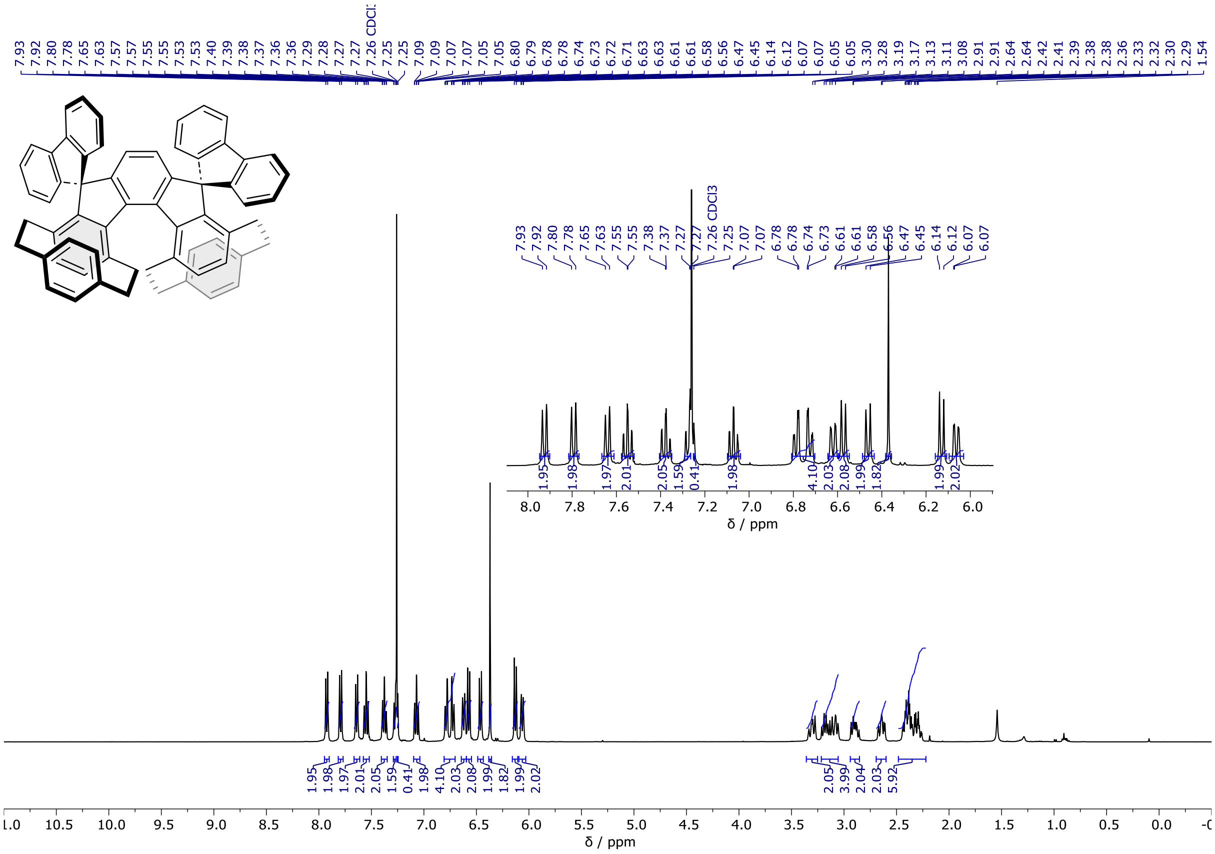

Figure S47. ^{1}H NMR spectrum of compound **8** ($CDCl_3$, 400 MHz).

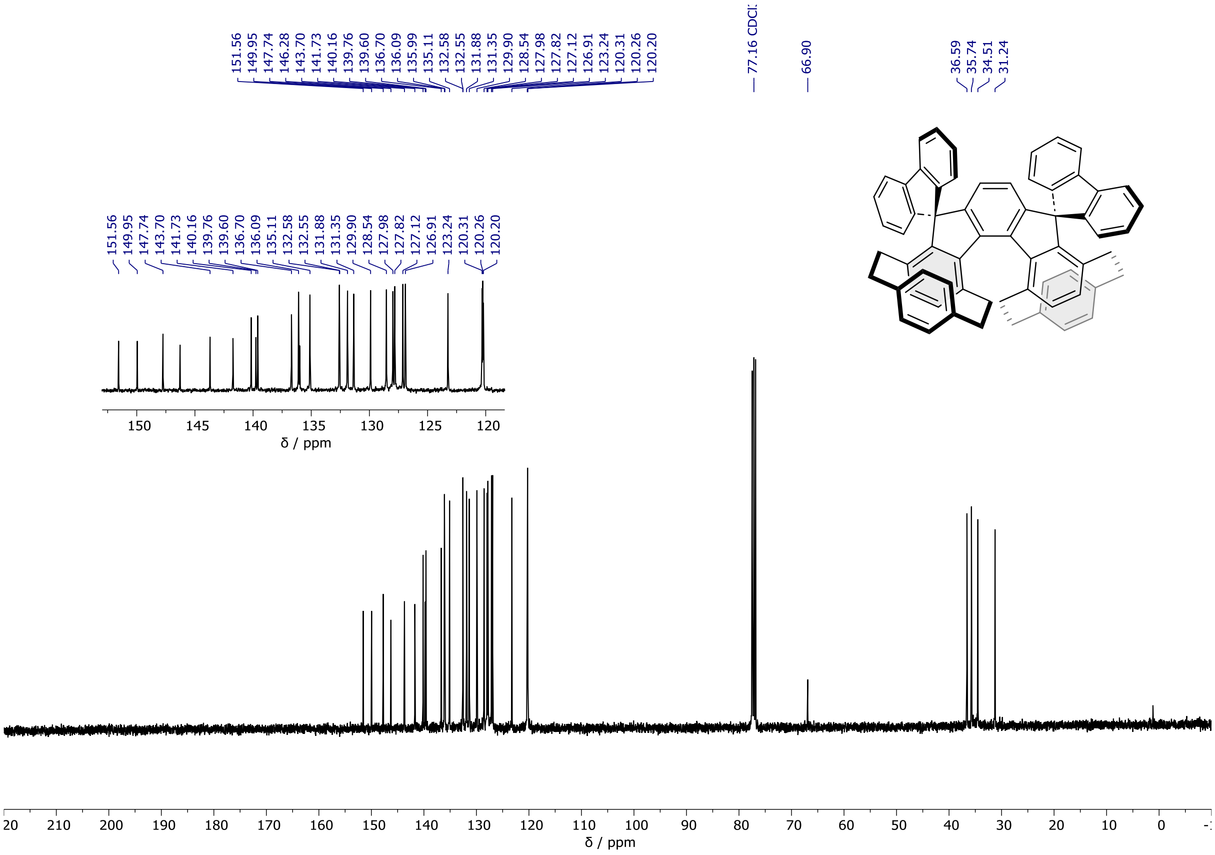

Figure S48. $^{13}C\{^{1}H\}$ NMR spectrum of compound **8** ($CDCl_3$, 101 MHz).

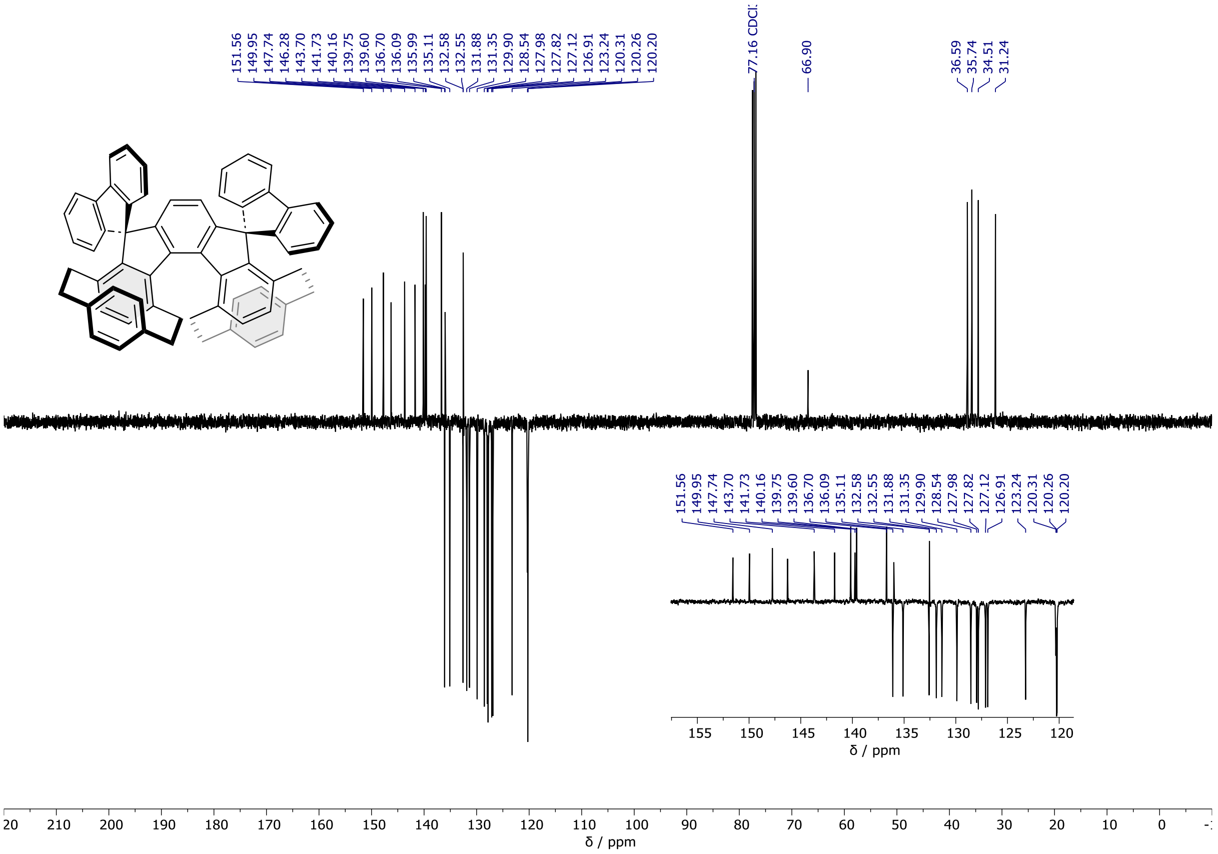

Figure S49. $^{13}C\{^{1}H\}$ APT NMR spectrum of compound **8** ($CDCl_3$, 101 MHz).

9 Selected HRMS spectra

HRMS spectrum of **4.**

Acquisition Parameter

Source Type	APPI	Ion Polarity	Positive	Set Nebulizer	2.0 Bar
Focus	Not active	Set Capillary	1500 V	Set Dry Heater	200 °C
Scan Begin	50 m/z	Set End Plate Offset	-500 V	Set Dry Gas	4.0 l/min
Scan End	1000 m/z	Set Charging Voltage	2000 V	Set Divert Valve	Waste
		Set Corona	0 nA	Set APCI Heater	450 °C

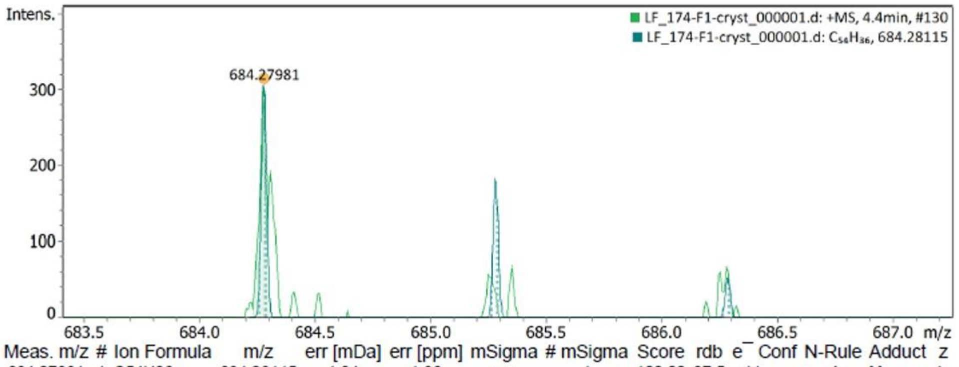

Meas. m/z	#	Ion Formula	m/z	err [mDa]	err [ppm]	mSigma	# mSigma	Score	rdb	e¯ Conf	N-Rule	Adduct	z
684.27981	1	C54H36	684.28115	1.34	1.96	n.a.	1	100.00	37.5	odd	ok	M	1+

HRMS spectrum of **8.**

Acquisition Parameter

Source Type	APPI	Ion Polarity	Positive	Set Nebulizer	2.0 Bar
Focus	Active	Set Capillary	1500 V	Set Dry Heater	200 °C
Scan Begin	50 m/z	Set End Plate Offset	-600 V	Set Dry Gas	4.0 l/min
Scan End	1400 m/z	Set Charging Voltage	2000 V	Set Divert Valve	Waste
		Set Corona	0 nA	Set APCI Heater	450 °C

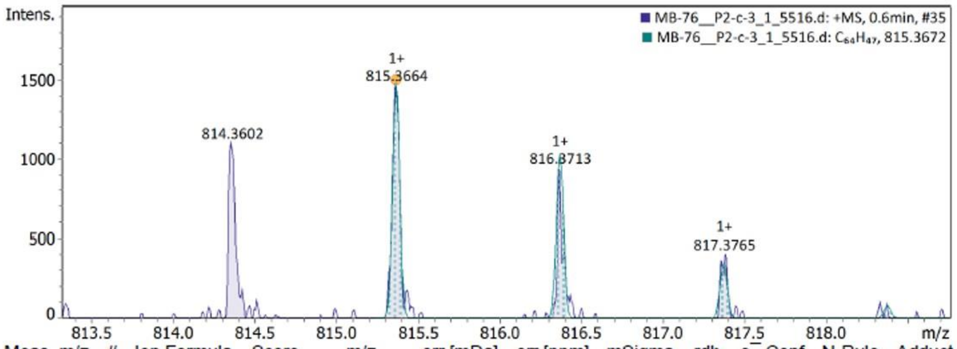

Meas. m/z	#	Ion Formula	Score	m/z	err [mDa]	err [ppm]	mSigma	rdb	e¯ Conf	N-Rule	Adduct
815.366351	1	C64H47	100.00	815.367228	0.9	1.1	38.4	42.0	even	ok	M+H
